# Supplementary figures and images for: HIF1α stabilization in hypoxia is not oxidant-initiated
Source: eLife. 2021 Oct 1;10:e72873. doi: 10.7554/eLife.72873 (PMC8530508; doi:10.7554/eLife.72873)

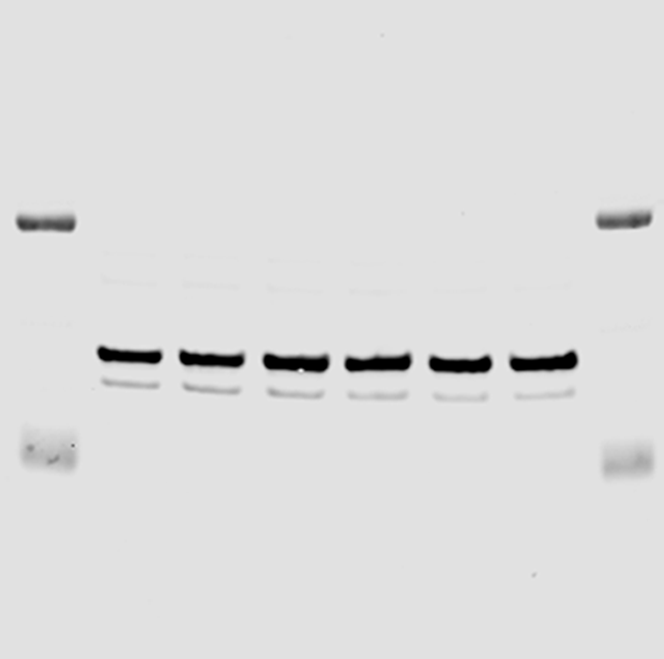

Supplement: Figure 1—source data 1. [file elife-72873-fig1-data1.zip › Figure 1 - source data 1/Actin blot.tif]

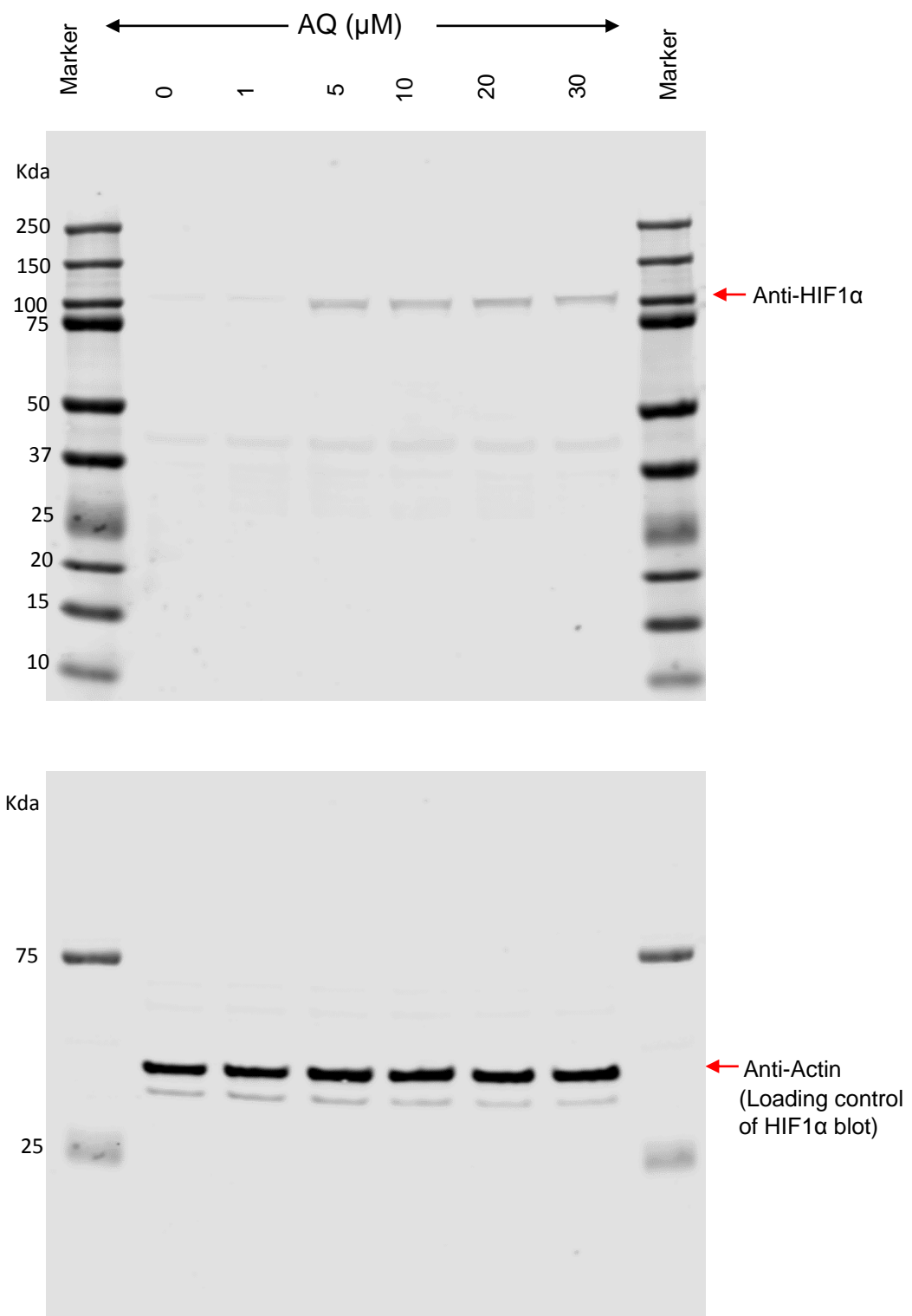

**Figure 1A**

Supplement: Figure 1—source data 1. [file elife-72873-fig1-data1.zip › Figure 1 - source data 1/Figure 1 - source data 1.pdf]

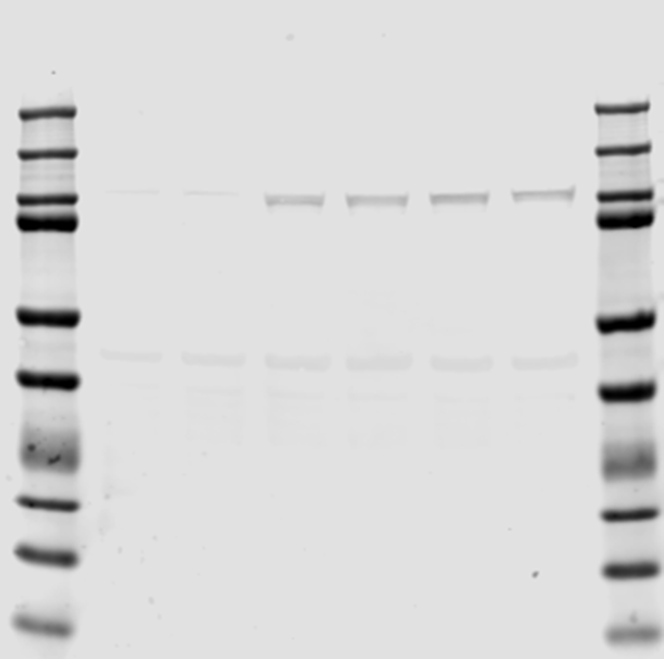

Supplement: Figure 1—source data 1. [file elife-72873-fig1-data1.zip › Figure 1 - source data 1/HIF1alpha blot.tif]

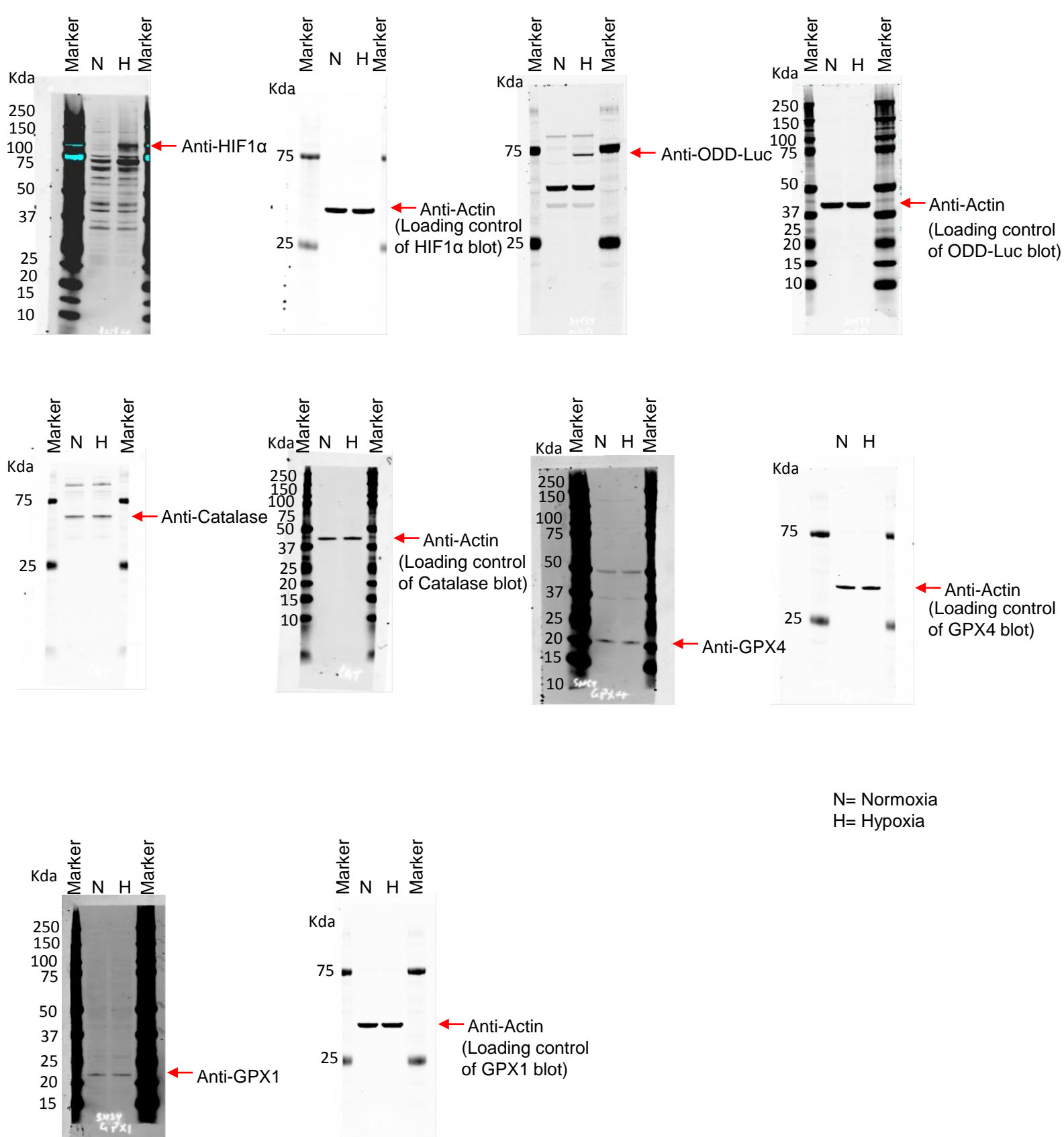

**Figure 2A**

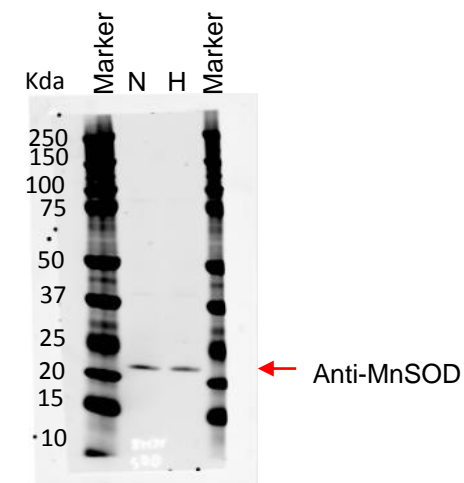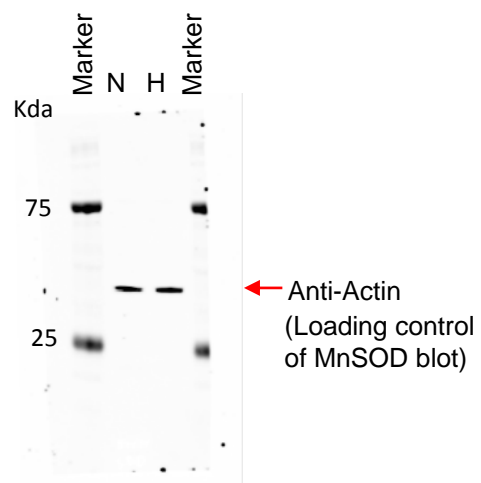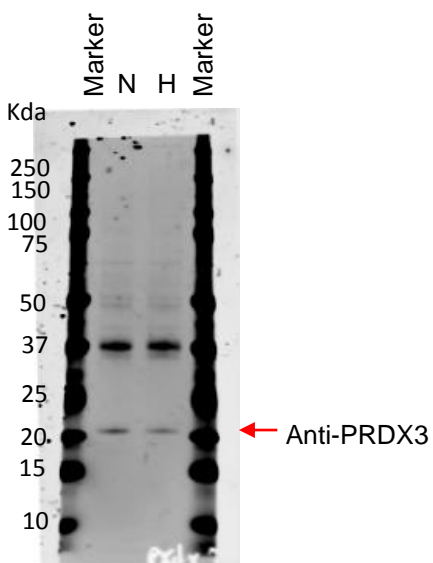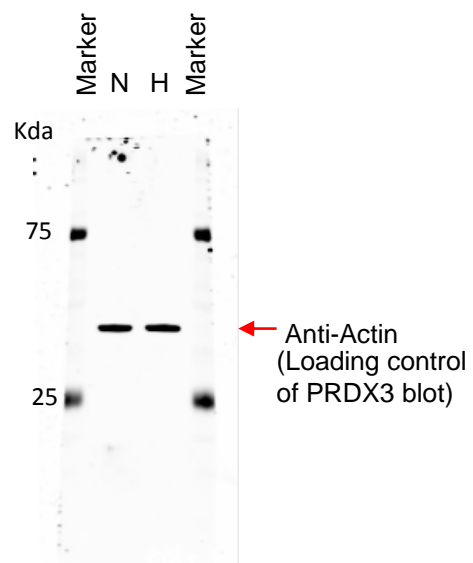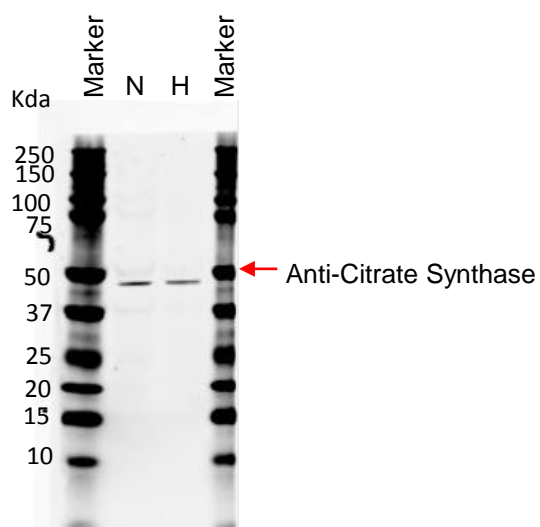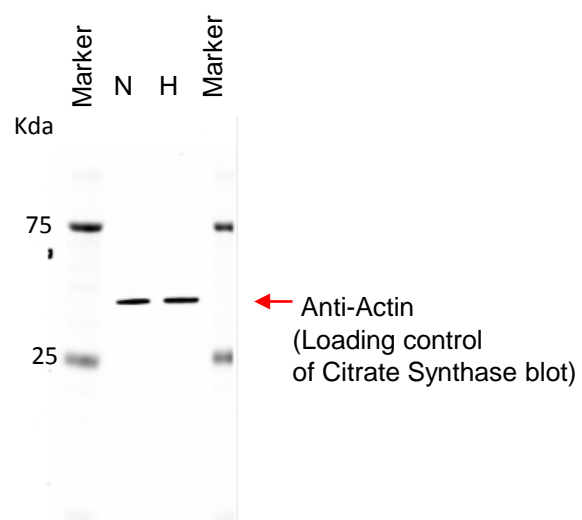

N= Normoxia  
H= Hypoxia

**Figure 2D**

Supplement: Figure 2—source data 1. [file elife-72873-fig2-data1.zip › Figure 2 - source data 1/Figure 2 - source data 1.pdf]

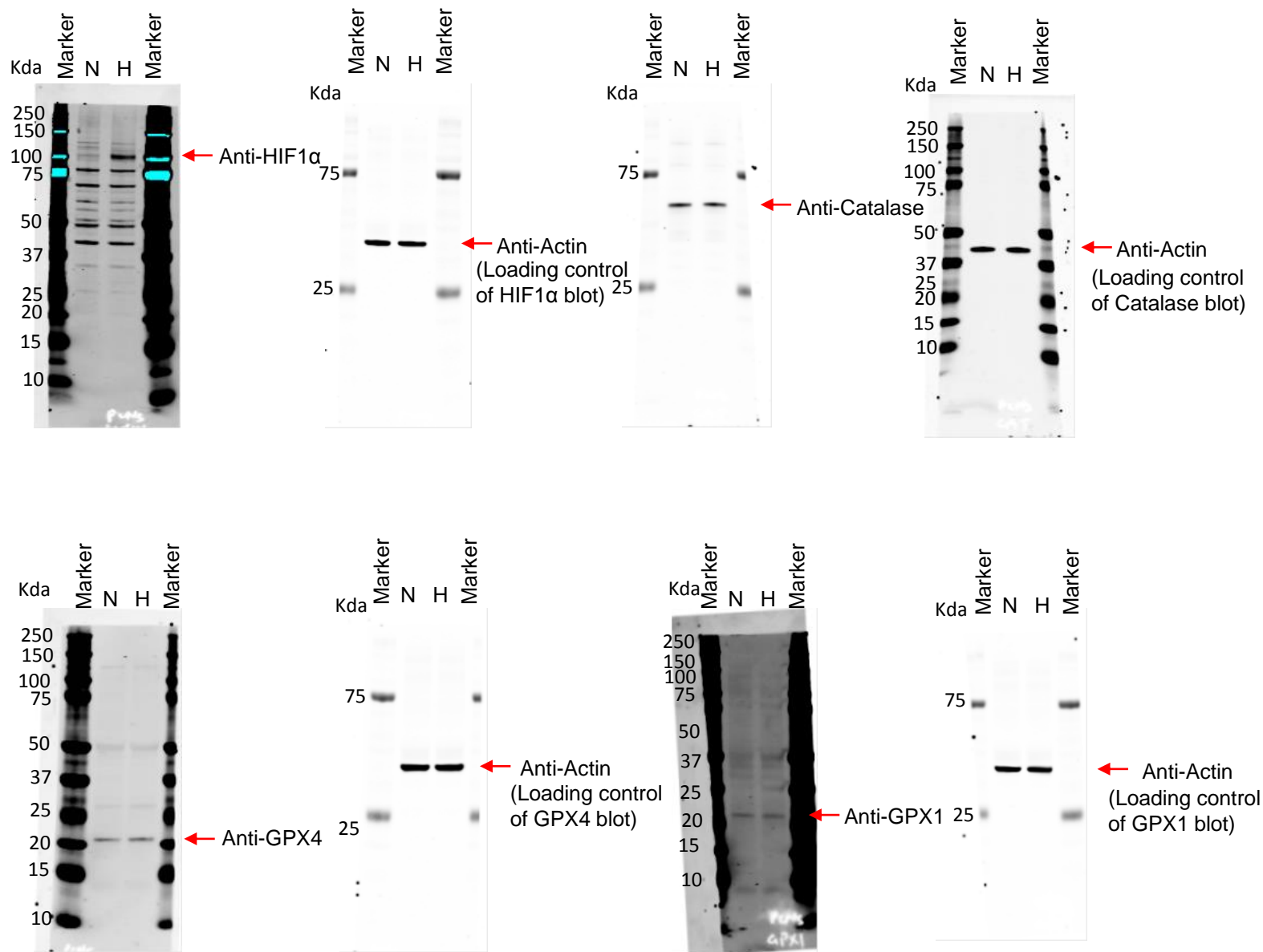

**Figure 2F**

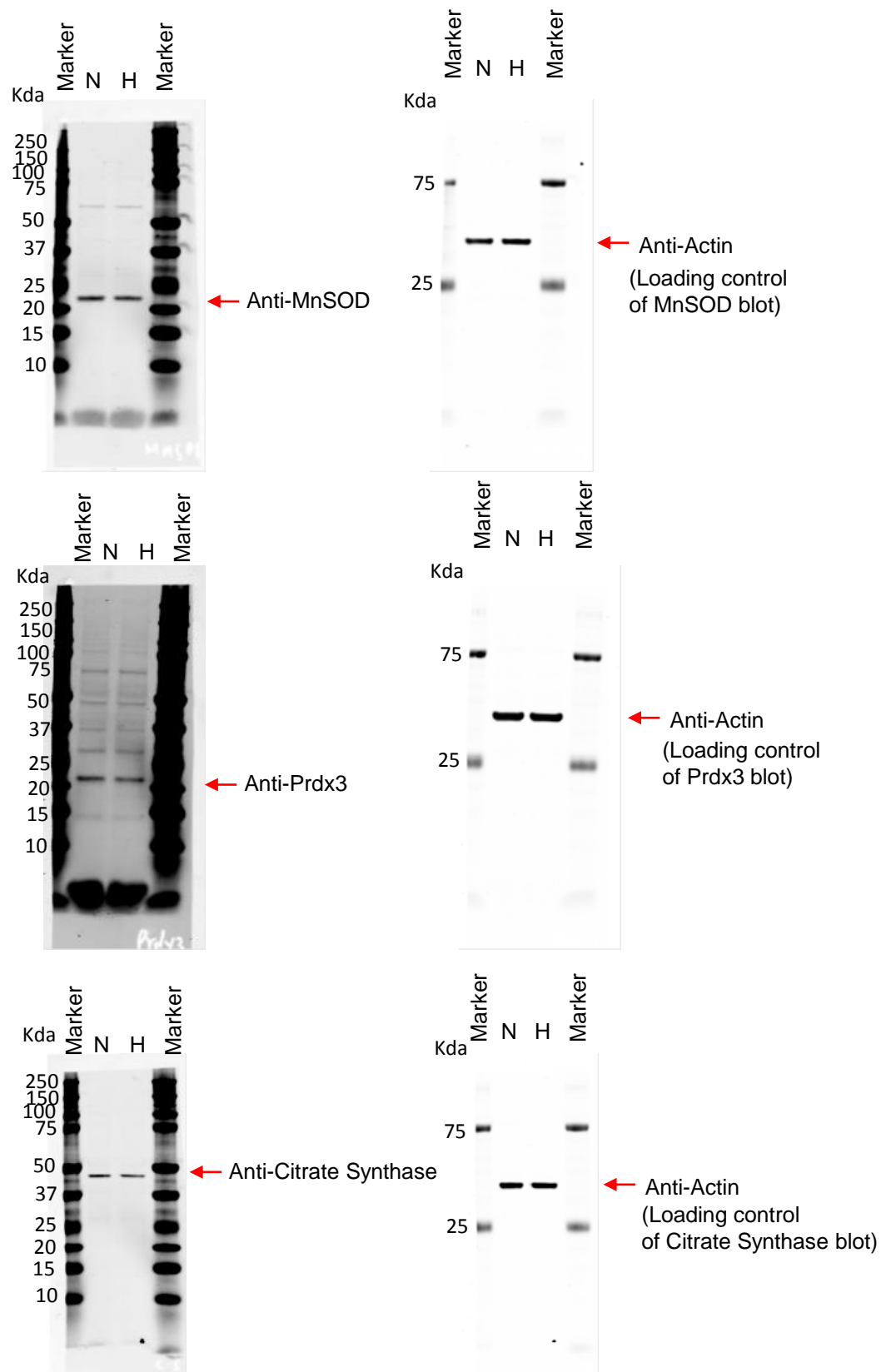

**Figure 2H**

Supplement: Figure 2—source data 2. [file elife-72873-fig2-data2.zip › Figure 2 - source data 2/Figure 2 - source data 2.pdf]

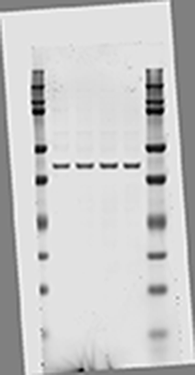

Supplement: Figure 2—figure supplement 1—source data 1. [file elife-72873-fig2-figsupp1-data1.zip › Figure S1 - source data 1/Actin control of catalase blot.tif]

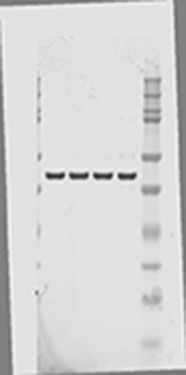

Supplement: Figure 2—figure supplement 1—source data 1. [file elife-72873-fig2-figsupp1-data1.zip › Figure S1 - source data 1/Actin control of Citrate Synthase blot.tif]

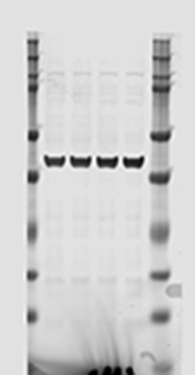

Supplement: Figure 2—figure supplement 1—source data 1. [file elife-72873-fig2-figsupp1-data1.zip › Figure S1 - source data 1/Actin control of GPX1 blot.tif]

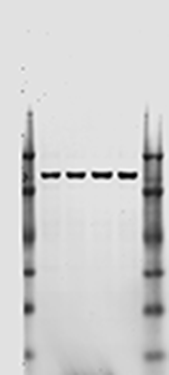

Supplement: Figure 2—figure supplement 1—source data 1. [file elife-72873-fig2-figsupp1-data1.zip › Figure S1 - source data 1/Actin control of GPX4 blot.tif]

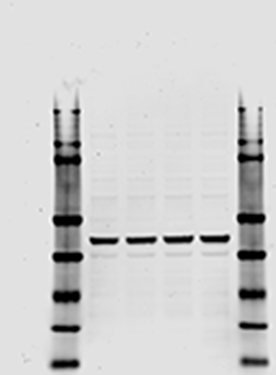

Supplement: Figure 2—figure supplement 1—source data 1. [file elife-72873-fig2-figsupp1-data1.zip › Figure S1 - source data 1/Actin control of HIF1alpha blot.tif]

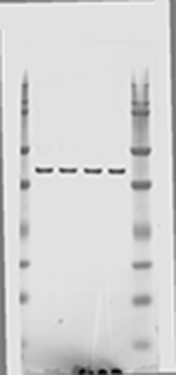

Supplement: Figure 2—figure supplement 1—source data 1. [file elife-72873-fig2-figsupp1-data1.zip › Figure S1 - source data 1/Actin control of MnSOD blot.tif]

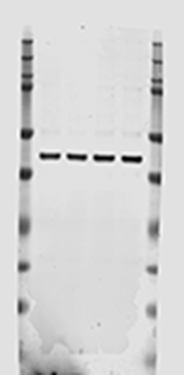

Supplement: Figure 2—figure supplement 1—source data 1. [file elife-72873-fig2-figsupp1-data1.zip › Figure S1 - source data 1/Actin control of PRDX3 blot.tif]

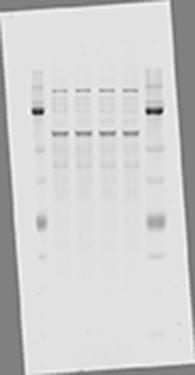

Supplement: Figure 2—figure supplement 1—source data 1. [file elife-72873-fig2-figsupp1-data1.zip › Figure S1 - source data 1/Catalase blot.tif]

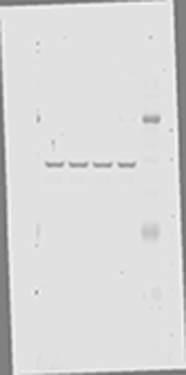

Supplement: Figure 2—figure supplement 1—source data 1. [file elife-72873-fig2-figsupp1-data1.zip › Figure S1 - source data 1/Citrate Synthase blot.tif]

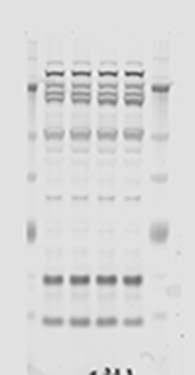

Supplement: Figure 2—figure supplement 1—source data 1. [file elife-72873-fig2-figsupp1-data1.zip › Figure S1 - source data 1/GPX1 blot.tif]

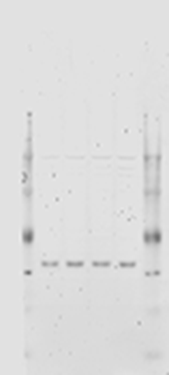

Supplement: Figure 2—figure supplement 1—source data 1. [file elife-72873-fig2-figsupp1-data1.zip › Figure S1 - source data 1/GPX4 blot.tif]

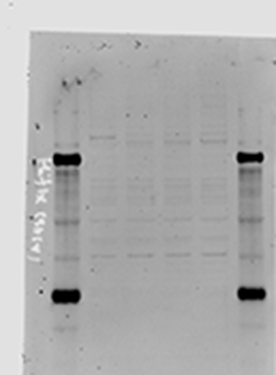

Supplement: Figure 2—figure supplement 1—source data 1. [file elife-72873-fig2-figsupp1-data1.zip › Figure S1 - source data 1/HIF1alpha blot.tif]

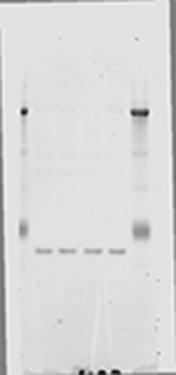

Supplement: Figure 2—figure supplement 1—source data 1. [file elife-72873-fig2-figsupp1-data1.zip › Figure S1 - source data 1/MnSOD blot.tif]

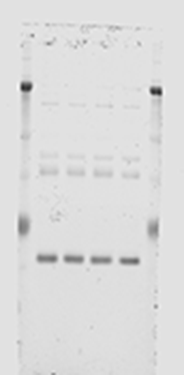

Supplement: Figure 2—figure supplement 1—source data 1. [file elife-72873-fig2-figsupp1-data1.zip › Figure S1 - source data 1/PRDX3 blot.tif]

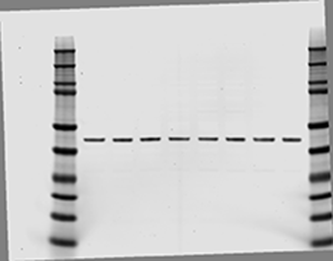

Supplement: Figure 3—source data 1. [file elife-72873-fig3-data1.zip › Figure 3 - source data 1/Actin control of catalase blot.tif]

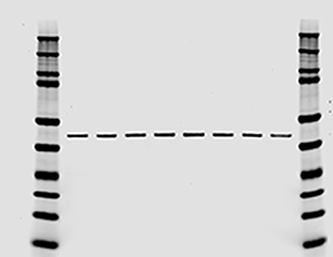

Supplement: Figure 3—source data 1. [file elife-72873-fig3-data1.zip › Figure 3 - source data 1/Actin control of GFP blot.tif]

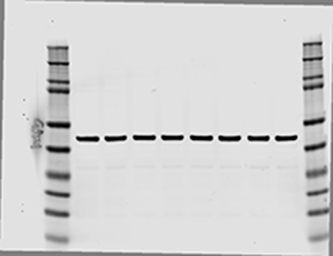

Supplement: Figure 3—source data 1. [file elife-72873-fig3-data1.zip › Figure 3 - source data 1/Actin control of GPX1 blot.tif]

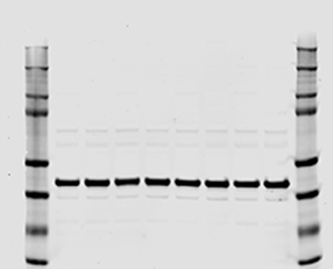

Supplement: Figure 3—source data 1. [file elife-72873-fig3-data1.zip › Figure 3 - source data 1/Actin control of HIF1alpha blot.tif]

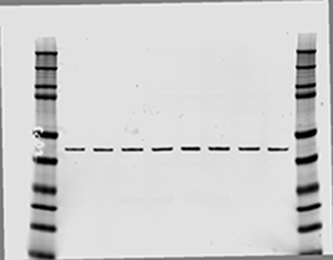

Supplement: Figure 3—source data 1. [file elife-72873-fig3-data1.zip › Figure 3 - source data 1/Actin control of MnSOD blot.tif]

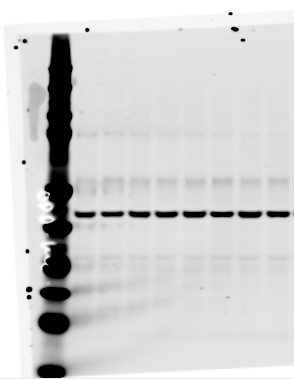

Supplement: Figure 3—source data 1. [file elife-72873-fig3-data1.zip › Figure 3 - source data 1/Actin control of ODD-Luc blot.tif]

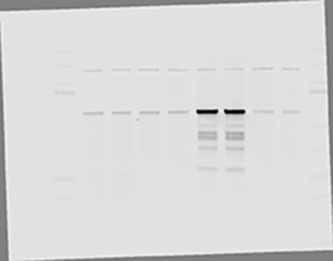

Supplement: Figure 3—source data 1. [file elife-72873-fig3-data1.zip › Figure 3 - source data 1/Catalase blot.tif]

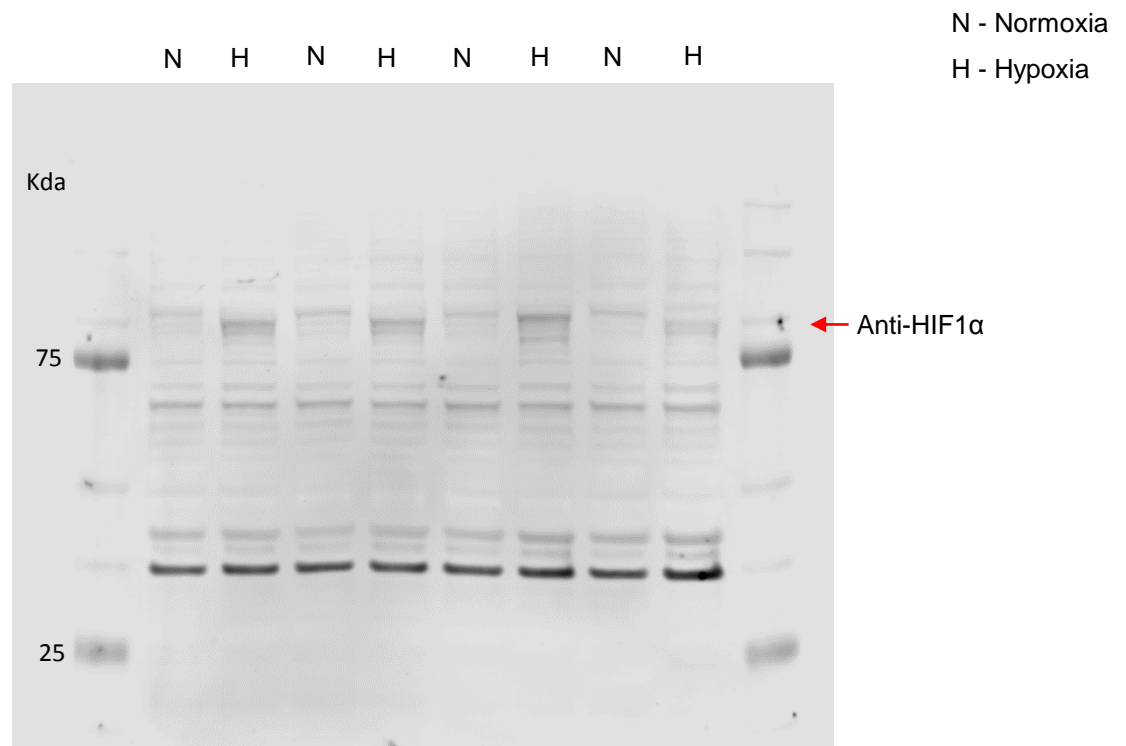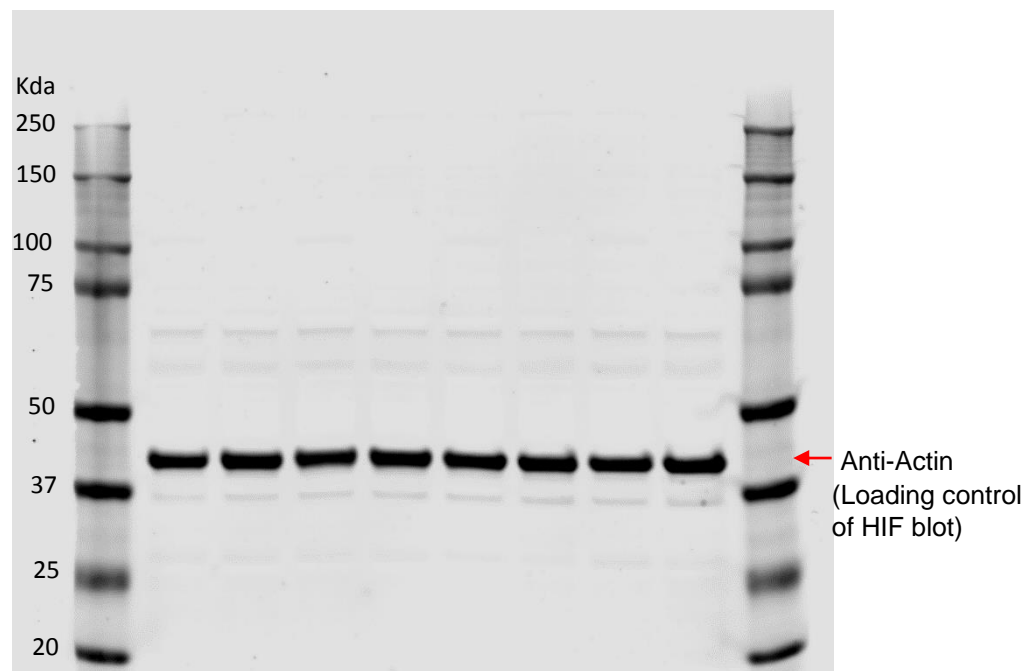

|          |   |   |   |   |   |   |   |   |
|----------|---|---|---|---|---|---|---|---|
| GFP      | + | + | - | - | - | - | - | - |
| MnSOD    | - | - | + | + | - | - | - | - |
| Catalase | - | - | - | - | + | + | - | - |
| GPX1     | - | - | - | - | - | - | + | + |

**Figure 3C**

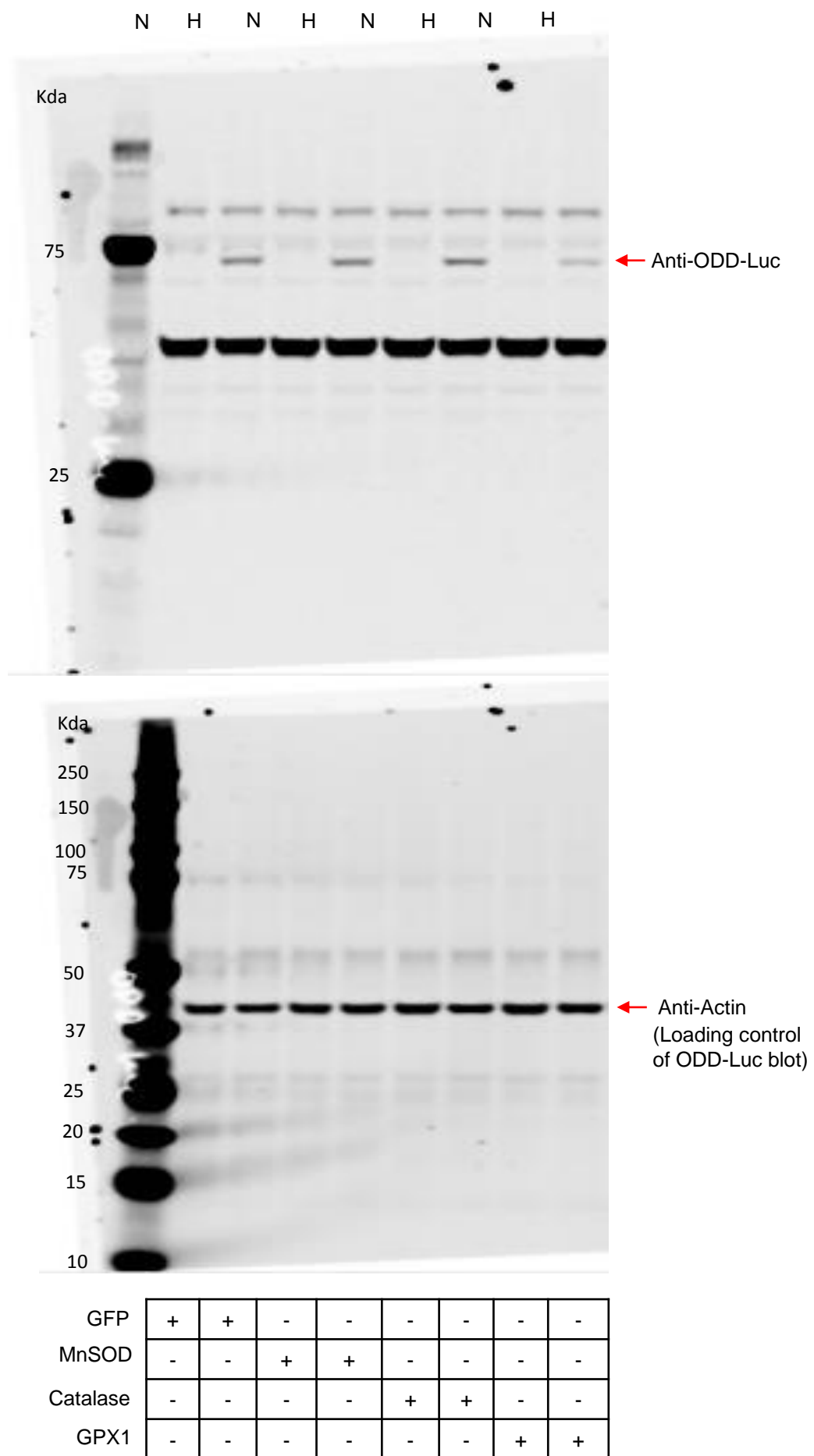

**Figure 3C**

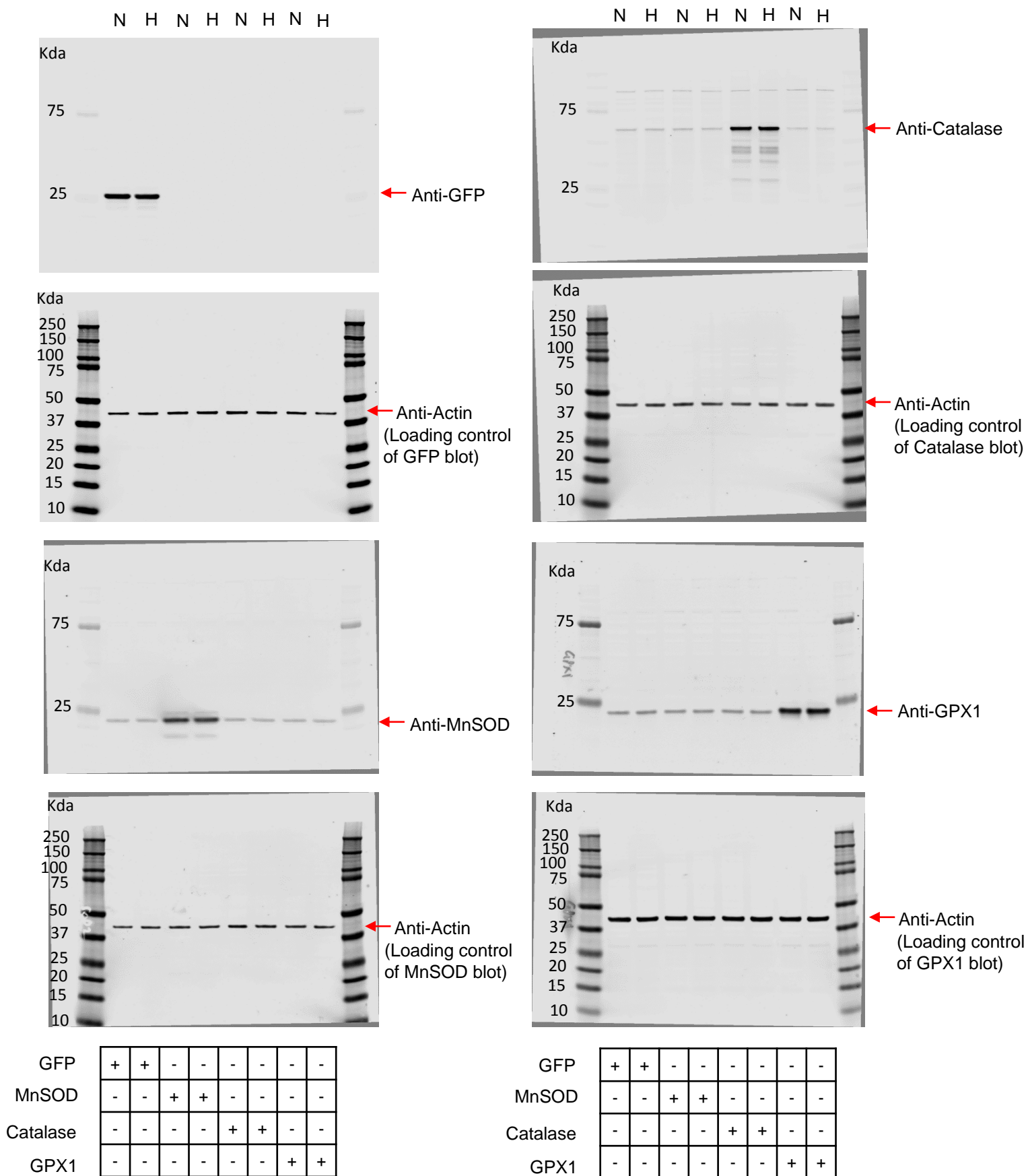

**Figure 3C**

Supplement: Figure 3—source data 1. [file elife-72873-fig3-data1.zip › Figure 3 - source data 1/Figure 3 - source data 1.pdf]

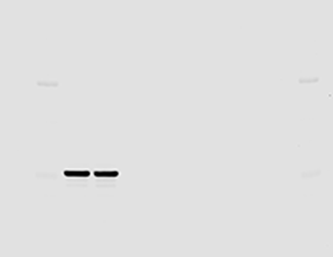

Supplement: Figure 3—source data 1. [file elife-72873-fig3-data1.zip › Figure 3 - source data 1/GFP blot.tif]

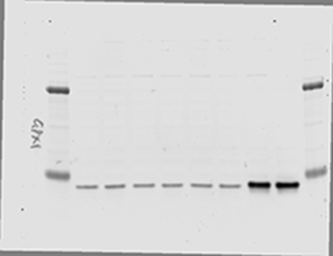

Supplement: Figure 3—source data 1. [file elife-72873-fig3-data1.zip › Figure 3 - source data 1/GPX1 blot.tif]

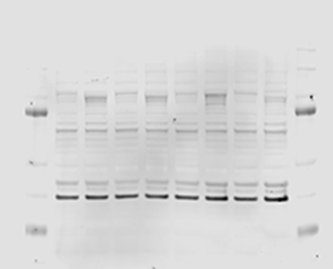

Supplement: Figure 3—source data 1. [file elife-72873-fig3-data1.zip › Figure 3 - source data 1/HIF1alpha blot.tif]

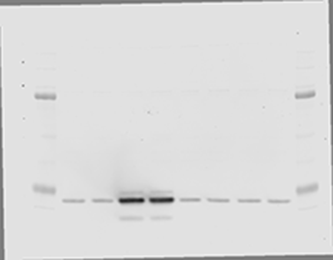

Supplement: Figure 3—source data 1. [file elife-72873-fig3-data1.zip › Figure 3 - source data 1/MnSOD blot.tif]

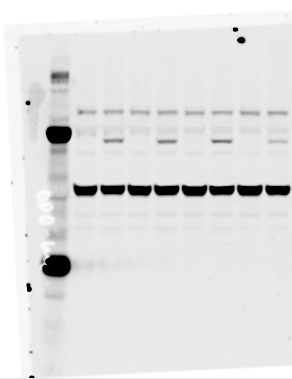

Supplement: Figure 3—source data 1. [file elife-72873-fig3-data1.zip › Figure 3 - source data 1/ODD-Luc blot.tif]

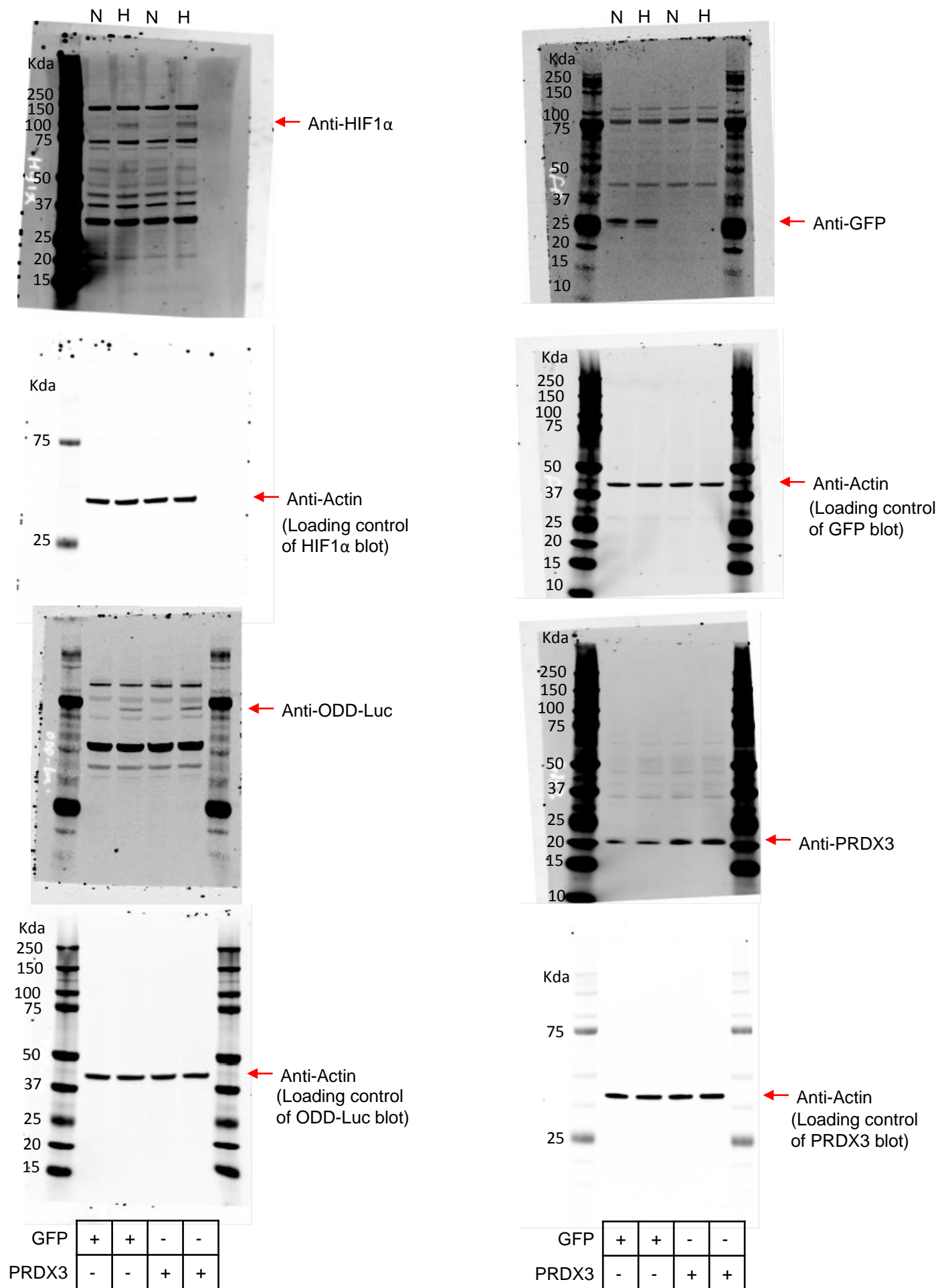

**Figure 3F**

Supplement: Figure 3—source data 2. [file elife-72873-fig3-data2.zip › Figure 3 - source data 2/Figure 3 - source data 2.pdf]

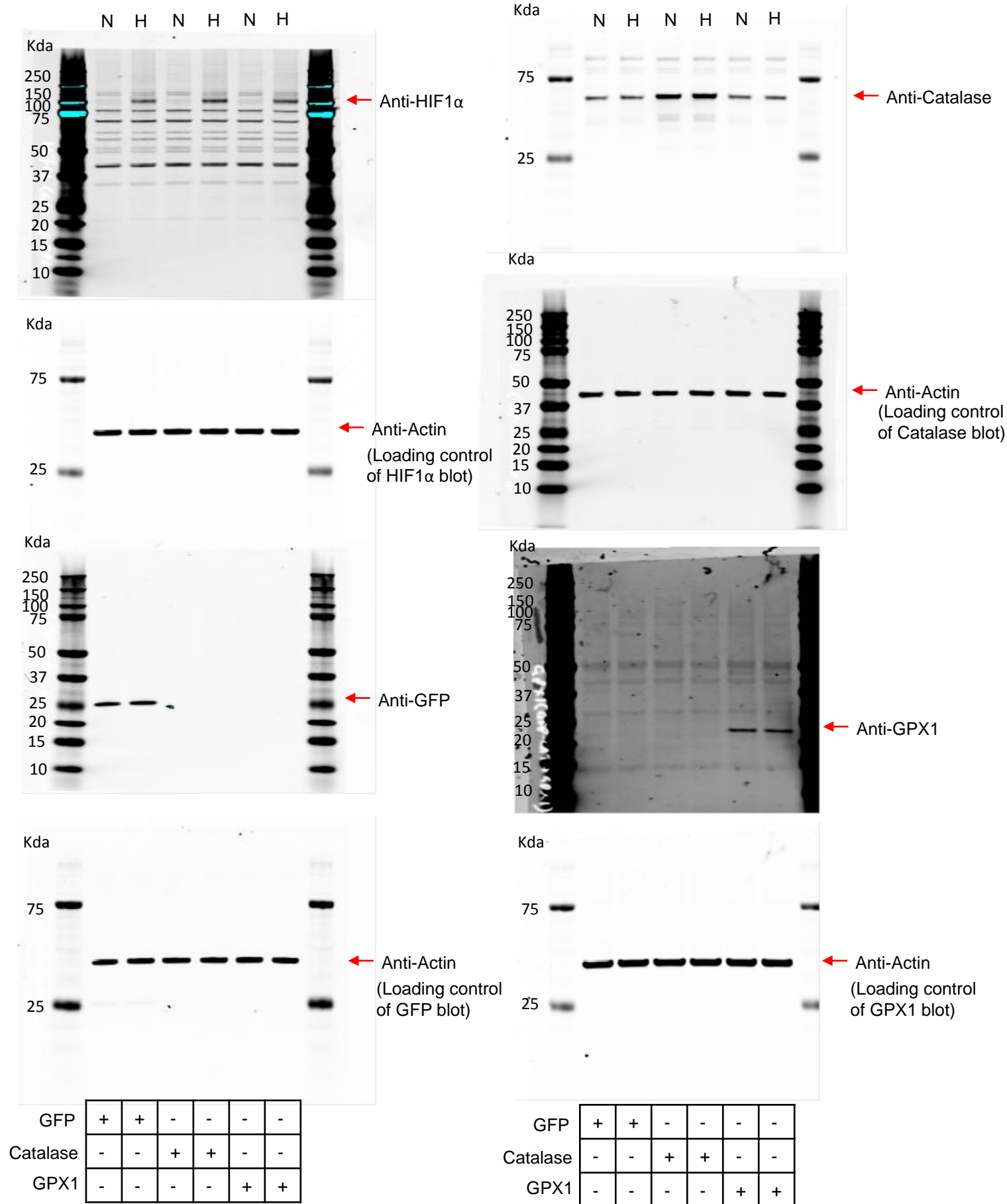

**Figure S6A**

Supplement: Figure 3—figure supplement 4—source data 1. [file elife-72873-fig3-figsupp4-data1.zip › Figure S6 - source data 1/Figure S6 - source data 1.pdf]

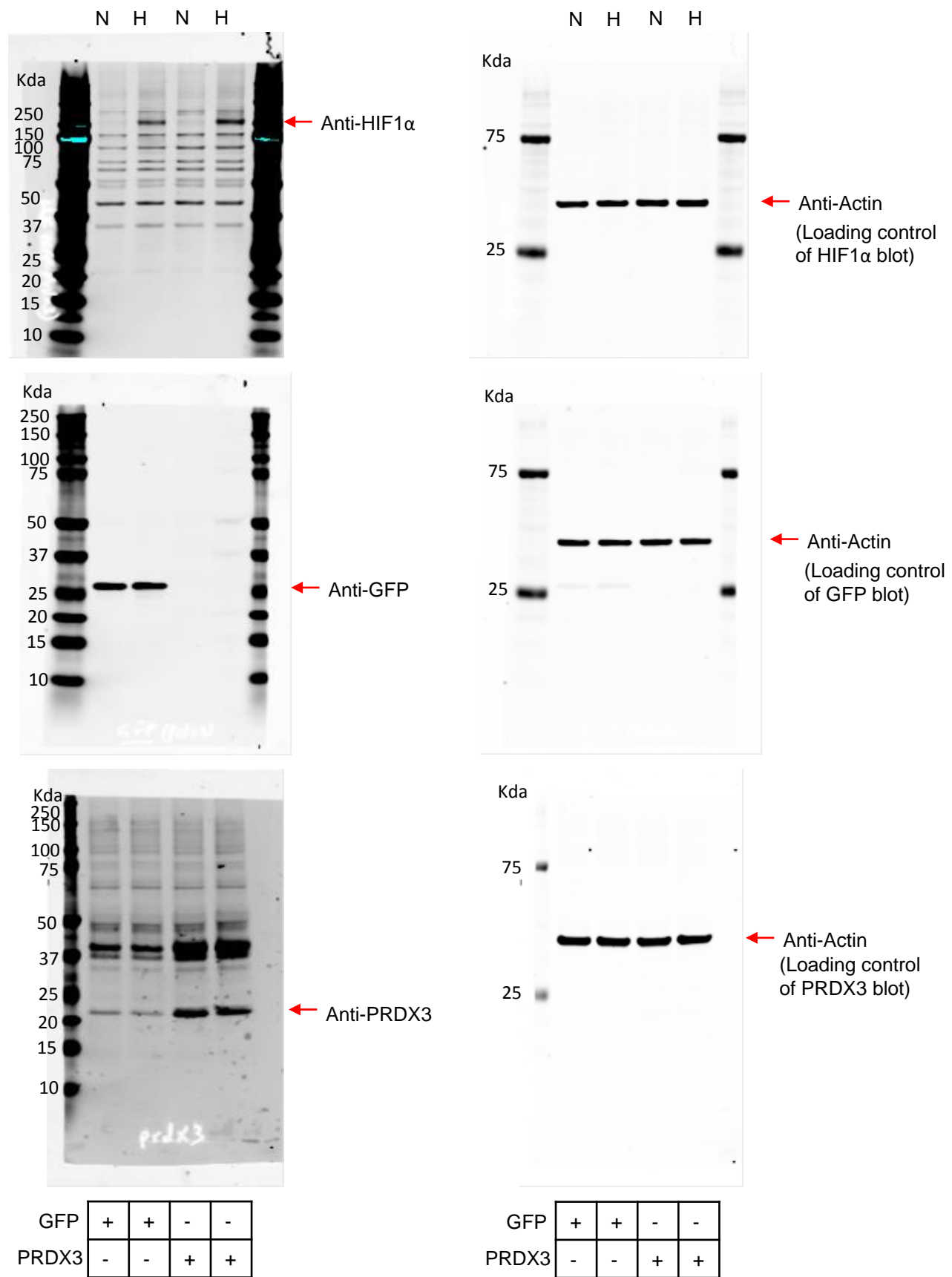

**Figure S6C**

Supplement: Figure 3—figure supplement 4—source data 2. [file elife-72873-fig3-figsupp4-data2.zip › Figure S6 - source data 2/Figure S6 - source data 2.pdf]

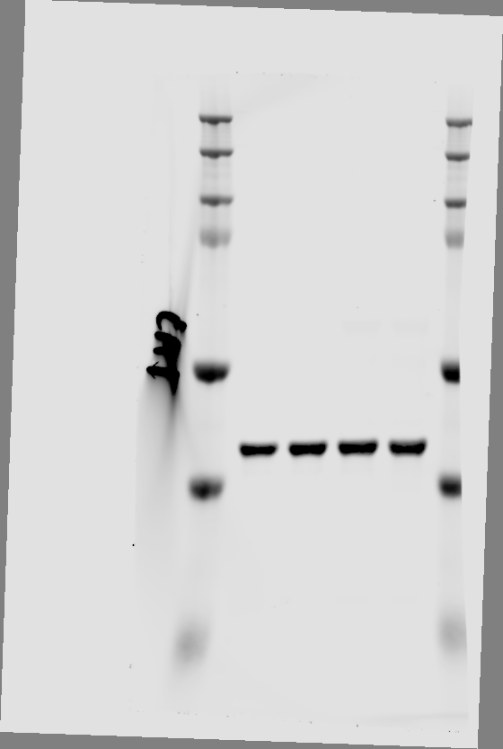

Supplement: Figure 3—figure supplement 5—source data 1. [file elife-72873-fig3-figsupp5-data1.zip › Figure S7 - source data 1/Actin control of catalase blot.tif]

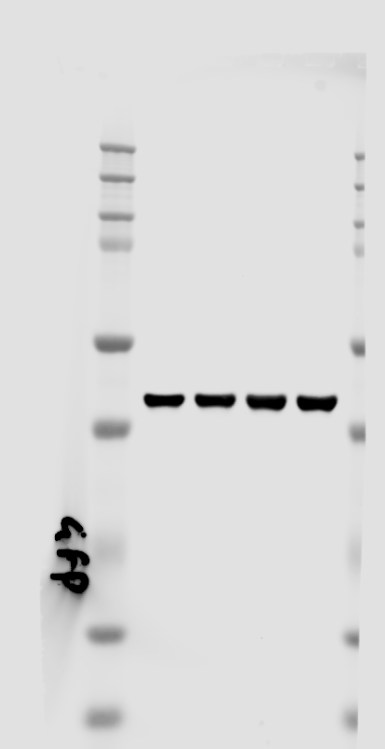

Supplement: Figure 3—figure supplement 5—source data 1. [file elife-72873-fig3-figsupp5-data1.zip › Figure S7 - source data 1/Actin control of GFP blot.tif]

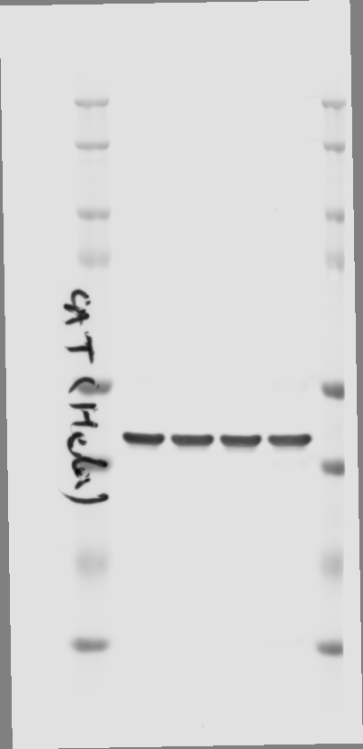

Supplement: Figure 3—figure supplement 5—source data 1. [file elife-72873-fig3-figsupp5-data1.zip › Figure S7 - source data 1/Actin control of HIF1alpha blot.tif]

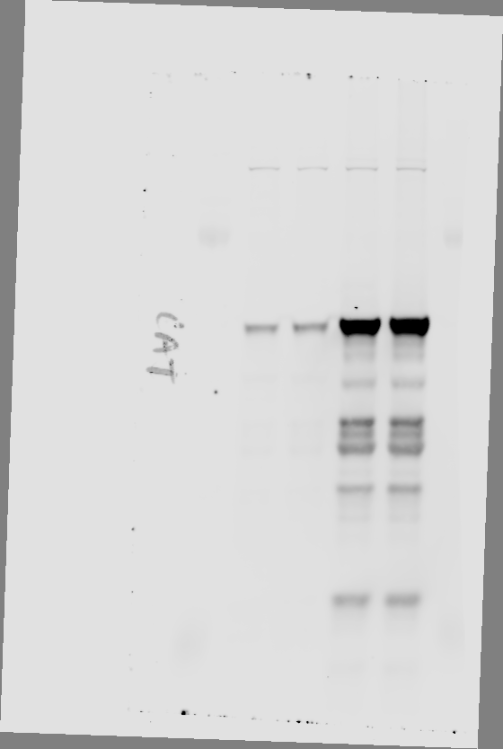

Supplement: Figure 3—figure supplement 5—source data 1. [file elife-72873-fig3-figsupp5-data1.zip › Figure S7 - source data 1/Catalase blot.tif]

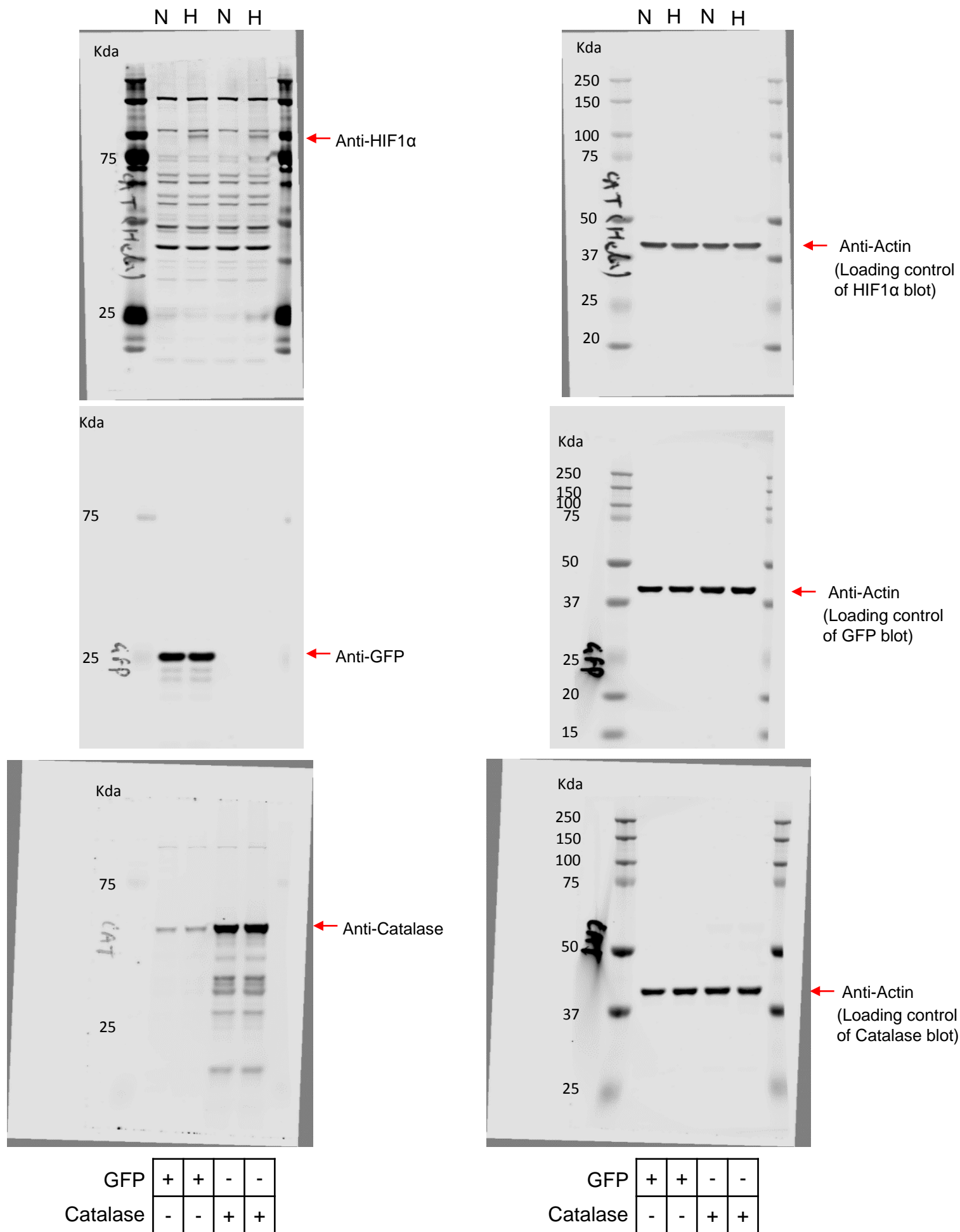

**Figure S7A**

Supplement: Figure 3—figure supplement 5—source data 1. [file elife-72873-fig3-figsupp5-data1.zip › Figure S7 - source data 1/Figure S7 - source data 1.pdf]

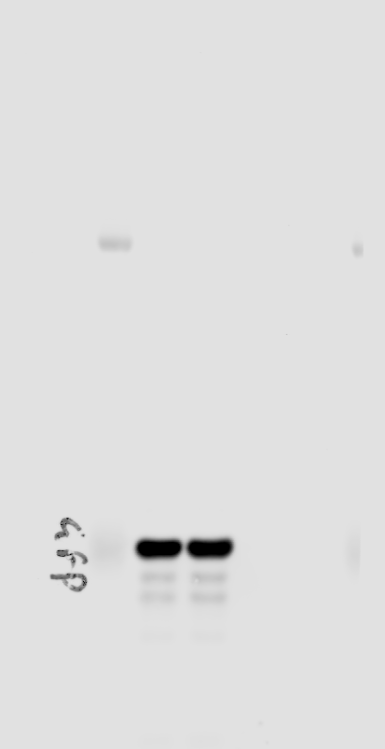

Supplement: Figure 3—figure supplement 5—source data 1. [file elife-72873-fig3-figsupp5-data1.zip › Figure S7 - source data 1/GFP blot.tif]

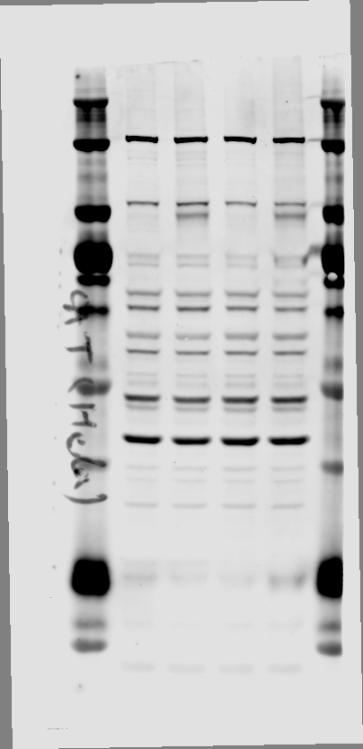

Supplement: Figure 3—figure supplement 5—source data 1. [file elife-72873-fig3-figsupp5-data1.zip › Figure S7 - source data 1/HIF1alpha blot.tif]

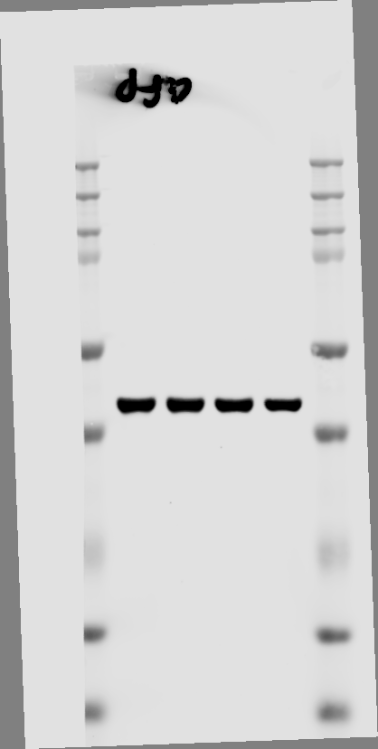

Supplement: Figure 3—figure supplement 5—source data 2. [file elife-72873-fig3-figsupp5-data2.zip › Figure S7 - source data 2/Actin control of GFP blot.tif]

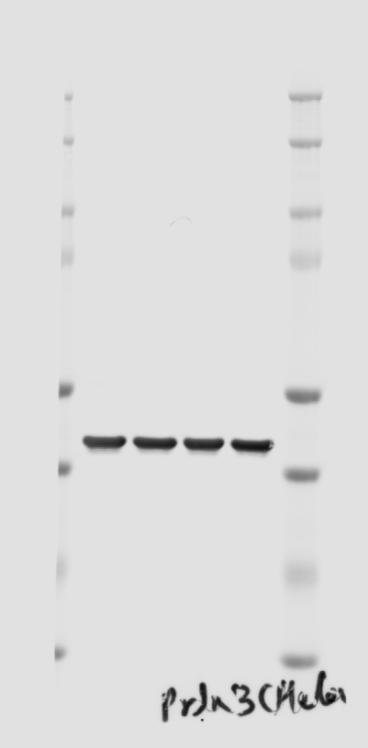

Supplement: Figure 3—figure supplement 5—source data 2. [file elife-72873-fig3-figsupp5-data2.zip › Figure S7 - source data 2/Actin control of HIF1alpha blot.tif]

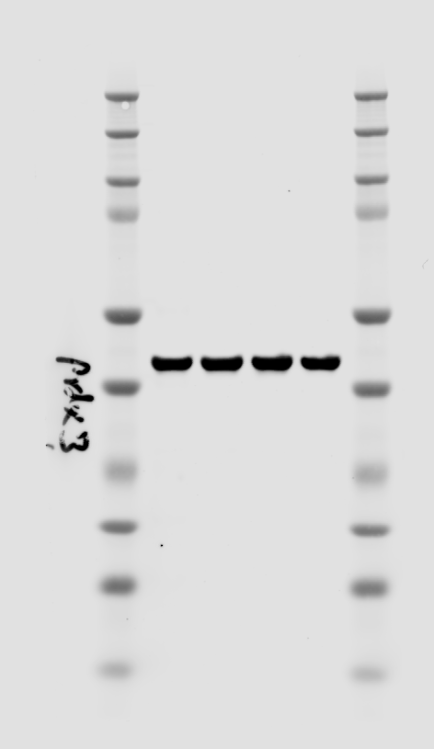

Supplement: Figure 3—figure supplement 5—source data 2. [file elife-72873-fig3-figsupp5-data2.zip › Figure S7 - source data 2/Actin control of PRDX3 blot.tif]

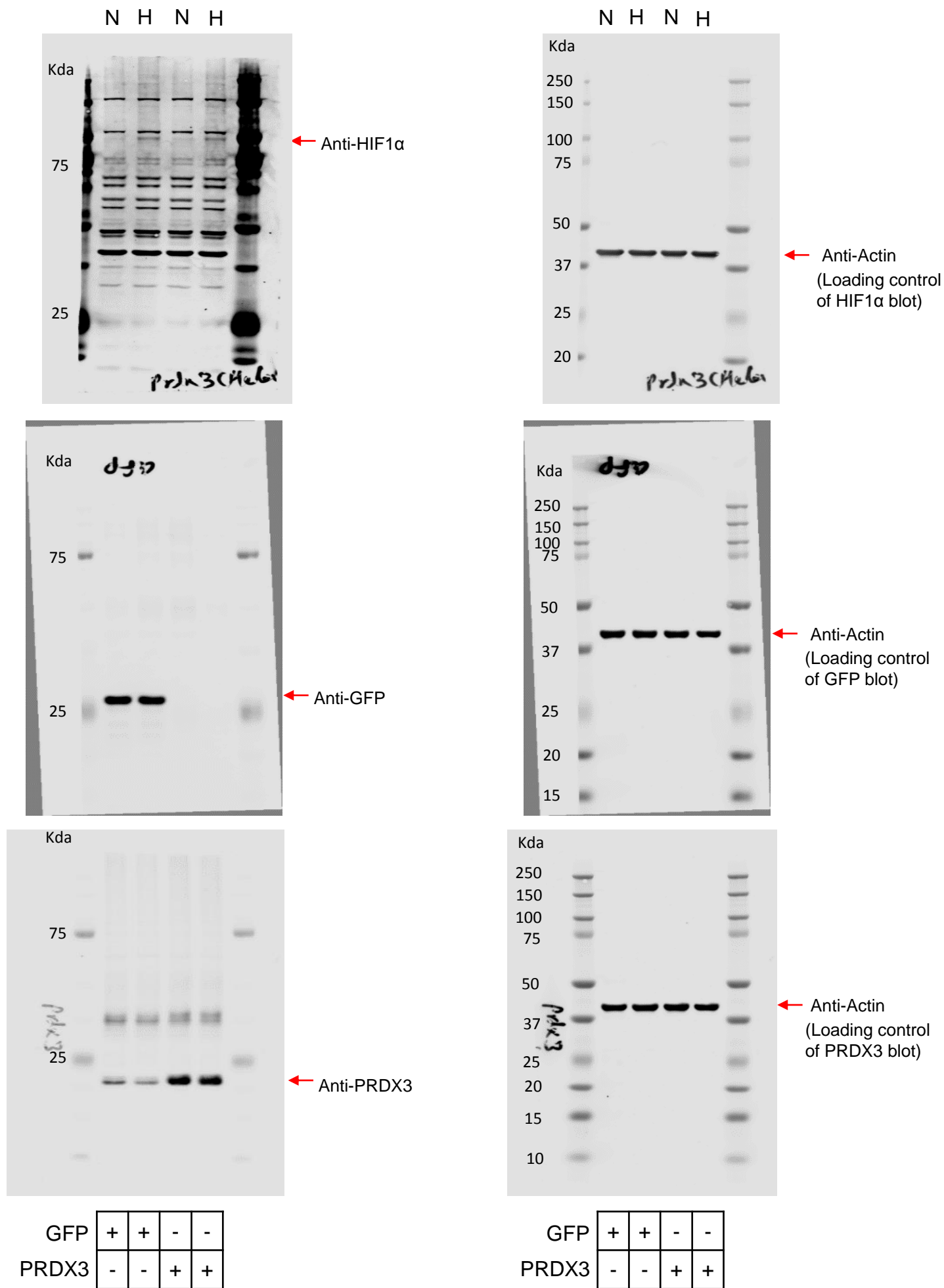

**Figure S7E**

Supplement: Figure 3—figure supplement 5—source data 2. [file elife-72873-fig3-figsupp5-data2.zip › Figure S7 - source data 2/Figure S7 - source data 2.pdf]

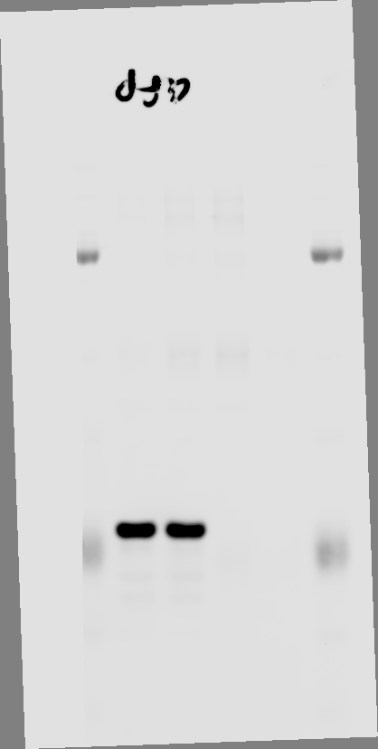

Supplement: Figure 3—figure supplement 5—source data 2. [file elife-72873-fig3-figsupp5-data2.zip › Figure S7 - source data 2/GFP blot.tif]

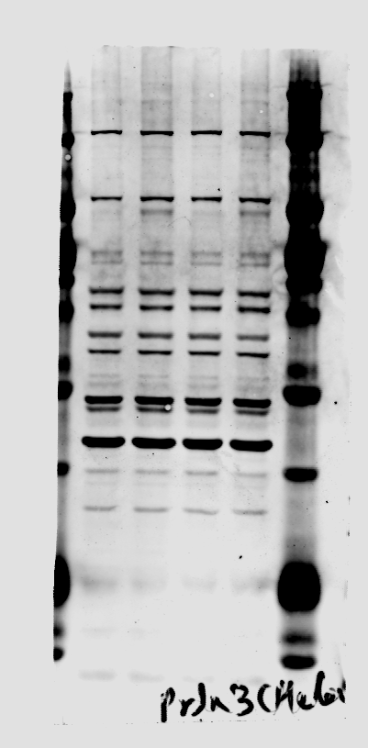

Supplement: Figure 3—figure supplement 5—source data 2. [file elife-72873-fig3-figsupp5-data2.zip › Figure S7 - source data 2/HIF1alpha blot.tif]

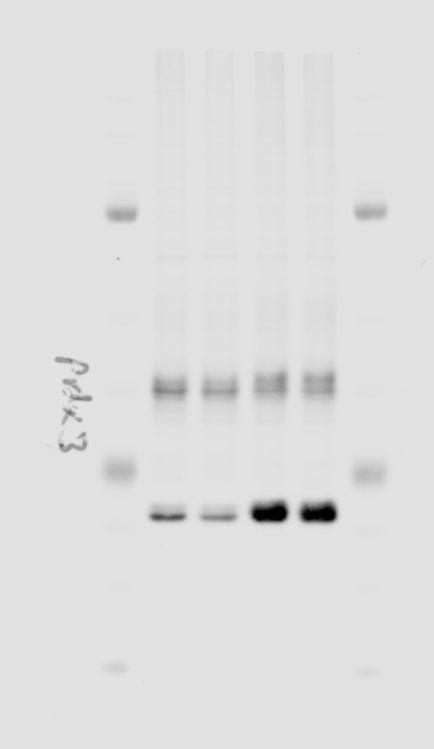

Supplement: Figure 3—figure supplement 5—source data 2. [file elife-72873-fig3-figsupp5-data2.zip › Figure S7 - source data 2/PRDX3 blot.tif]

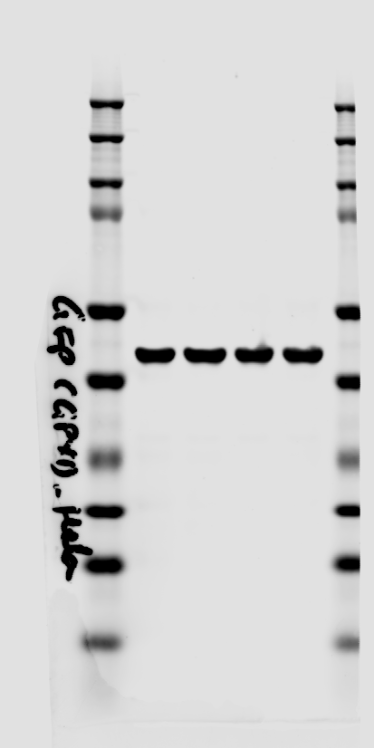

Supplement: Figure 3—figure supplement 5—source data 3. [file elife-72873-fig3-figsupp5-data3.zip › Figure S7 - source data 3/Actin control of GFP blot.tif]

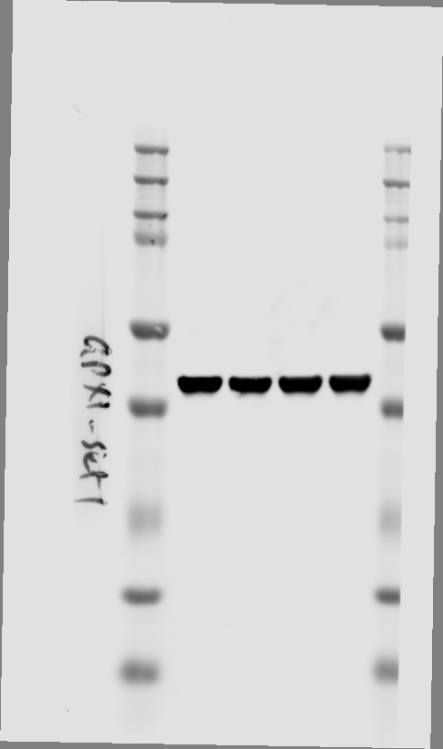

Supplement: Figure 3—figure supplement 5—source data 3. [file elife-72873-fig3-figsupp5-data3.zip › Figure S7 - source data 3/Actin control of GPX1 blot.tif]

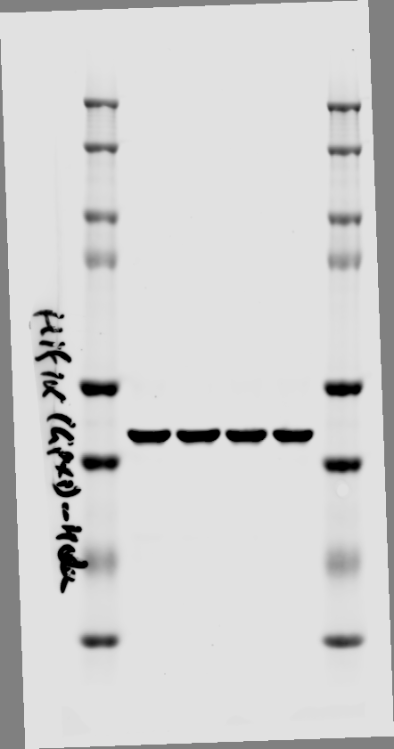

Supplement: Figure 3—figure supplement 5—source data 3. [file elife-72873-fig3-figsupp5-data3.zip › Figure S7 - source data 3/Actin control of HIF1alpha blot.tif]

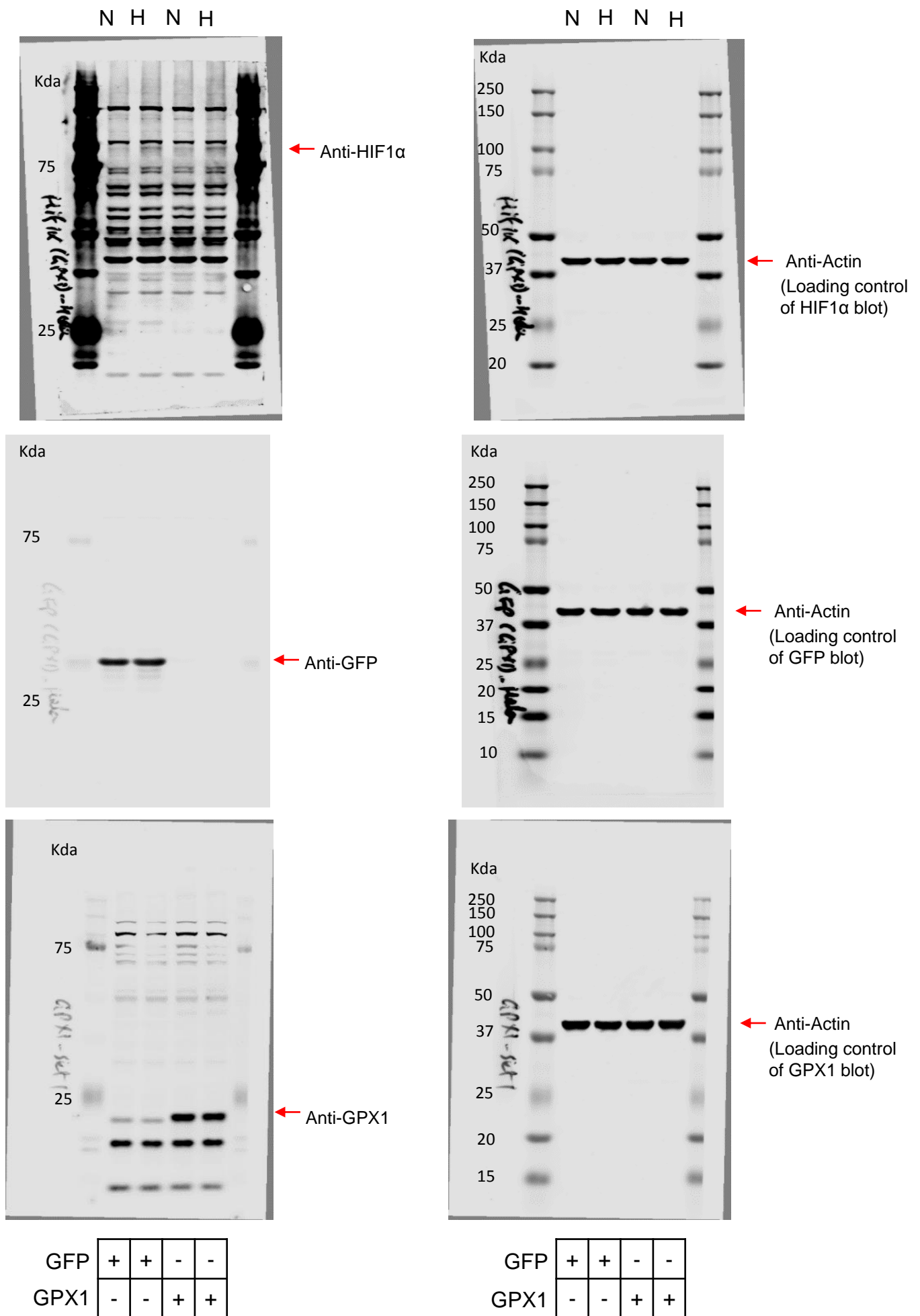

**Figure S7I**

Supplement: Figure 3—figure supplement 5—source data 3. [file elife-72873-fig3-figsupp5-data3.zip › Figure S7 - source data 3/Figure S7 - source data 3.pdf]

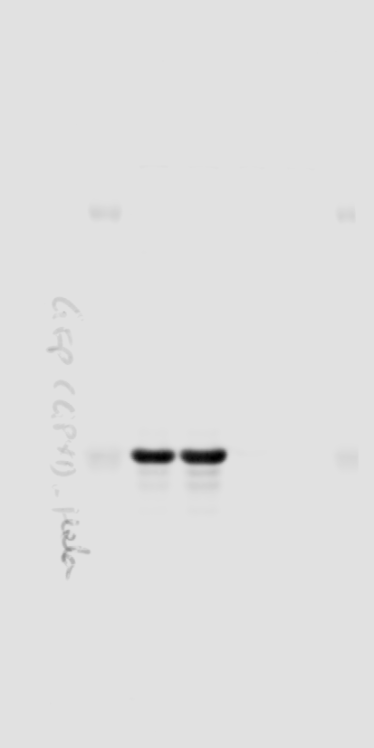

Supplement: Figure 3—figure supplement 5—source data 3. [file elife-72873-fig3-figsupp5-data3.zip › Figure S7 - source data 3/GFP blot.tif]

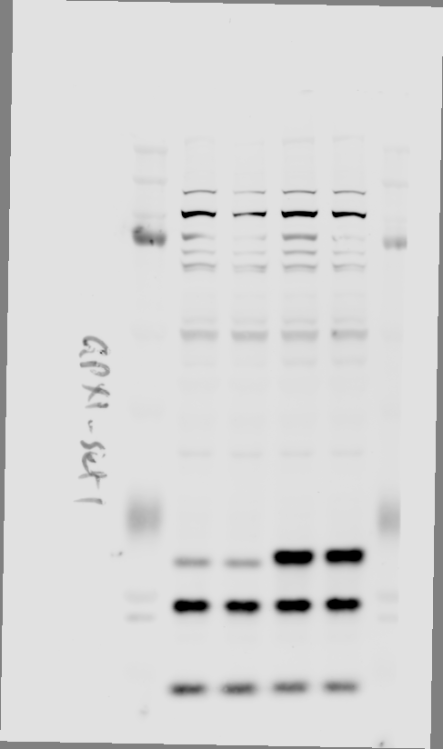

Supplement: Figure 3—figure supplement 5—source data 3. [file elife-72873-fig3-figsupp5-data3.zip › Figure S7 - source data 3/GPX1 blot.tif]

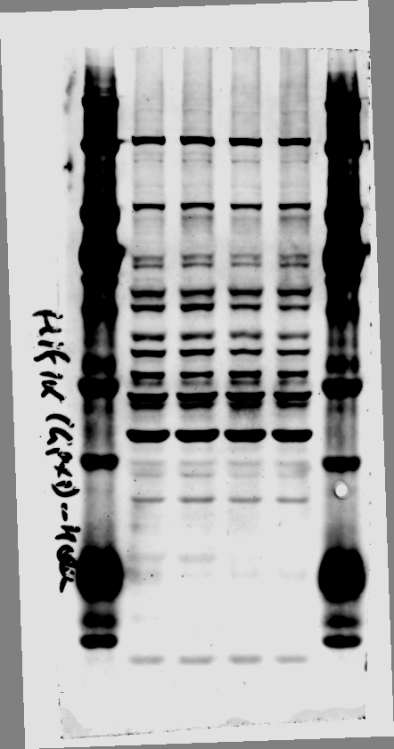

Supplement: Figure 3—figure supplement 5—source data 3. [file elife-72873-fig3-figsupp5-data3.zip › Figure S7 - source data 3/HIF1alpha blot.tif]

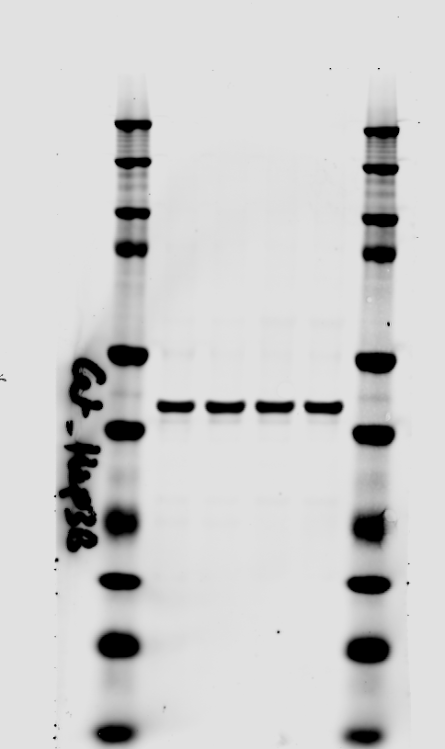

Supplement: Figure 3—figure supplement 6—source data 1. [file elife-72873-fig3-figsupp6-data1.zip › Figure S8 - source data 1/Actin control of catalase blot.tif]

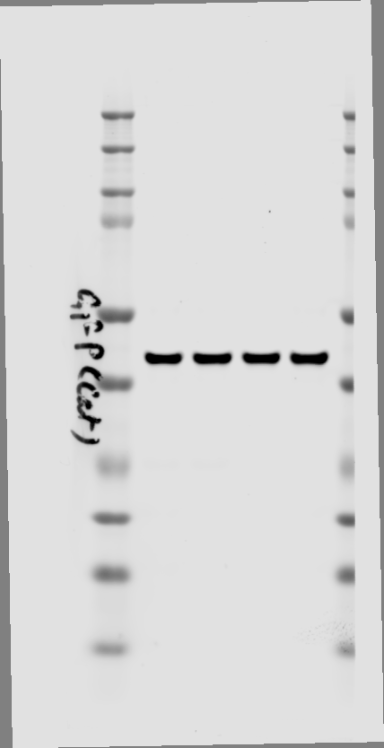

Supplement: Figure 3—figure supplement 6—source data 1. [file elife-72873-fig3-figsupp6-data1.zip › Figure S8 - source data 1/Actin control of GFP blot.tif]

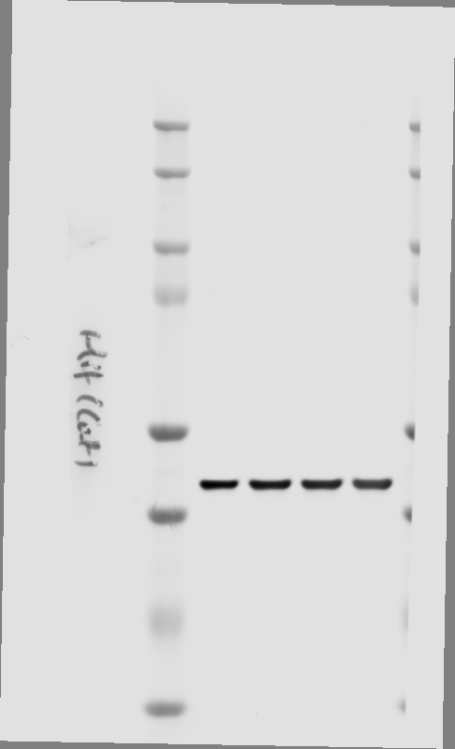

Supplement: Figure 3—figure supplement 6—source data 1. [file elife-72873-fig3-figsupp6-data1.zip › Figure S8 - source data 1/Actin control of HIF1alpha blot.tif]

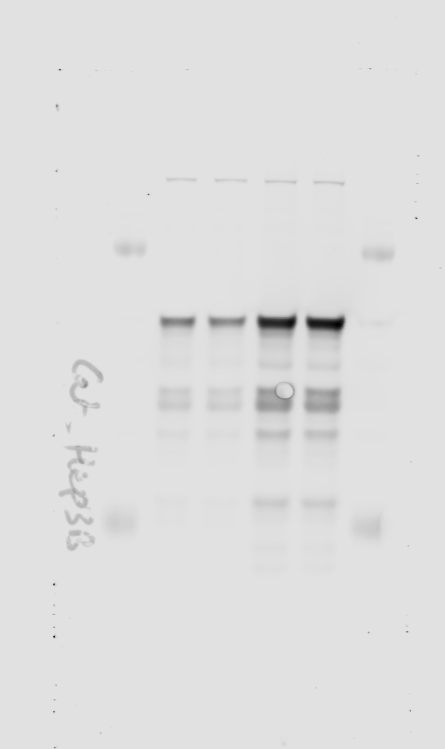

Supplement: Figure 3—figure supplement 6—source data 1. [file elife-72873-fig3-figsupp6-data1.zip › Figure S8 - source data 1/Catalase blot.tif]

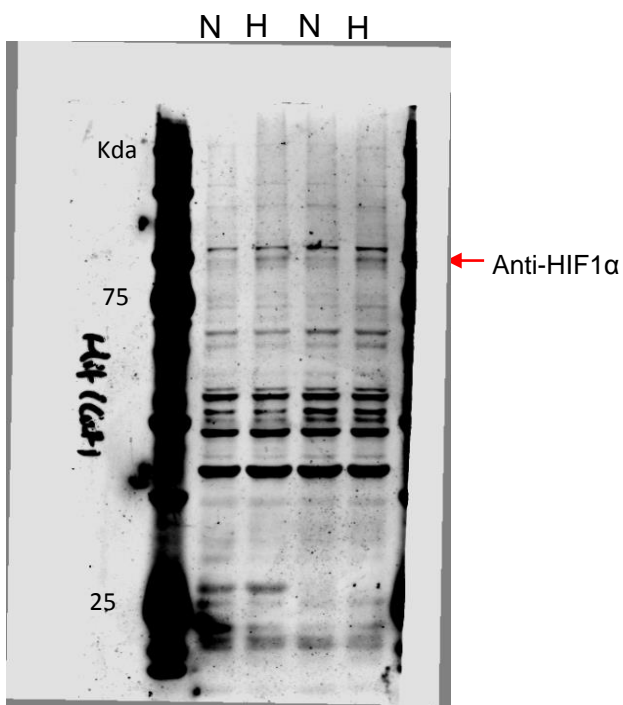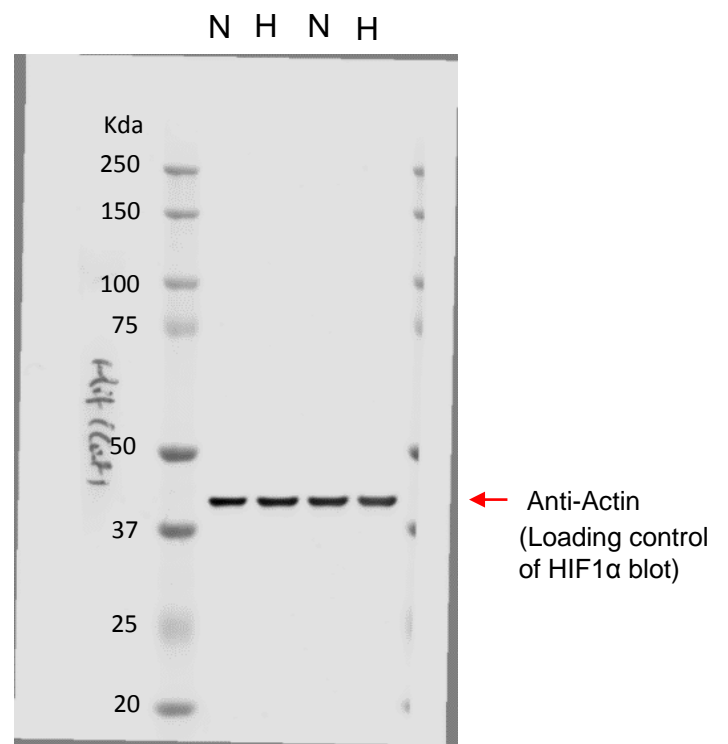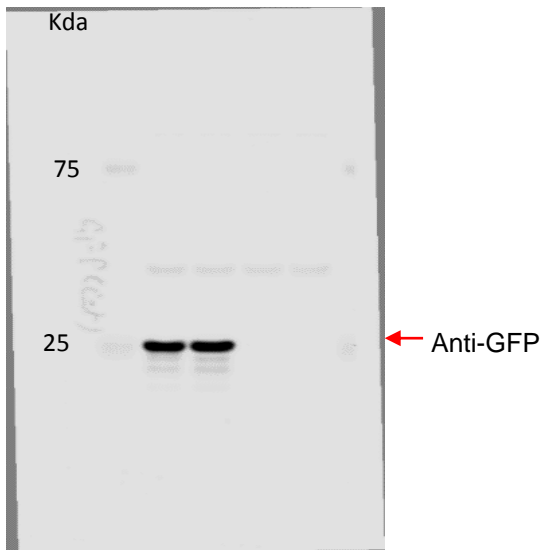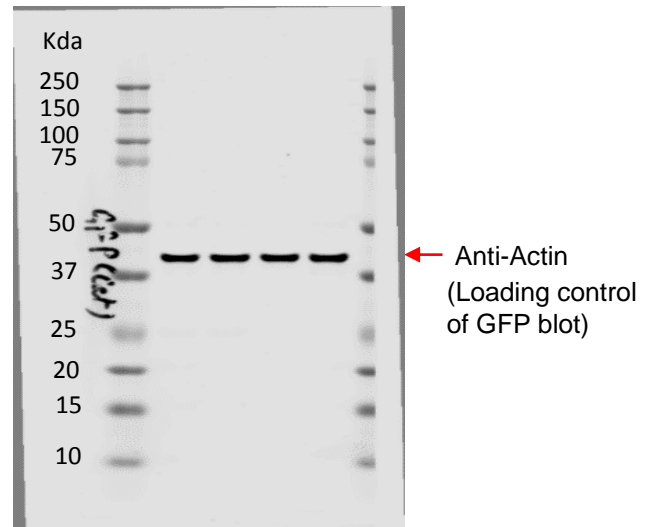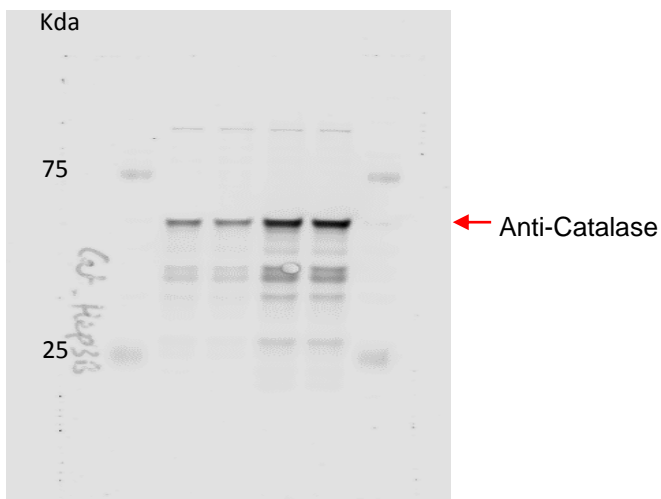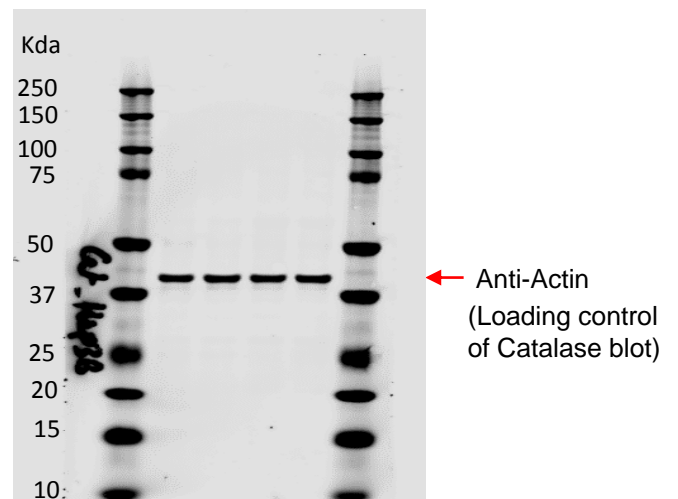

|          |   |   |   |   |
|----------|---|---|---|---|
| GFP      | + | + | - | - |
| Catalase | - | - | + | + |

|          |   |   |   |   |
|----------|---|---|---|---|
| GFP      | + | + | - | - |
| Catalase | - | - | + | + |

Figure S8A

Supplement: Figure 3—figure supplement 6—source data 1. [file elife-72873-fig3-figsupp6-data1.zip › Figure S8 - source data 1/Figure S8 - source data 1.pdf]

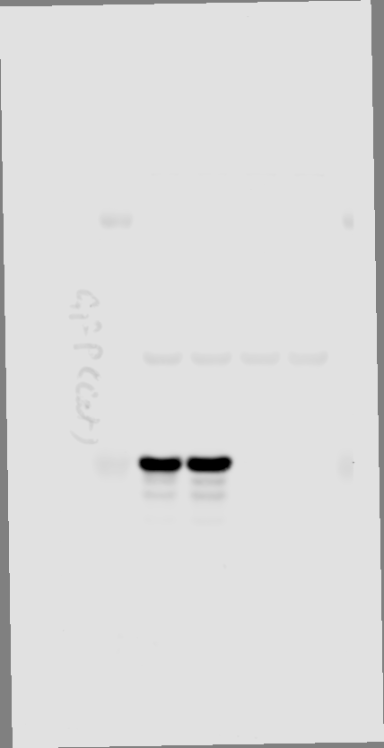

Supplement: Figure 3—figure supplement 6—source data 1. [file elife-72873-fig3-figsupp6-data1.zip › Figure S8 - source data 1/GFP blot.tif]

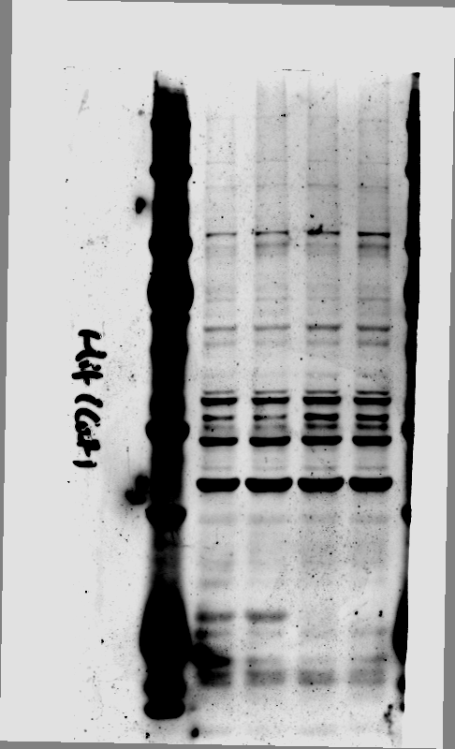

Supplement: Figure 3—figure supplement 6—source data 1. [file elife-72873-fig3-figsupp6-data1.zip › Figure S8 - source data 1/HIF1alpha blot.tif]

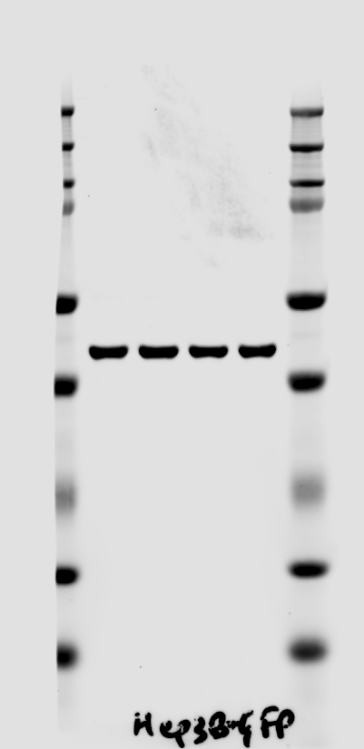

Supplement: Figure 3—figure supplement 6—source data 2. [file elife-72873-fig3-figsupp6-data2.zip › Figure S8 - source data 2/Actin control of GFP blot.tif]

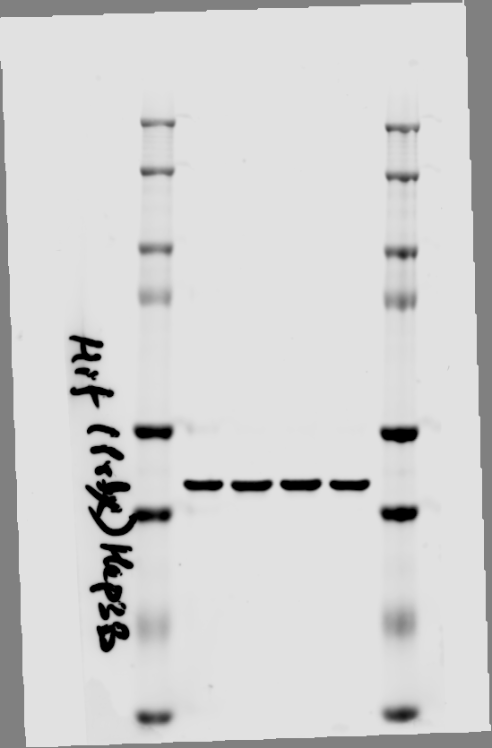

Supplement: Figure 3—figure supplement 6—source data 2. [file elife-72873-fig3-figsupp6-data2.zip › Figure S8 - source data 2/Actin control of HIF1alpha blot.tif]

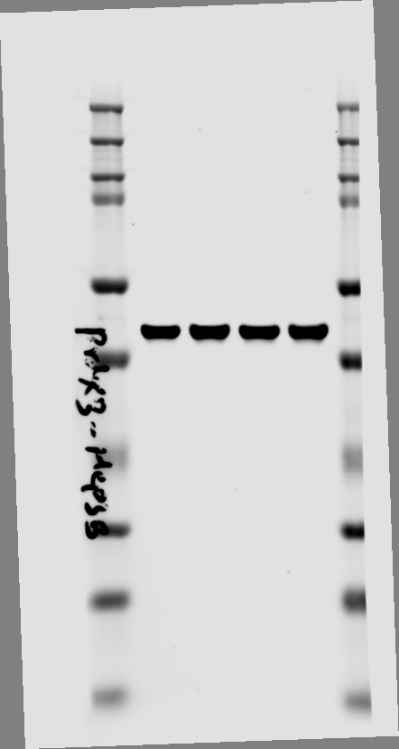

Supplement: Figure 3—figure supplement 6—source data 2. [file elife-72873-fig3-figsupp6-data2.zip › Figure S8 - source data 2/Actin control of PRDX3 blot.tif]

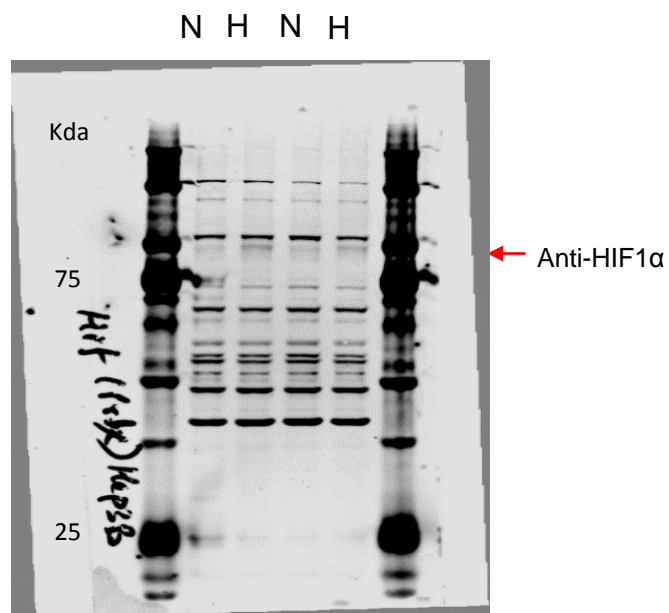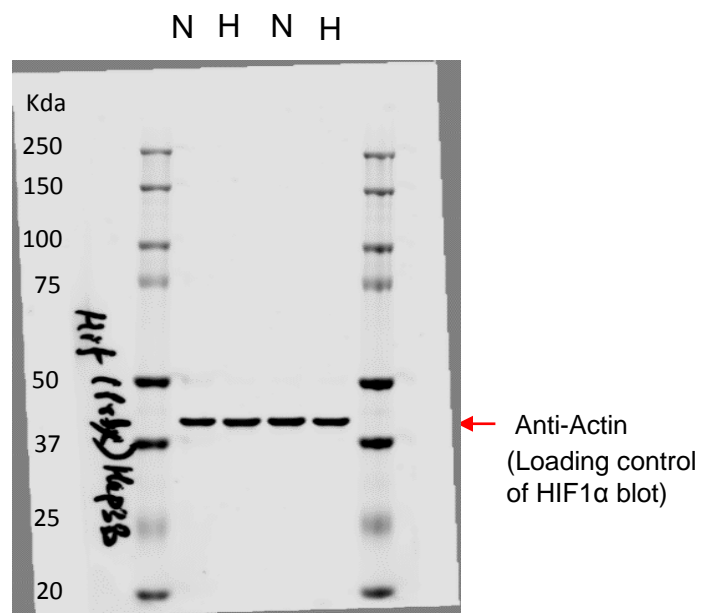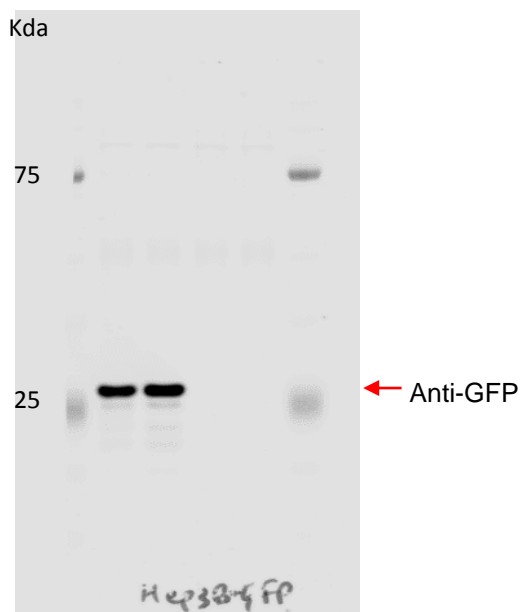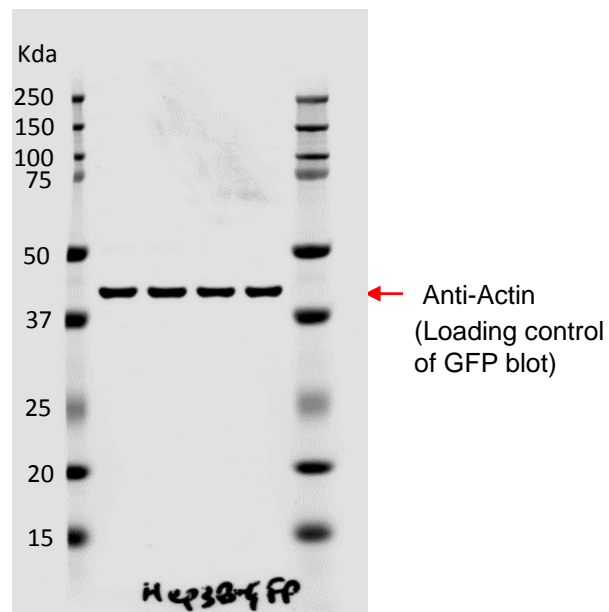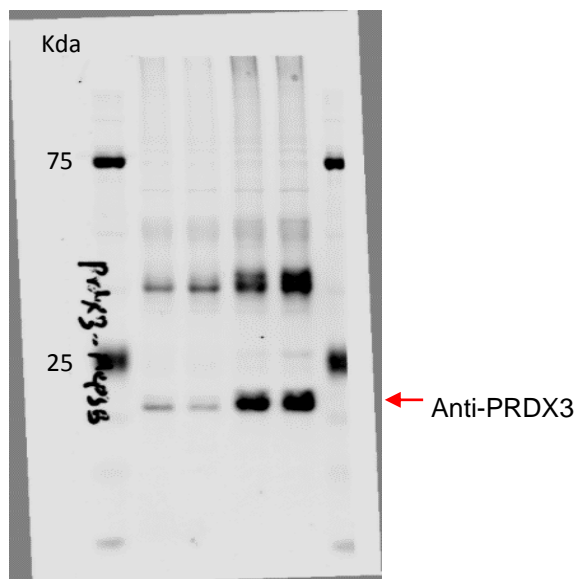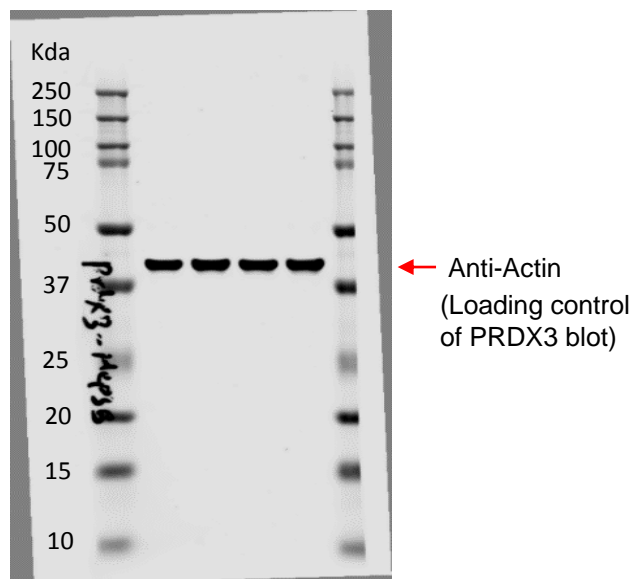

|       |   |   |   |   |
|-------|---|---|---|---|
| GFP   | + | + | - | - |
| PRDX3 | - | - | + | + |

|       |   |   |   |   |
|-------|---|---|---|---|
| GFP   | + | + | - | - |
| PRDX3 | - | - | + | + |

Figure S8E

Supplement: Figure 3—figure supplement 6—source data 2. [file elife-72873-fig3-figsupp6-data2.zip › Figure S8 - source data 2/Figure S8 - source data 2.pdf]

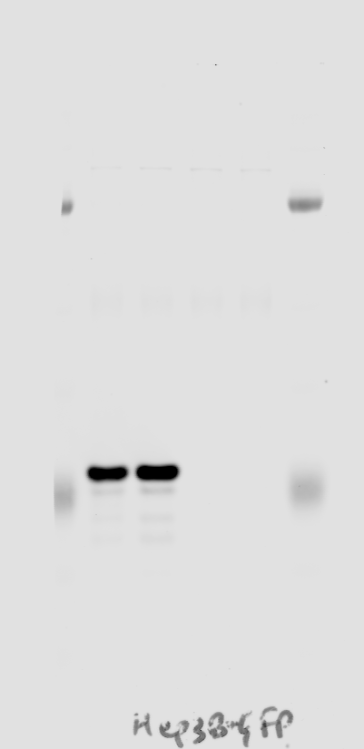

Supplement: Figure 3—figure supplement 6—source data 2. [file elife-72873-fig3-figsupp6-data2.zip › Figure S8 - source data 2/GFP blot.tif]

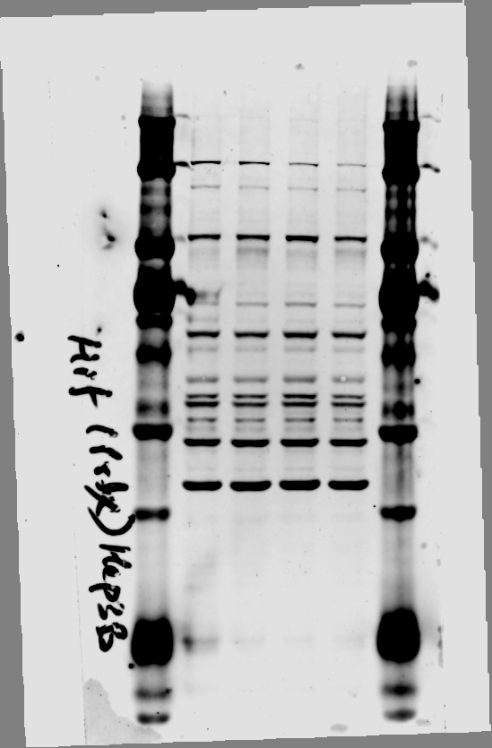

Supplement: Figure 3—figure supplement 6—source data 2. [file elife-72873-fig3-figsupp6-data2.zip › Figure S8 - source data 2/HIF1alpha blot.tif]

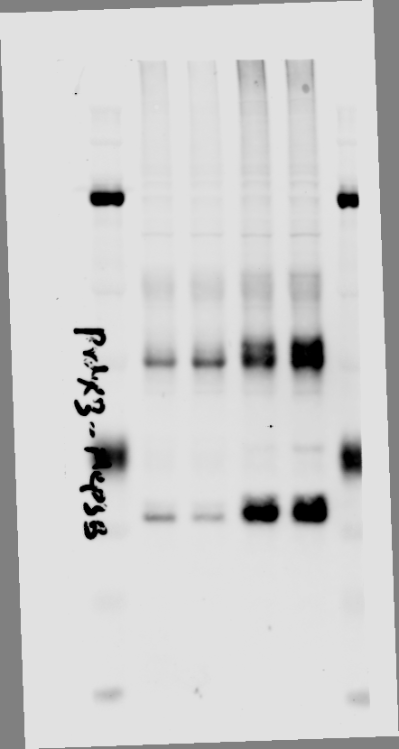

Supplement: Figure 3—figure supplement 6—source data 2. [file elife-72873-fig3-figsupp6-data2.zip › Figure S8 - source data 2/PRDX3 blot.tif]

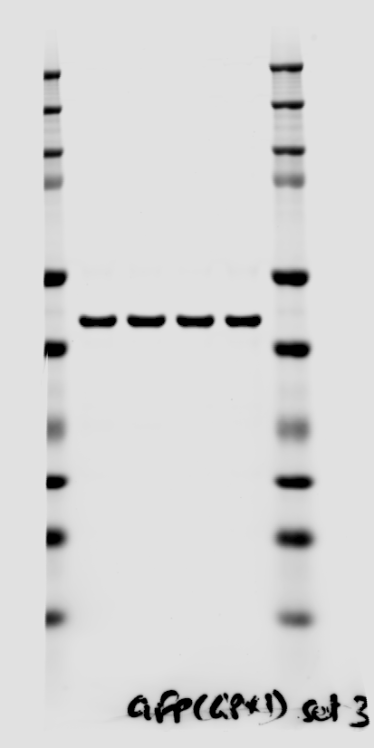

Supplement: Figure 3—figure supplement 6—source data 3. [file elife-72873-fig3-figsupp6-data3.zip › Figure S8 - source data 3/Actin control of GFP blot.tif]

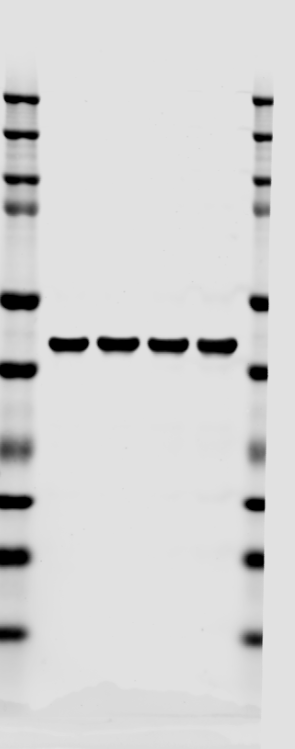

Supplement: Figure 3—figure supplement 6—source data 3. [file elife-72873-fig3-figsupp6-data3.zip › Figure S8 - source data 3/Actin control of GPX1 blot.tif]

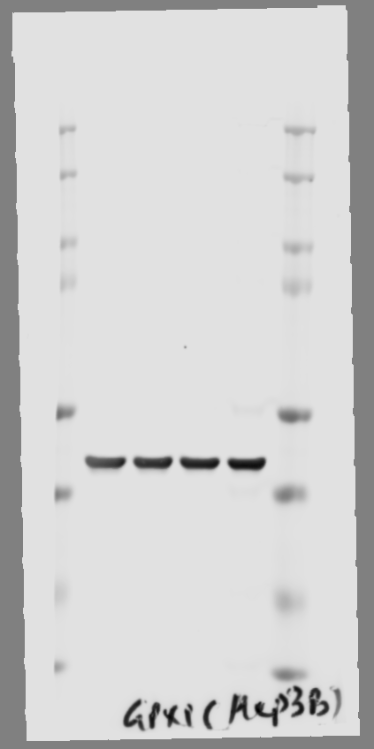

Supplement: Figure 3—figure supplement 6—source data 3. [file elife-72873-fig3-figsupp6-data3.zip › Figure S8 - source data 3/Actin control of HIF1alpha blot.tif]

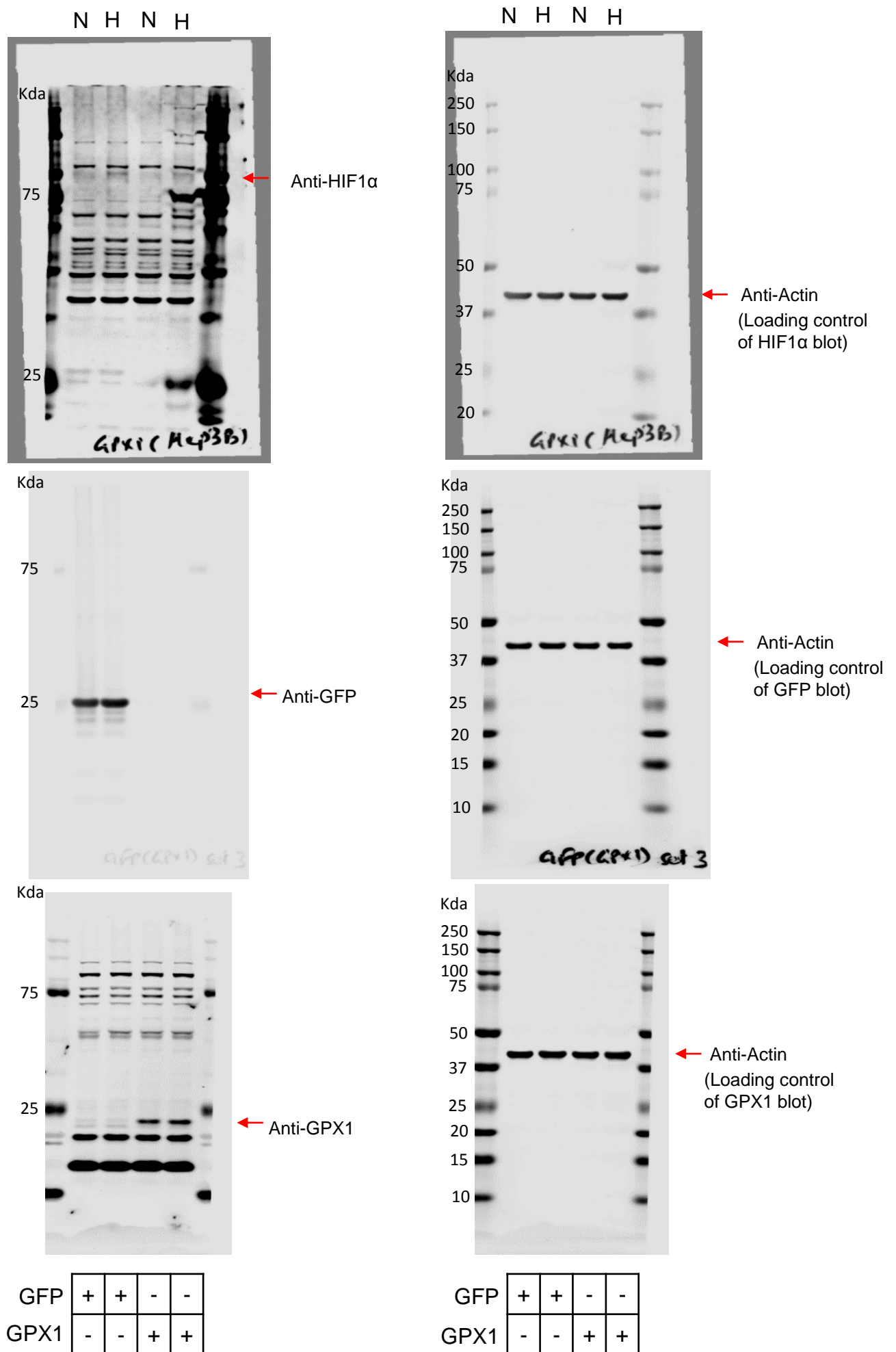

**Figure S8I**

Supplement: Figure 3—figure supplement 6—source data 3. [file elife-72873-fig3-figsupp6-data3.zip › Figure S8 - source data 3/Figure S8 - source data 3.pdf]

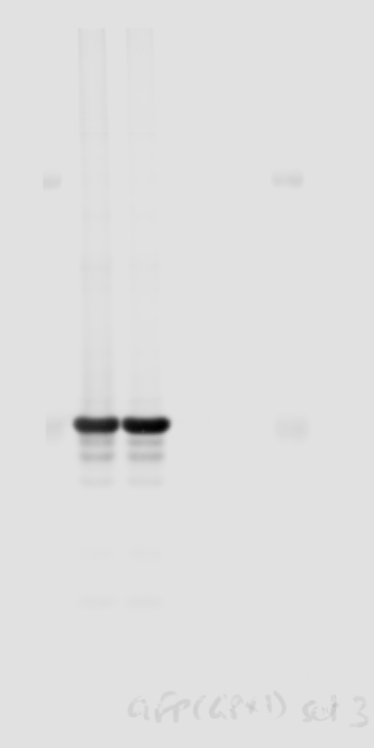

Supplement: Figure 3—figure supplement 6—source data 3. [file elife-72873-fig3-figsupp6-data3.zip › Figure S8 - source data 3/GFP blot.tif]

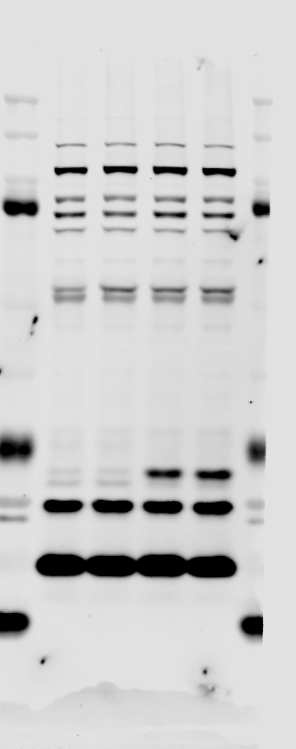

Supplement: Figure 3—figure supplement 6—source data 3. [file elife-72873-fig3-figsupp6-data3.zip › Figure S8 - source data 3/GPX1 blot.tif]

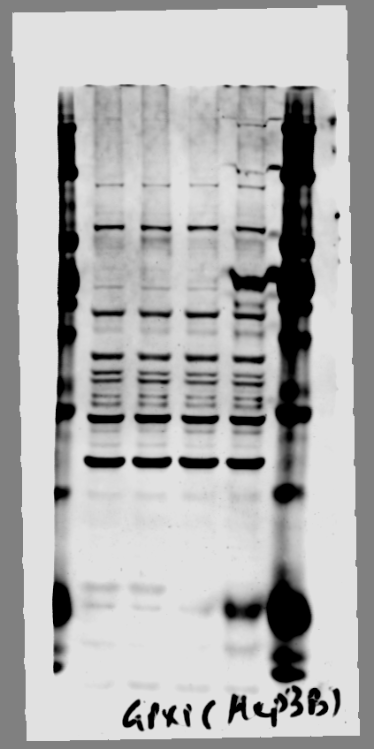

Supplement: Figure 3—figure supplement 6—source data 3. [file elife-72873-fig3-figsupp6-data3.zip › Figure S8 - source data 3/HIF1alpha blot.tif]

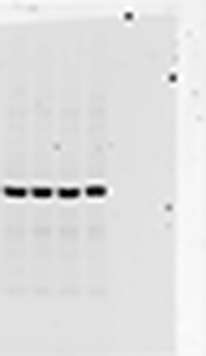

Supplement: Figure 5—source data 1. [file elife-72873-fig5-data1.zip › Figure 5 - source data 1/Actin control of GFP blot.tif]

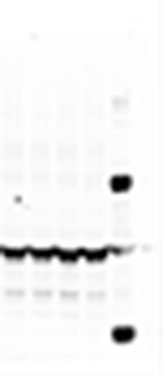

Supplement: Figure 5—source data 1. [file elife-72873-fig5-data1.zip › Figure 5 - source data 1/Actin control of GPX4 blot.tif]

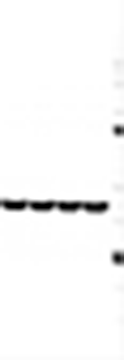

Supplement: Figure 5—source data 1. [file elife-72873-fig5-data1.zip › Figure 5 - source data 1/Actin control of HIF1alpha blot.tif]

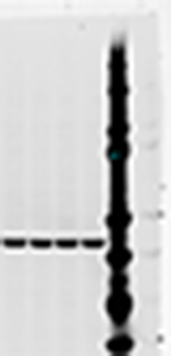

Supplement: Figure 5—source data 1. [file elife-72873-fig5-data1.zip › Figure 5 - source data 1/Actin control of ODD-Luc blot.tif]

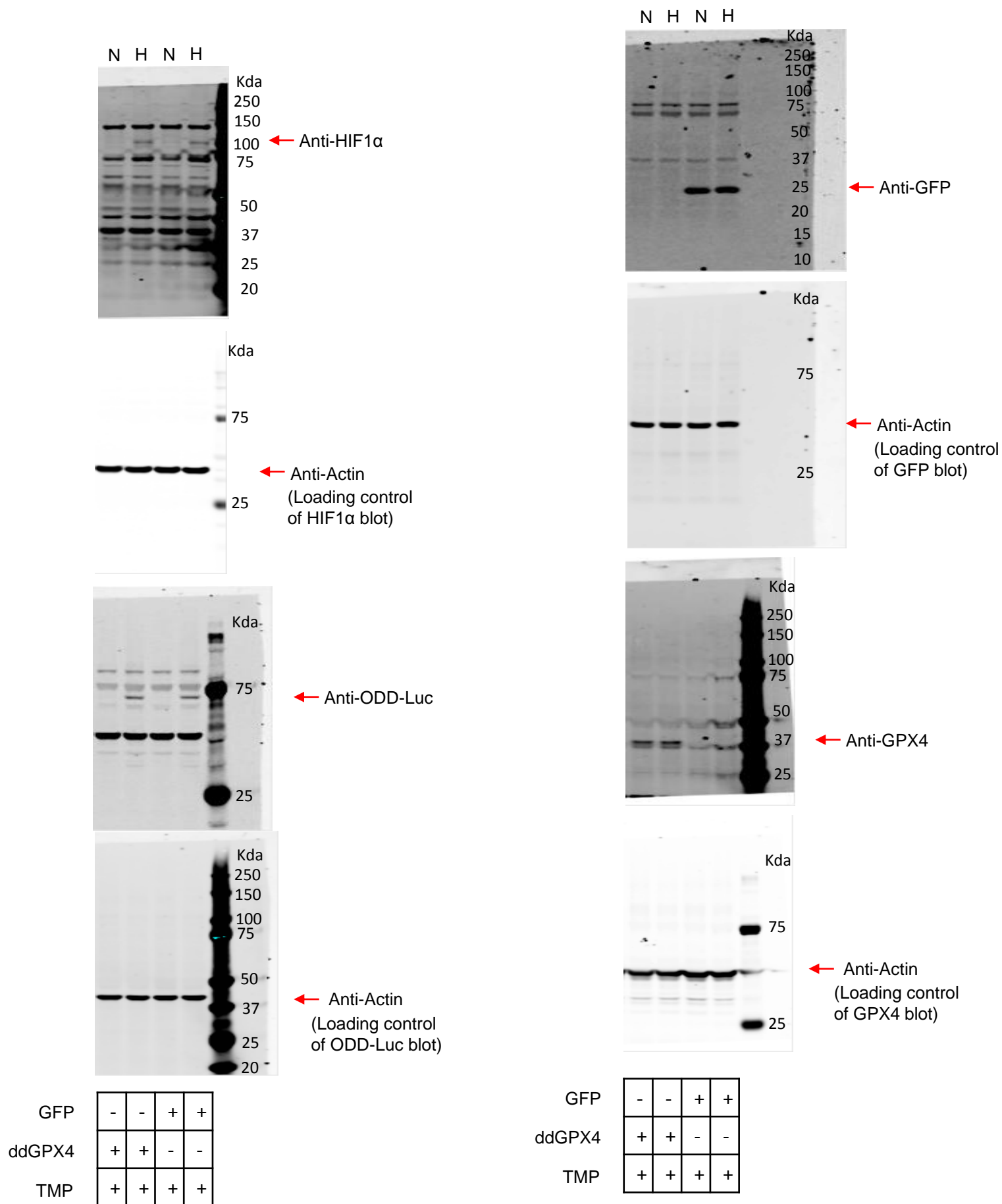

**Figure 6D**

Supplement: Figure 5—source data 1. [file elife-72873-fig5-data1.zip › Figure 5 - source data 1/Figure 5 - source data 1.pdf]

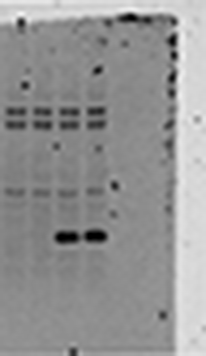

Supplement: Figure 5—source data 1. [file elife-72873-fig5-data1.zip › Figure 5 - source data 1/GFP blot.tif]

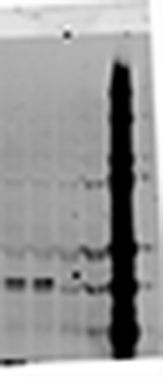

Supplement: Figure 5—source data 1. [file elife-72873-fig5-data1.zip › Figure 5 - source data 1/GPX4 blot.tif]

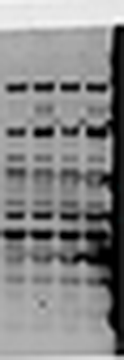

Supplement: Figure 5—source data 1. [file elife-72873-fig5-data1.zip › Figure 5 - source data 1/HIF1alpha blot.tif]

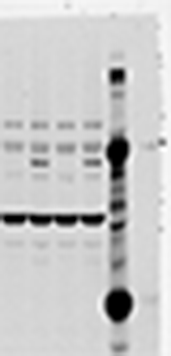

Supplement: Figure 5—source data 1. [file elife-72873-fig5-data1.zip › Figure 5 - source data 1/ODD-luc blot.tif]
