# Supplementary material for: HIF1α stabilization in hypoxia is not oxidant-initiated
Source: eLife. 2021 Oct 1;10:e72873. doi: 10.7554/eLife.72873 (PMC8530508; doi:10.7554/eLife.72873)
Supplement: Figure 2—figure supplement 1—source data 1. [file elife-72873-fig2-figsupp1-data1.zip › Figure S1 - source data 1/Figure S1 - source data 1.pdf]

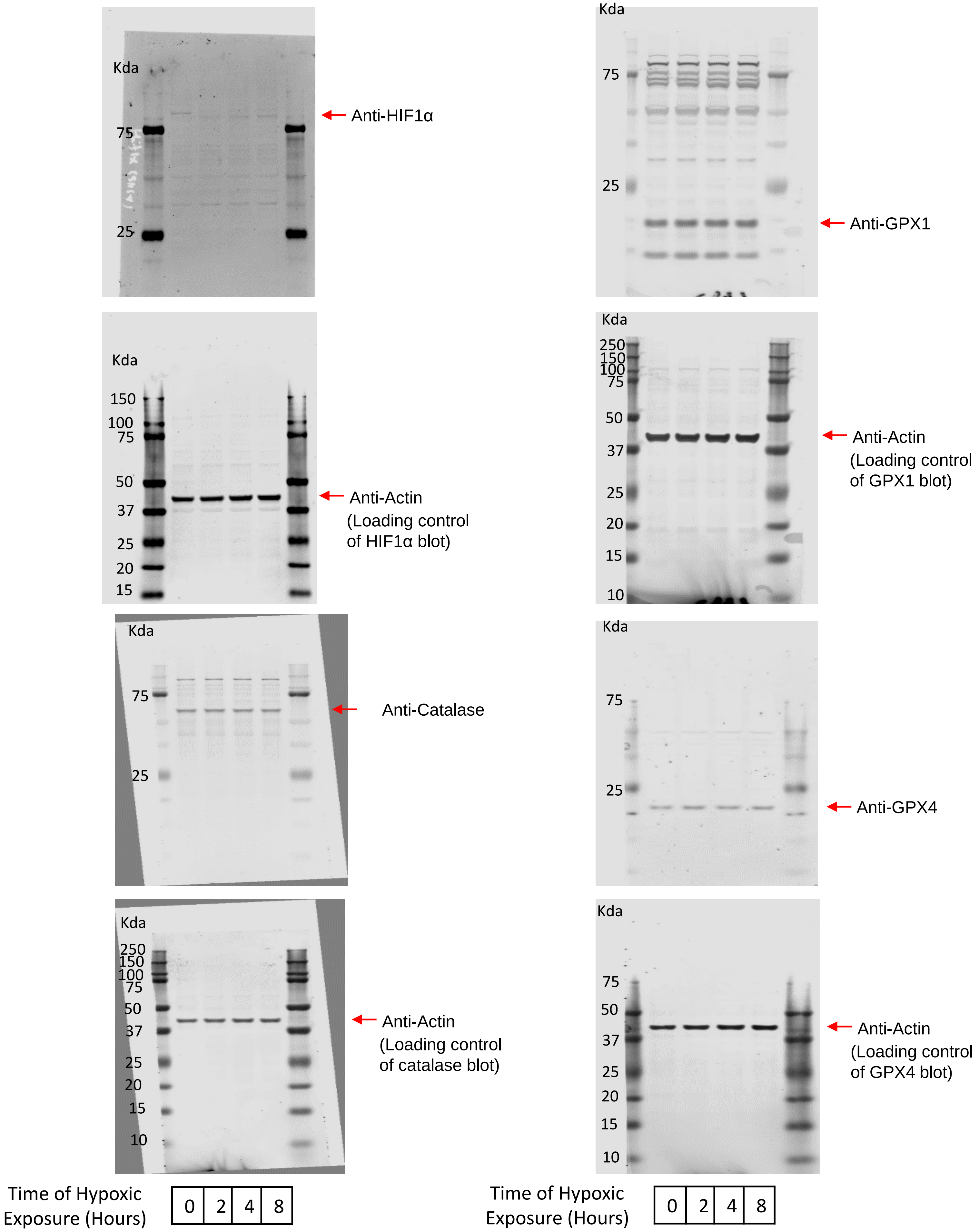

Kda
75
25
Anti-HIF1α
150
100
50
37
20
15
Anti-Actin
(Loading control
of HIF1α blot)
Anti-Catalase
250
10
(Loading control
of catalase blot)
Anti-GPX1
(Loading control
of GPX1 blot)
Anti-GPX4
(Loading control
of GPX4 blot)
Time of Hypoxic
Exposure (Hours)
0
2
4
8

Figure S1A

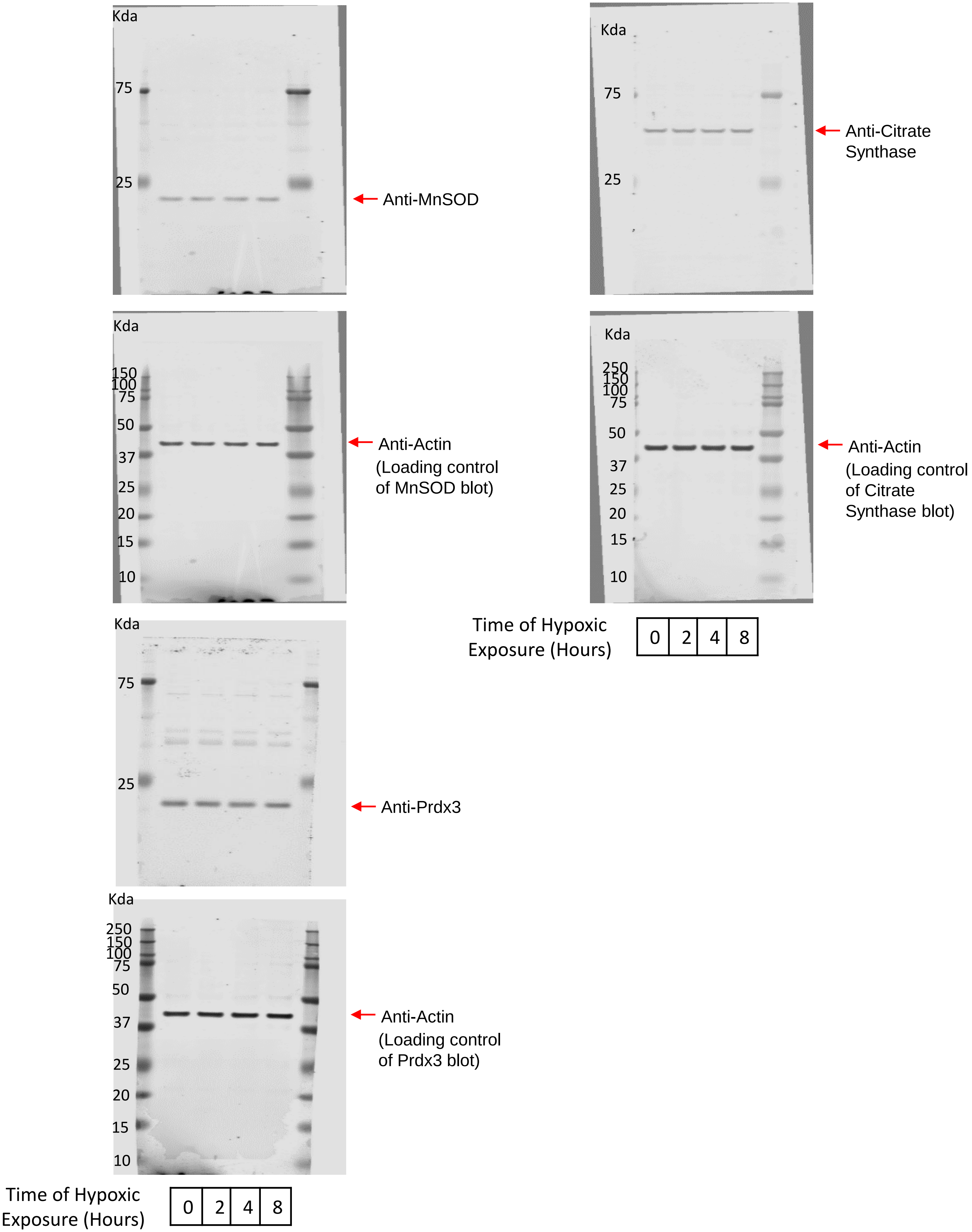
Kda
75
25
Anti-MnSOD
Kda
75
25
Anti-Citrate Synthase
Kda
150
100
75
50
37
25
20
15
10
Anti-Actin
(Loading control of MnSOD blot)
Kda
250
150
100
75
50
37
25
20
15
10
Anti-Actin
(Loading control of Citrate Synthase blot)
Time of Hypoxic Exposure (Hours)
0
2
4
8
Kda
75
25
Anti-Prdx3
Kda
250
150
100
75
50
37
25
20
15
10
Anti-Actin
(Loading control of Prdx3 blot)
Time of Hypoxic Exposure (Hours)
0
2
4
8

Figure S1B
