# Supplementary material for: HIF1α stabilization in hypoxia is not oxidant-initiated
Source: eLife. 2021 Oct 1;10:e72873. doi: 10.7554/eLife.72873 (PMC8530508; doi:10.7554/eLife.72873)
Supplement: Figure 3—figure supplement 6—source data 3. [file elife-72873-fig3-figsupp6-data3.zip › Figure S8 - source data 3/Original Blots.pptx]

## Slide 1
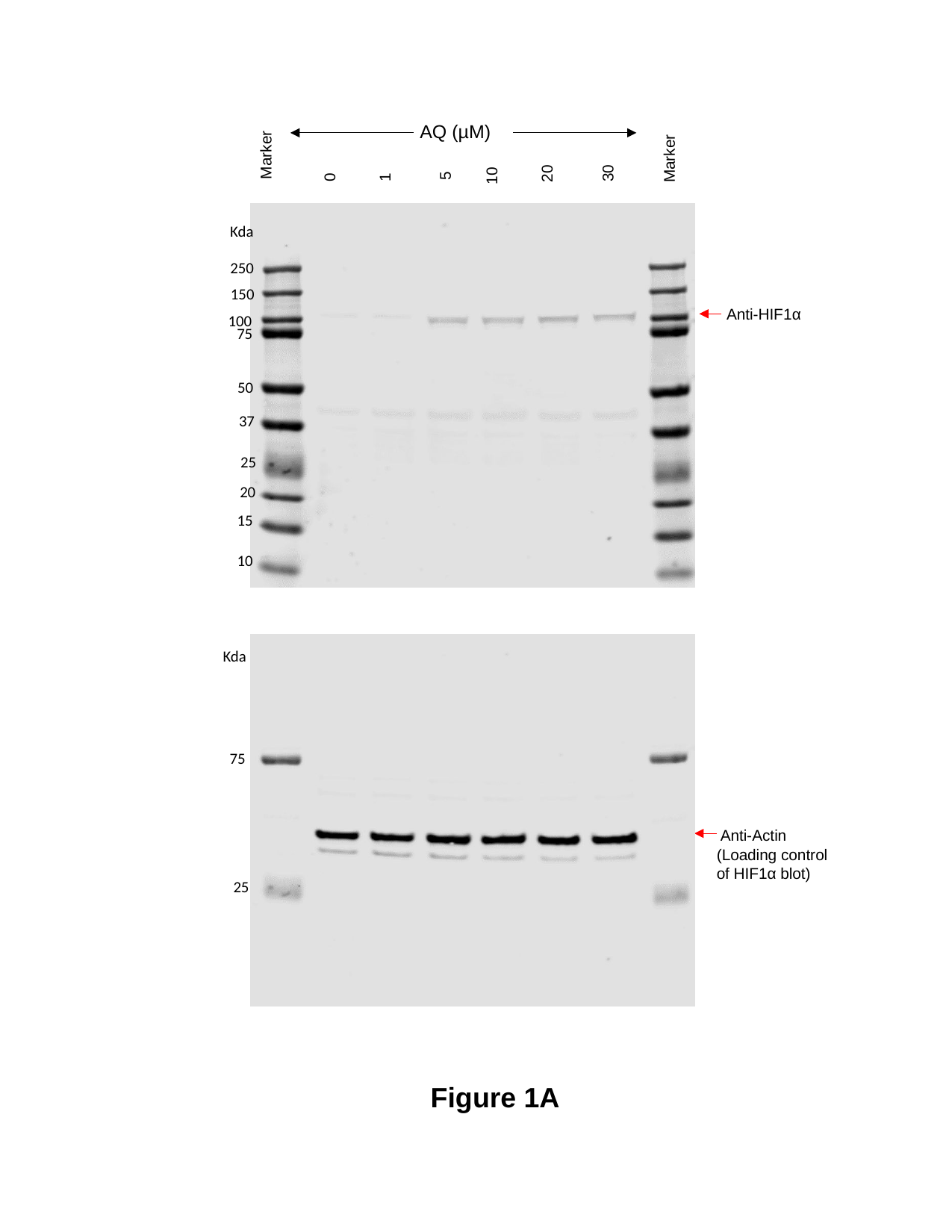

AQ (µM)
Marker
Marker
30
20
5
10
1
0
Kda
250
150
Anti-HIF1α
100
75
50
37
25
20
15
10
Kda
75
Anti-Actin
(Loading control
of HIF1α blot)
25
Figure 1A

## Slide 2
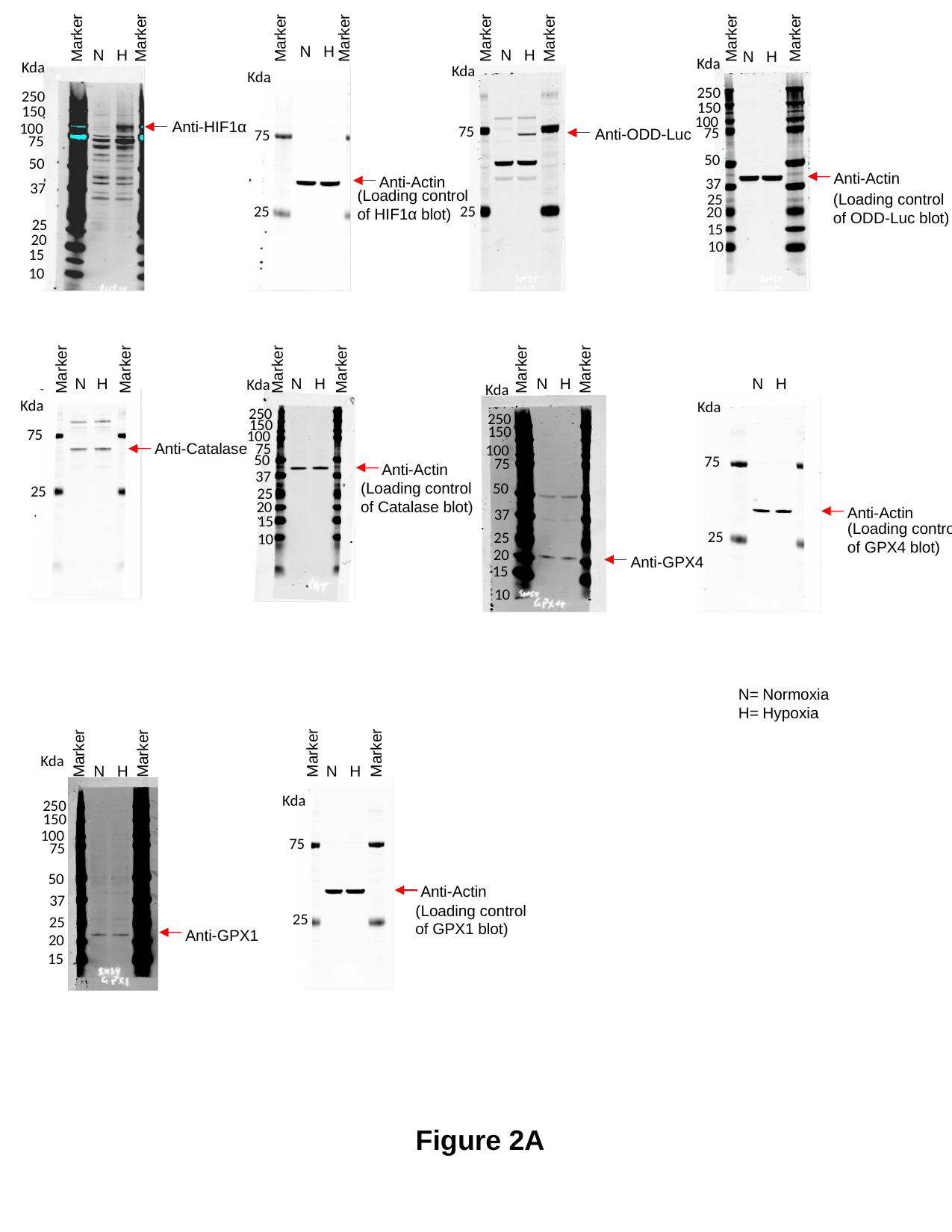

Marker
Marker
Marker
Marker
Marker
Marker
Marker
Marker
N
H
N
H
N
H
N
H
Kda
Kda
Kda
Kda
250
250
150
150
100
Anti-HIF1α
100
75
75
Anti-ODD-Luc
75
75
50
50
Anti-Actin
Anti-Actin
37
37
(Loading control
of HIF1α blot)
25
(Loading control
of ODD-Luc blot)
25
25
20
25
15
20
10
15
10
Marker
Marker
Marker
Marker
Marker
Marker
N
H
N
H
N
H
N
H
Kda
Kda
Kda
Kda
250
250
150
150
75
100
75
Anti-Catalase
100
50
75
75
Anti-Actin
37
50
(Loading control
of Catalase blot)
25
25
20
Anti-Actin
37
15
(Loading control
of GPX4 blot)
25
25
10
20
Anti-GPX4
15
10
N= Normoxia
H= Hypoxia
Marker
Marker
Marker
Marker
Kda
N
H
N
H
Kda
250
150
100
75
75
50
Anti-Actin
37
(Loading control
of GPX1 blot)
25
25
Anti-GPX1
20
15
Figure 2A

## Slide 3
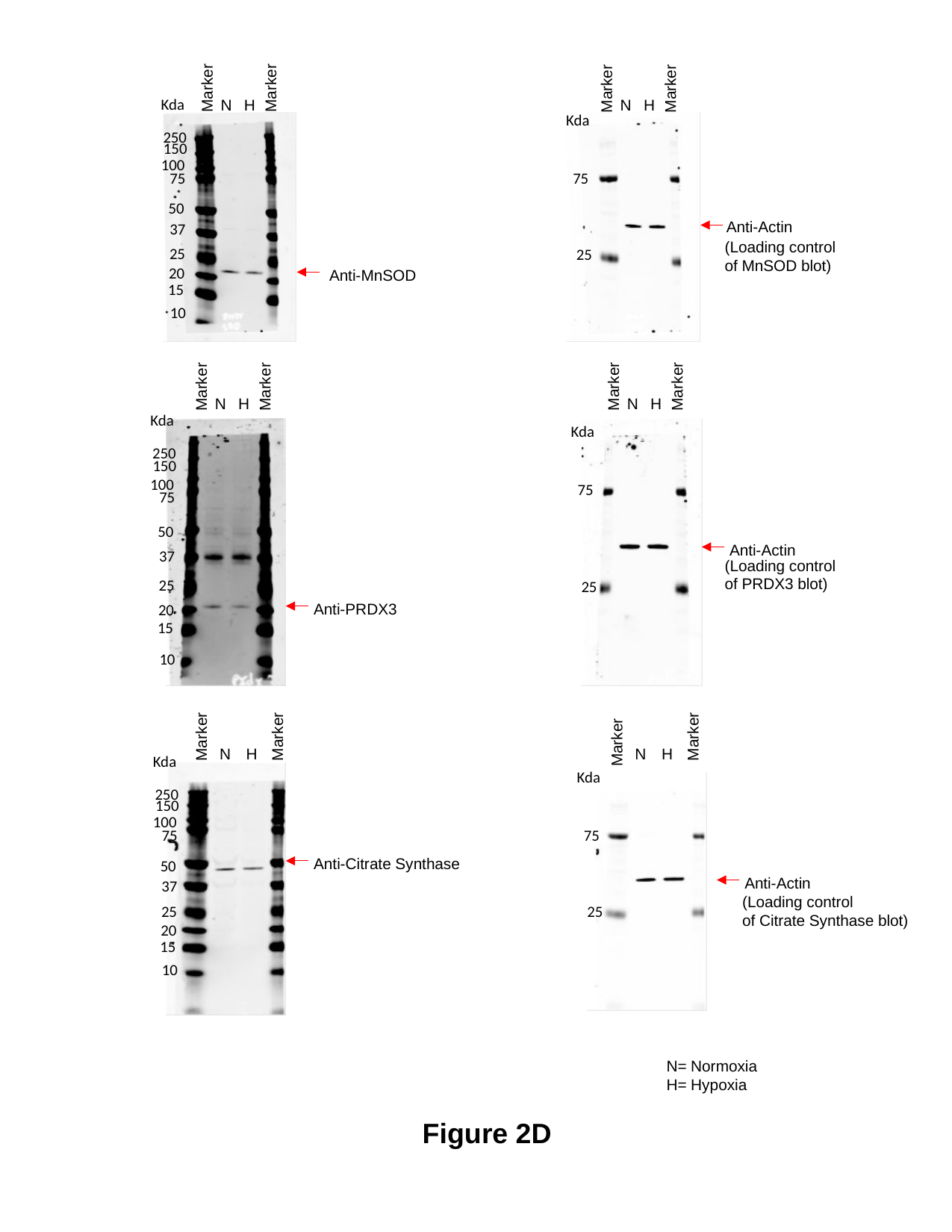

Marker
Marker
Marker
Marker
Kda
N
H
N
H
Kda
250
150
100
75
75
50
Anti-Actin
37
(Loading control
of MnSOD blot)
25
25
20
Anti-MnSOD
15
10
Marker
Marker
Marker
Marker
N
H
N
H
Kda
Kda
250
150
100
75
75
50
Anti-Actin
37
(Loading control
of PRDX3 blot)
25
25
Anti-PRDX3
20
15
10
Marker
Marker
Marker
Marker
N
H
N
H
Kda
Kda
250
150
100
75
75
Anti-Citrate Synthase
50
Anti-Actin
37
(Loading control
of Citrate Synthase blot)
25
25
20
15
10
N= Normoxia
H= Hypoxia
Figure 2D

## Slide 4
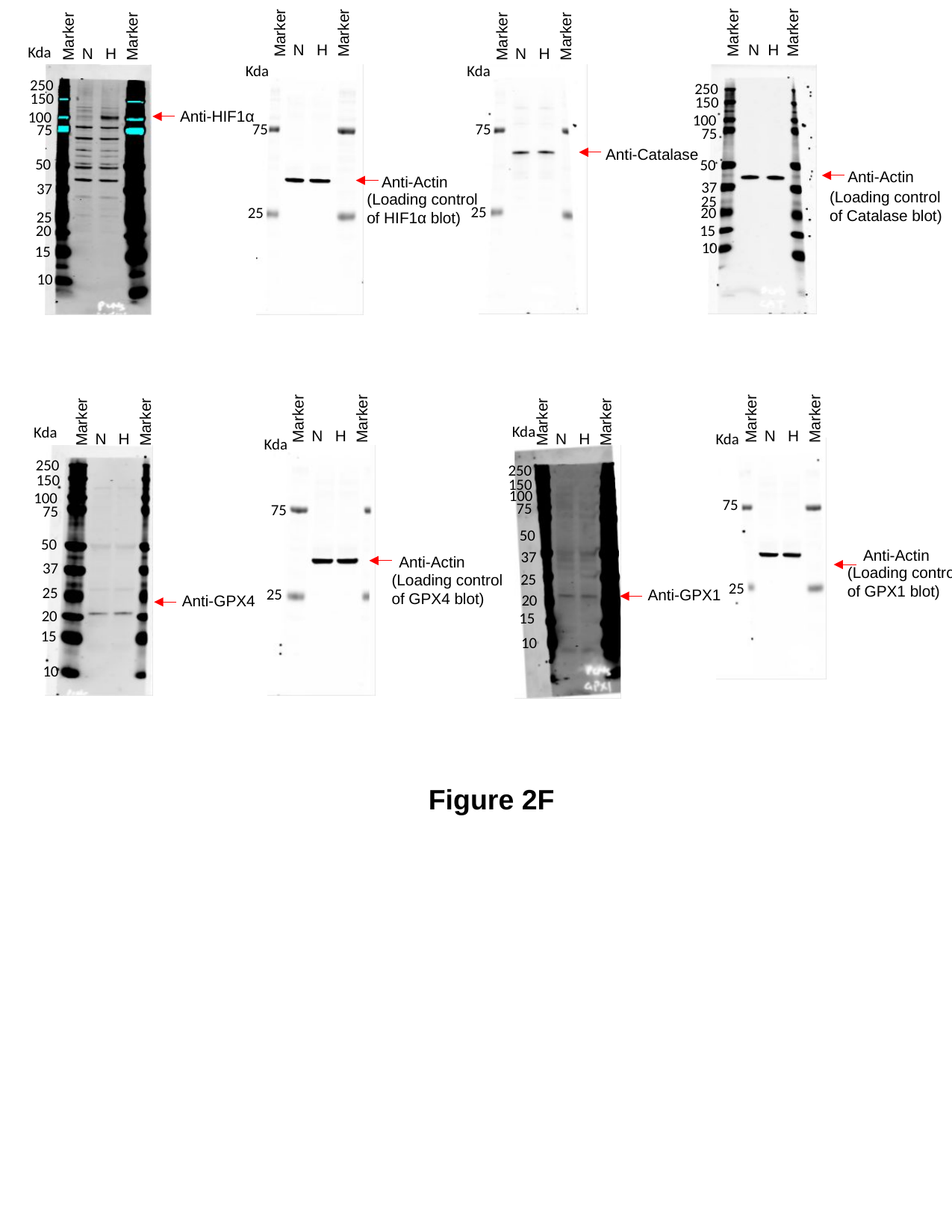

Marker
Marker
Marker
Marker
Marker
Marker
Marker
Marker
N
H
N
H
Kda
N
H
N
H
Kda
Kda
250
250
150
150
Anti-HIF1α
100
100
75
75
75
Kda
75
Anti-Catalase
50
50
Anti-Actin
Anti-Actin
37
37
75
(Loading control
of Catalase blot)
(Loading control
of HIF1α blot)
25
25
25
20
25
20
15
10
15
25
10
Marker
Marker
Marker
Marker
Marker
Marker
Marker
Marker
Kda
Kda
N
H
N
H
Kda
N
H
N
H
Kda
250
250
150
150
100
100
75
75
75
75
50
50
Anti-Actin
37
Anti-Actin
37
(Loading control
of GPX1 blot)
25
(Loading control
of GPX4 blot)
25
25
25
Anti-GPX1
20
Anti-GPX4
20
15
15
10
10
Figure 2F

## Slide 5
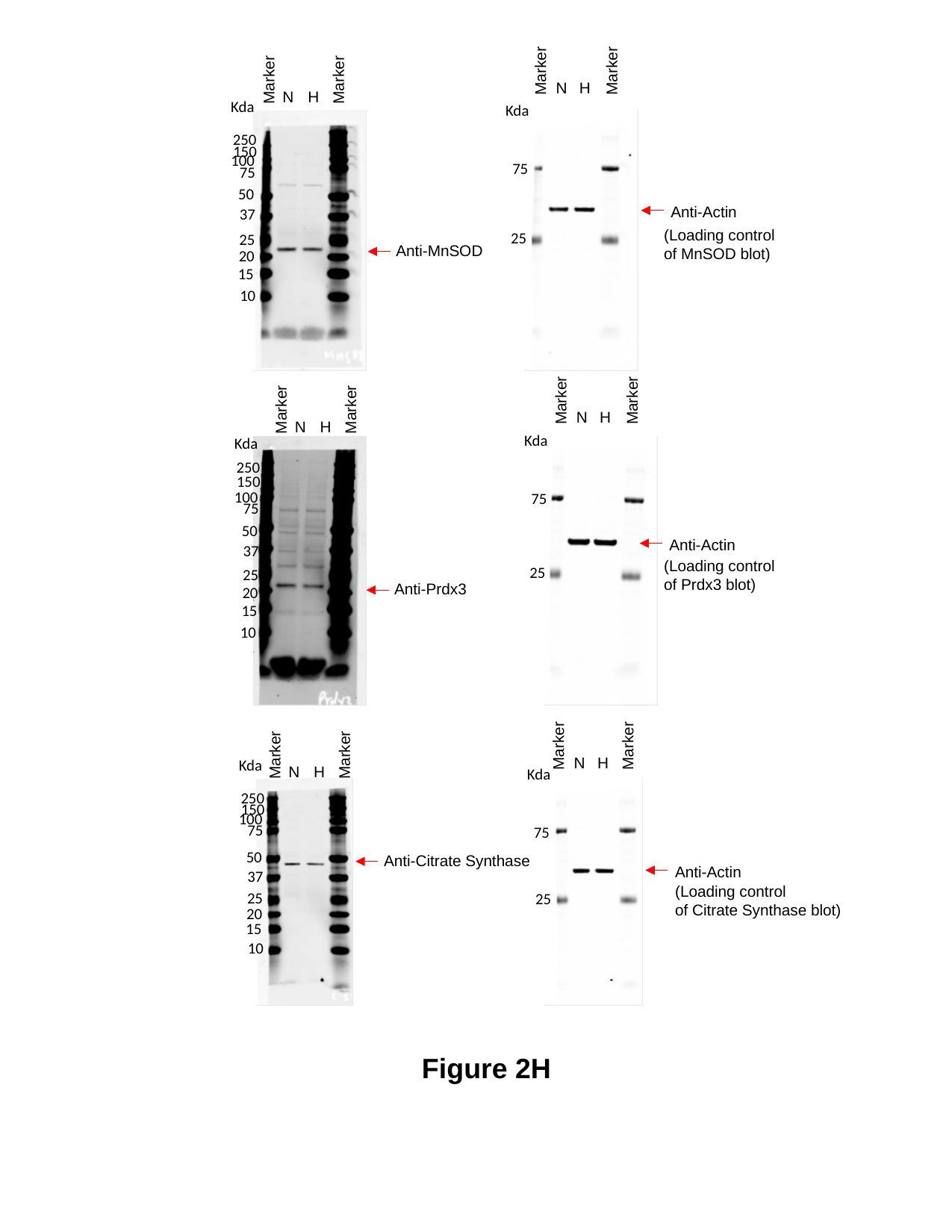

Marker
Marker
Marker
Marker
N
H
N
H
Kda
Kda
250
150
100
75
75
50
Anti-Actin
37
(Loading control
of MnSOD blot)
25
25
Anti-MnSOD
20
15
10
Marker
Marker
Marker
Marker
N
H
N
H
Kda
Kda
250
150
100
75
75
50
Anti-Actin
37
(Loading control
of Prdx3 blot)
25
25
Anti-Prdx3
20
15
10
Marker
Marker
Marker
Marker
N
H
Kda
N
H
Kda
250
150
100
75
75
50
Anti-Citrate Synthase
Anti-Actin
37
(Loading control
of Citrate Synthase blot)
25
25
20
15
10
Figure 2H

## Slide 6
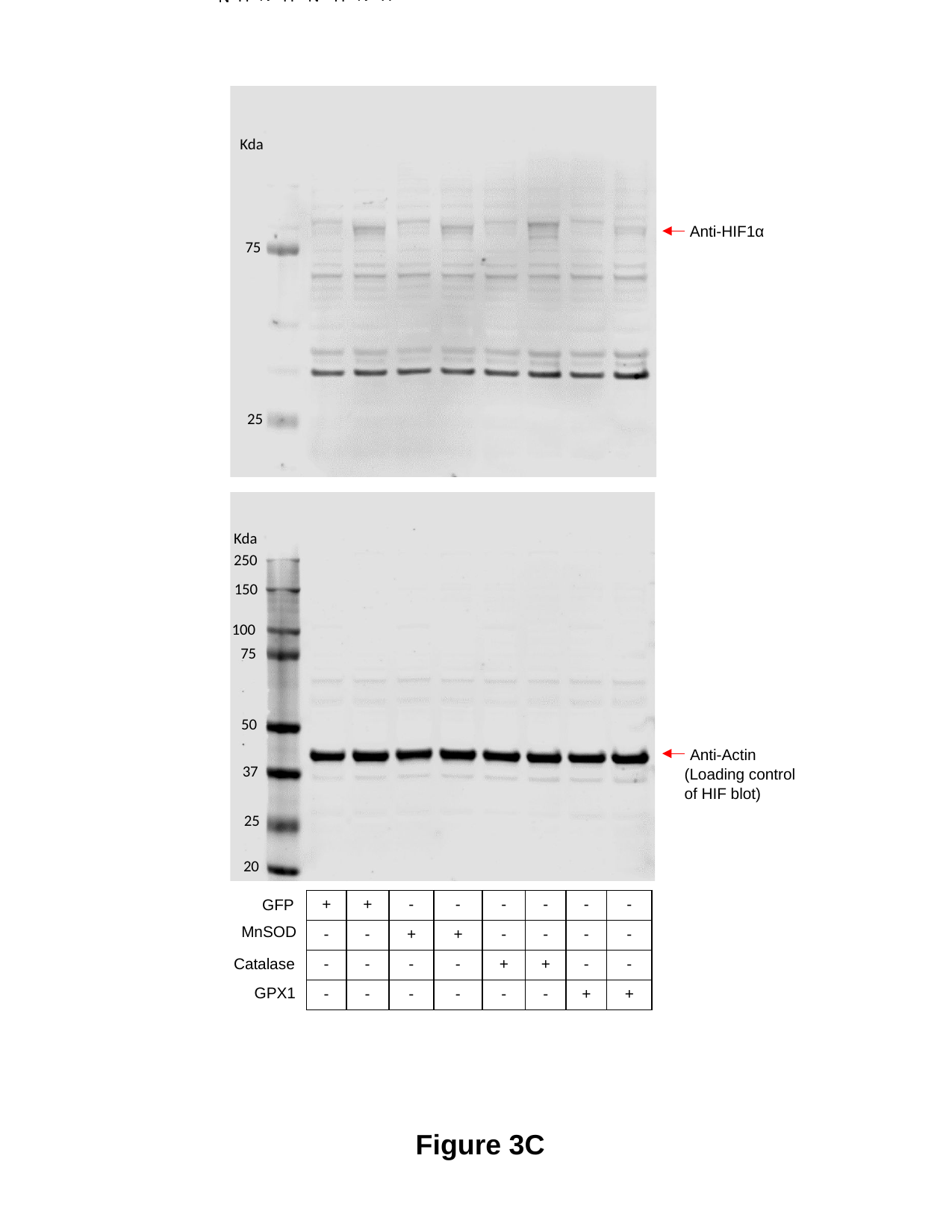

H
N
N
H
N
H
H
N
Kda
Anti-HIF1α
75
25
Kda
250
150
100
75
50
Anti-Actin
37
(Loading control of HIF blot)
25
20
GFP
| + | + | - | - | - | - | - | - |
| --- | --- | --- | --- | --- | --- | --- | --- |
| - | - | + | + | - | - | - | - |
| - | - | - | - | + | + | - | - |
| - | - | - | - | - | - | + | + |
MnSOD
Catalase
GPX1
Figure 3C

## Slide 7
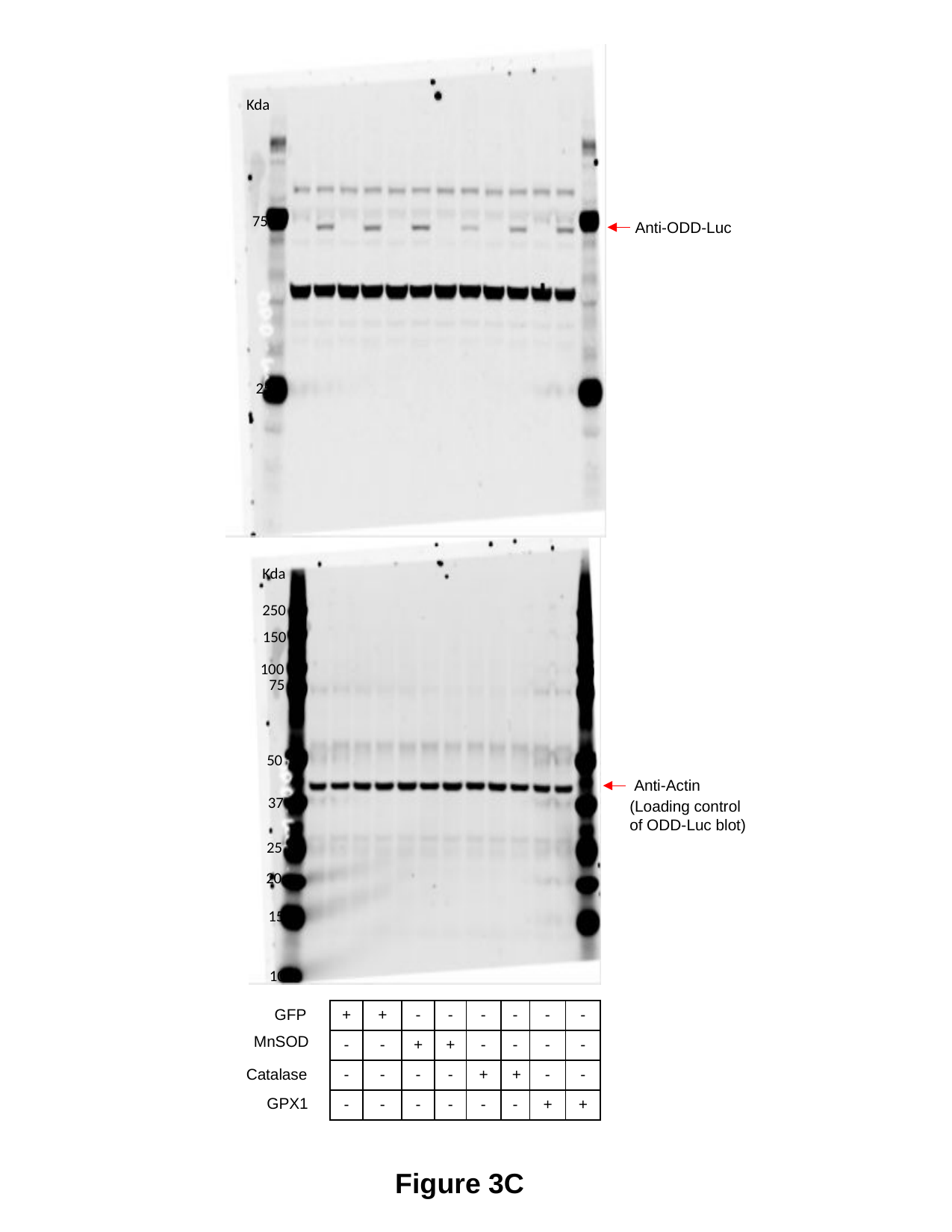

Kda
75
Anti-ODD-Luc
25
Kda
250
150
100
75
50
Anti-Actin
37
(Loading control of ODD-Luc blot)
25
20
15
10
GFP
| + | + | - | - | - | - | - | - |
| --- | --- | --- | --- | --- | --- | --- | --- |
| - | - | + | + | - | - | - | - |
| - | - | - | - | + | + | - | - |
| - | - | - | - | - | - | + | + |
MnSOD
Catalase
GPX1
Figure 3C

## Slide 8
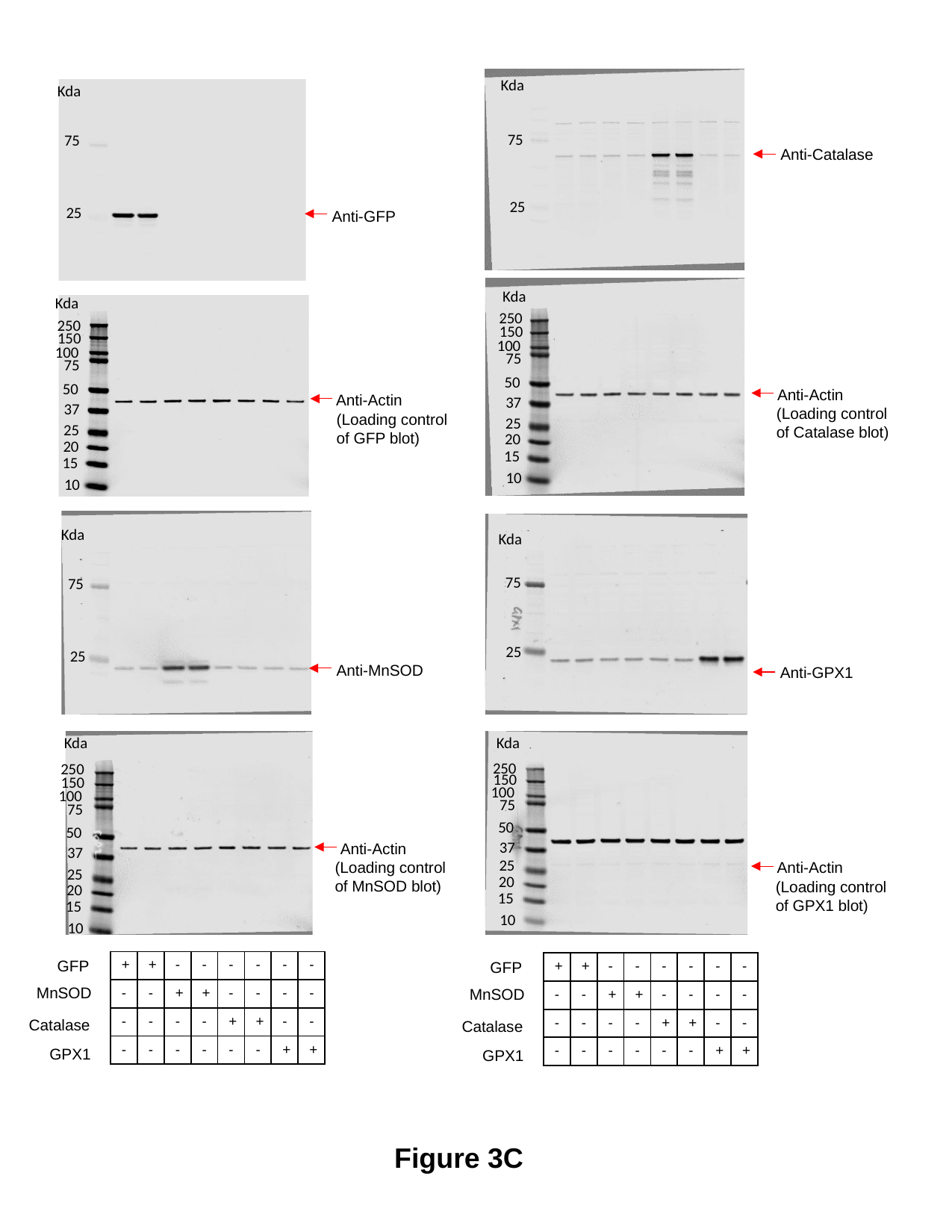

Kda
Kda
75
75
Anti-Catalase
25
25
Anti-GFP
Kda
Kda
250
250
150
150
100
100
75
75
50
50
Anti-Actin
Anti-Actin
37
37
(Loading control of Catalase blot)
(Loading control
of GFP blot)
25
25
20
20
15
15
10
10
Kda
Kda
75
75
25
25
Anti-MnSOD
Anti-GPX1
Kda
Kda
250
250
150
150
100
100
75
75
50
50
37
Anti-Actin
37
25
(Loading control of MnSOD blot)
Anti-Actin
25
20
(Loading control
of GPX1 blot)
20
15
15
10
10
GFP
| + | + | - | - | - | - | - | - |
| --- | --- | --- | --- | --- | --- | --- | --- |
| - | - | + | + | - | - | - | - |
| - | - | - | - | + | + | - | - |
| - | - | - | - | - | - | + | + |
GFP
| + | + | - | - | - | - | - | - |
| --- | --- | --- | --- | --- | --- | --- | --- |
| - | - | + | + | - | - | - | - |
| - | - | - | - | + | + | - | - |
| - | - | - | - | - | - | + | + |
MnSOD
MnSOD
Catalase
Catalase
GPX1
GPX1
Figure 3C

## Slide 9
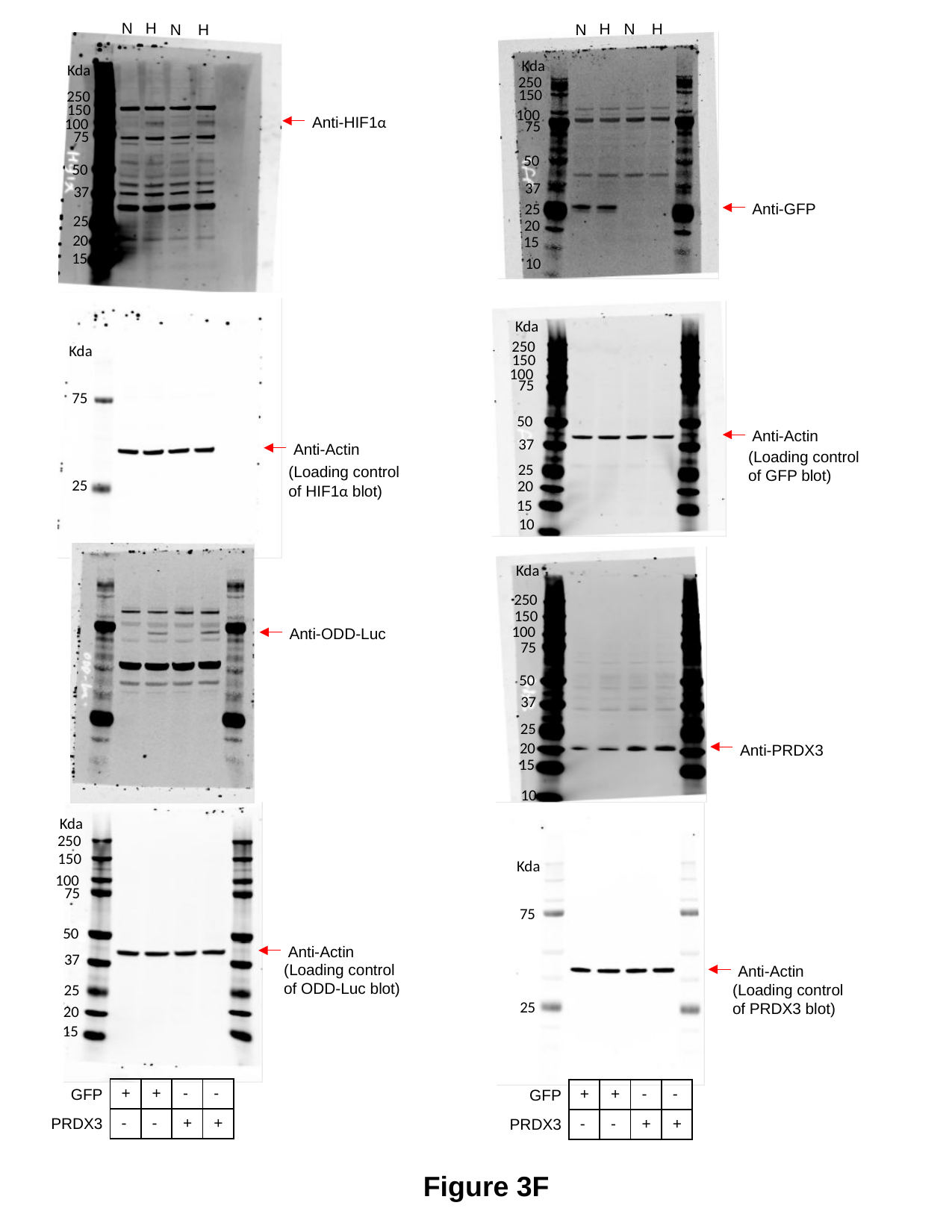

H
N
H
N
H
H
N
N
Kda
Kda
250
150
250
150
100
Anti-HIF1α
100
75
75
50
50
37
37
Anti-GFP
25
25
20
20
15
15
10
Kda
250
Kda
150
100
75
75
50
Anti-Actin
37
Anti-Actin
(Loading control
of GFP blot)
25
(Loading control
of HIF1α blot)
25
20
15
10
Kda
250
150
100
Anti-ODD-Luc
75
50
37
25
20
Anti-PRDX3
15
10
Kda
250
150
Kda
100
75
75
50
Anti-Actin
37
(Loading control
of ODD-Luc blot)
Anti-Actin
25
(Loading control
of PRDX3 blot)
25
20
15
| + | + | - | - |
| --- | --- | --- | --- |
| - | - | + | + |
GFP
| + | + | - | - |
| --- | --- | --- | --- |
| - | - | + | + |
GFP
PRDX3
PRDX3
Figure 3F

## Slide 10
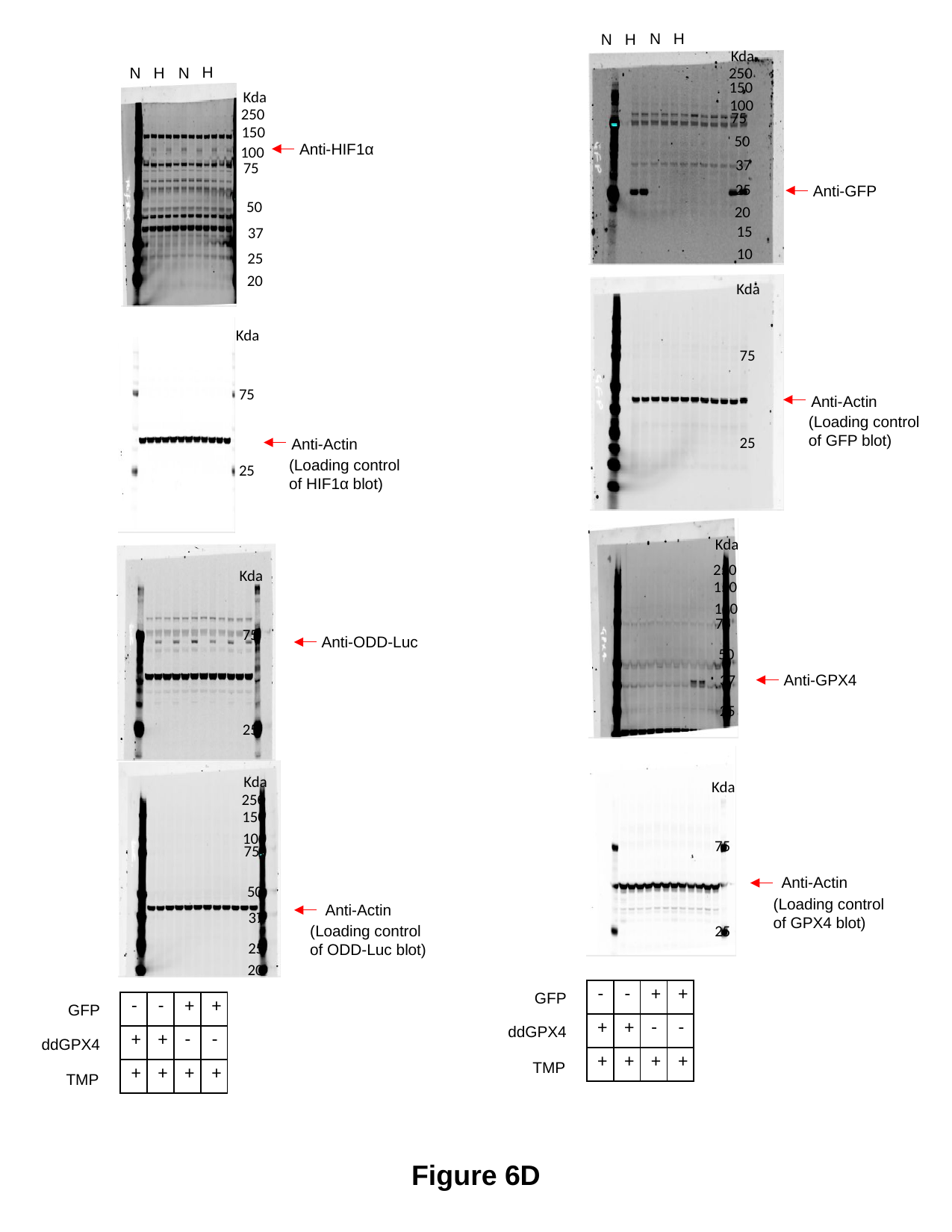

H
N
H
N
Kda
H
N
H
250
N
150
Kda
100
250
75
150
50
Anti-HIF1α
100
37
75
25
Anti-GFP
50
20
15
37
10
25
20
Kda
Kda
75
75
Anti-Actin
(Loading control
of GFP blot)
25
Anti-Actin
(Loading control
of HIF1α blot)
25
Kda
250
Kda
150
100
75
75
Anti-ODD-Luc
50
37
Anti-GPX4
25
25
Kda
Kda
250
150
100
75
75
Anti-Actin
50
(Loading control
of GPX4 blot)
Anti-Actin
37
(Loading control
of ODD-Luc blot)
25
25
20
| - | - | + | + |
| --- | --- | --- | --- |
| + | + | - | - |
| + | + | + | + |
GFP
| - | - | + | + |
| --- | --- | --- | --- |
| + | + | - | - |
| + | + | + | + |
GFP
ddGPX4
ddGPX4
TMP
TMP
Figure 6D

## Slide 11
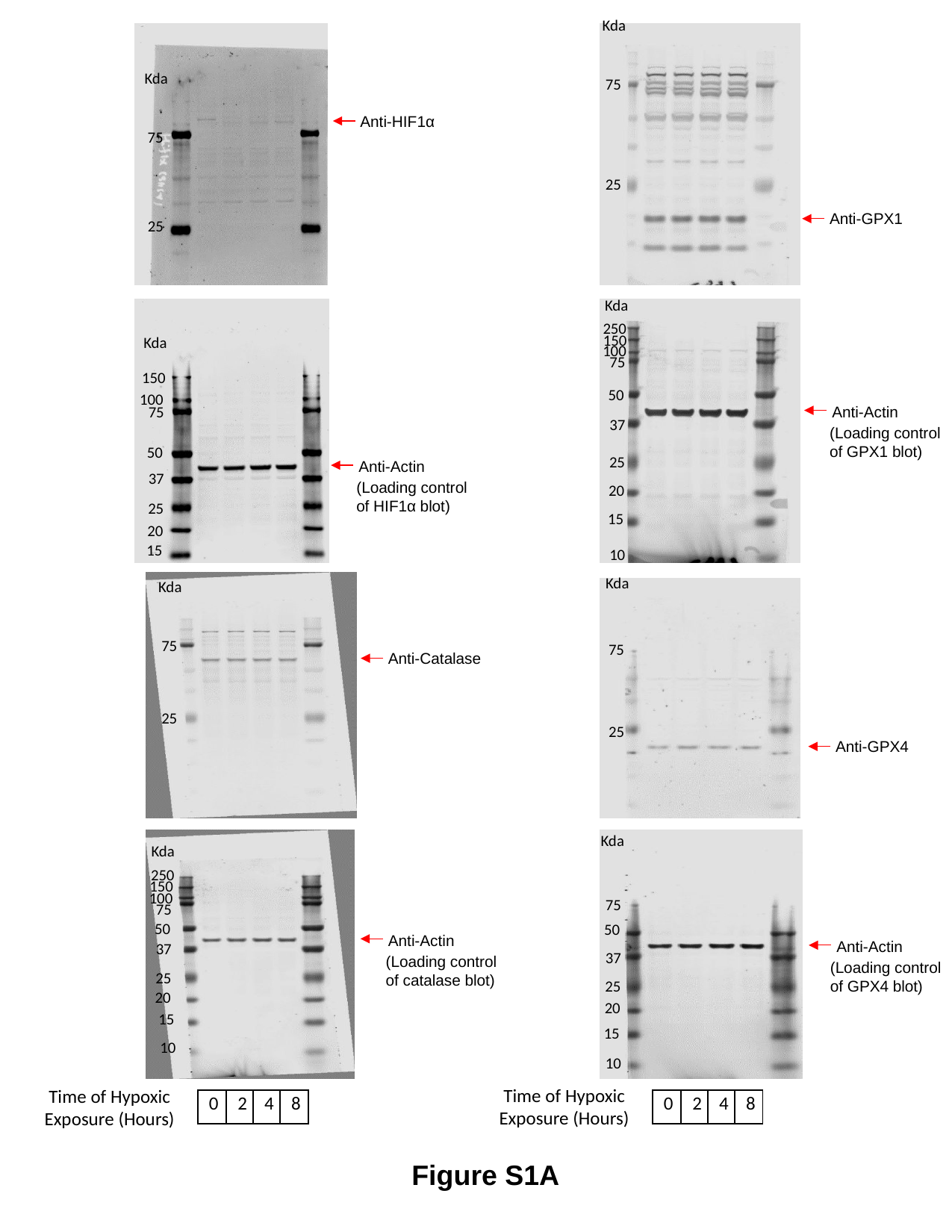

Kda
Kda
75
Anti-HIF1α
75
25
Anti-GPX1
25
Kda
250
150
Kda
100
75
150
50
100
Anti-Actin
75
37
(Loading control
of GPX1 blot)
50
25
Anti-Actin
37
(Loading control
of HIF1α blot)
20
25
15
20
15
10
Kda
Kda
75
75
Anti-Catalase
25
25
Anti-GPX4
Kda
Kda
250
150
100
75
75
50
50
Anti-Actin
Anti-Actin
37
37
(Loading control
of catalase blot)
(Loading control
of GPX4 blot)
25
25
20
20
15
15
10
10
Time of Hypoxic
Exposure (Hours)
Time of Hypoxic
Exposure (Hours)
| 0 | 2 | 4 | 8 |
| --- | --- | --- | --- |
| 0 | 2 | 4 | 8 |
| --- | --- | --- | --- |
Figure S1A

## Slide 12
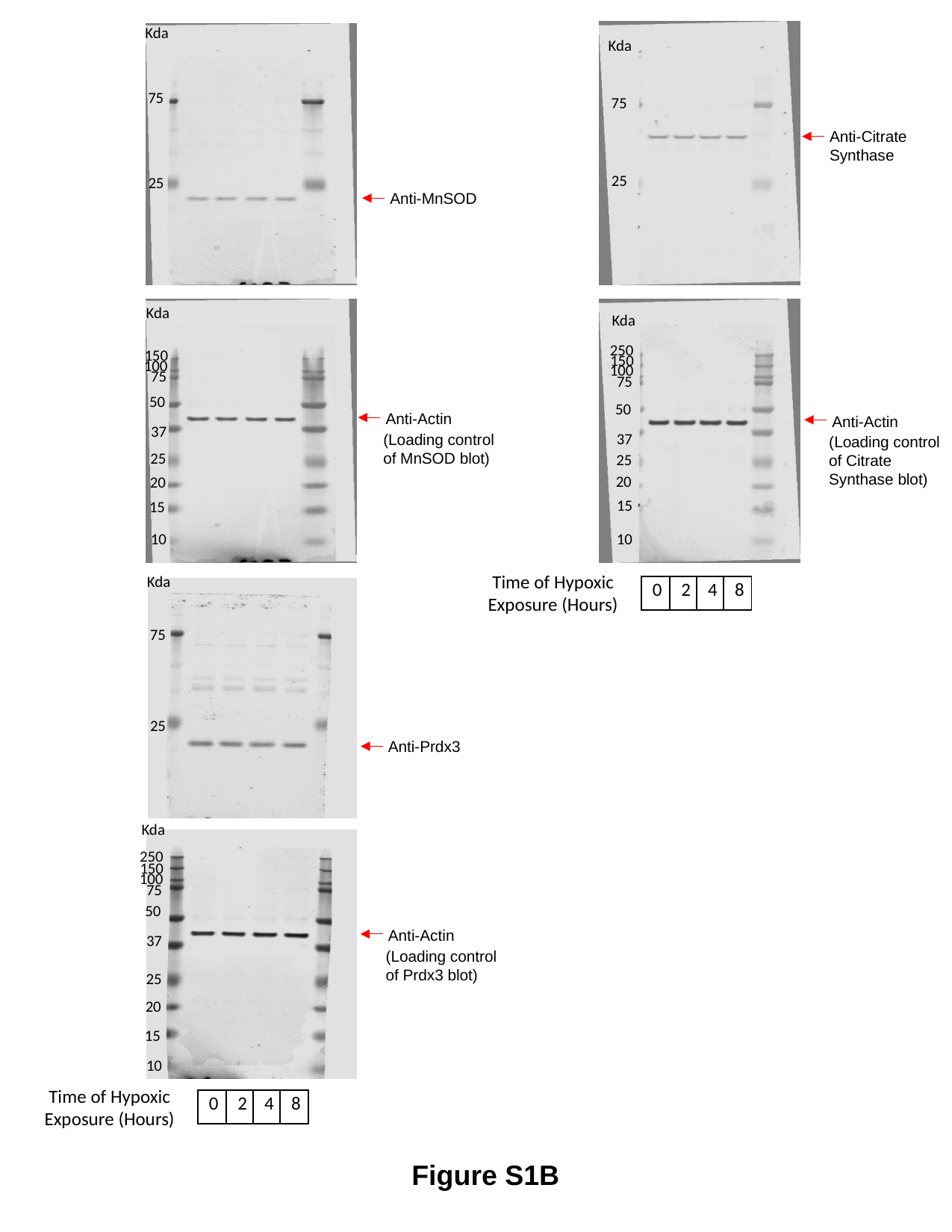

Kda
Kda
75
75
Anti-Citrate
Synthase
25
25
Anti-MnSOD
Kda
Kda
250
150
150
100
100
75
75
50
50
Anti-Actin
Anti-Actin
37
37
(Loading control
of MnSOD blot)
(Loading control
of Citrate
Synthase blot)
25
25
20
20
15
15
10
10
Time of Hypoxic
Exposure (Hours)
Kda
| 0 | 2 | 4 | 8 |
| --- | --- | --- | --- |
75
25
Anti-Prdx3
Kda
250
150
100
75
50
Anti-Actin
37
(Loading control
of Prdx3 blot)
25
20
15
10
Time of Hypoxic
Exposure (Hours)
| 0 | 2 | 4 | 8 |
| --- | --- | --- | --- |
Figure S1B

## Slide 13
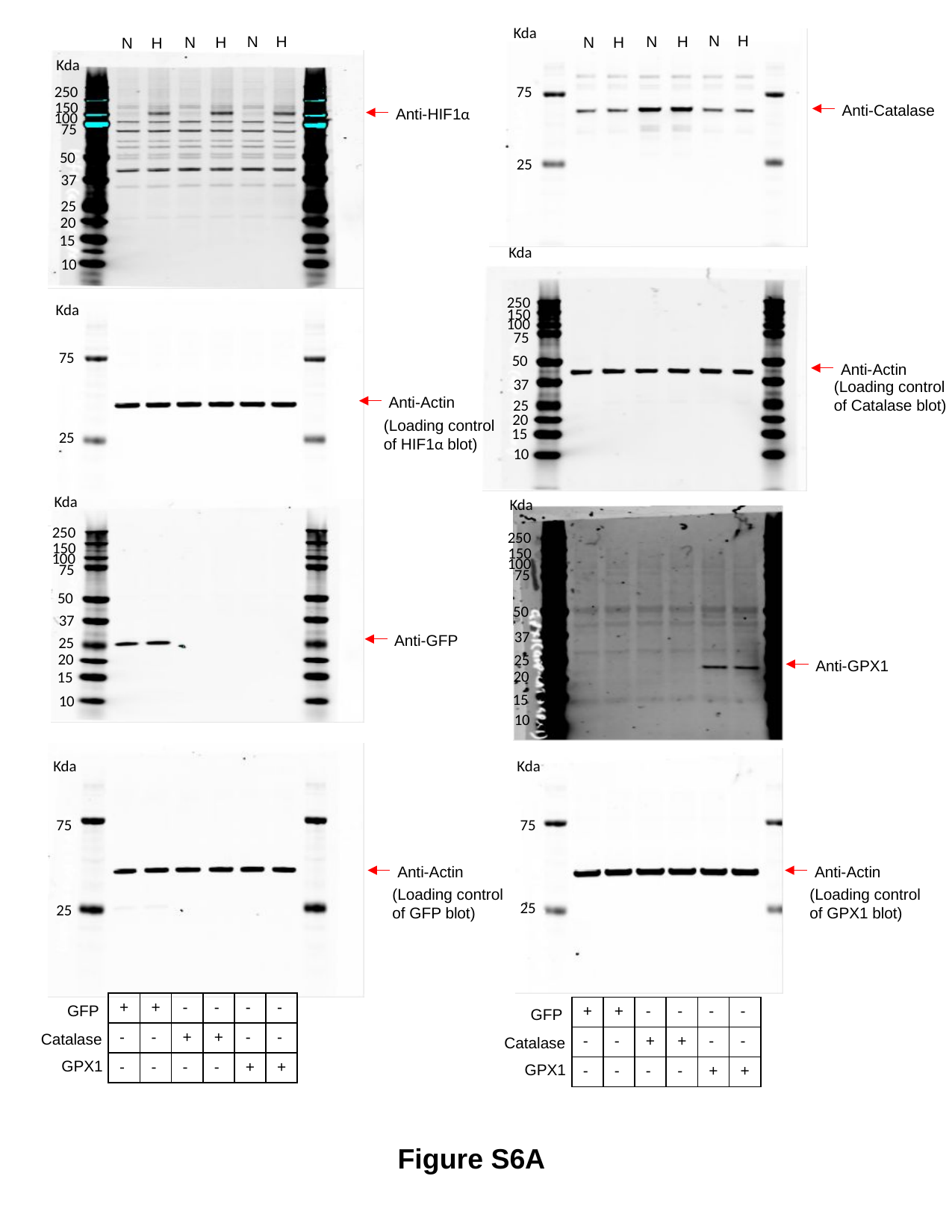

Kda
H
N
H
N
H
N
H
H
N
N
H
N
Kda
250
75
150
Anti-Catalase
Anti-HIF1α
100
75
50
25
37
25
20
15
Kda
10
250
Kda
150
100
75
75
50
Anti-Actin
37
(Loading control
of Catalase blot)
Anti-Actin
25
20
(Loading control
of HIF1α blot)
15
25
10
Kda
Kda
250
250
150
150
100
100
75
75
50
50
37
37
Anti-GFP
25
20
25
Anti-GPX1
20
15
15
10
10
Kda
Kda
75
75
Anti-Actin
Anti-Actin
(Loading control
of GFP blot)
(Loading control
of GPX1 blot)
25
25
| + | + | - | - | - | - |
| --- | --- | --- | --- | --- | --- |
| - | - | + | + | - | - |
| - | - | - | - | + | + |
GFP
| + | + | - | - | - | - |
| --- | --- | --- | --- | --- | --- |
| - | - | + | + | - | - |
| - | - | - | - | + | + |
GFP
Catalase
Catalase
GPX1
GPX1
Figure S6A

## Slide 14
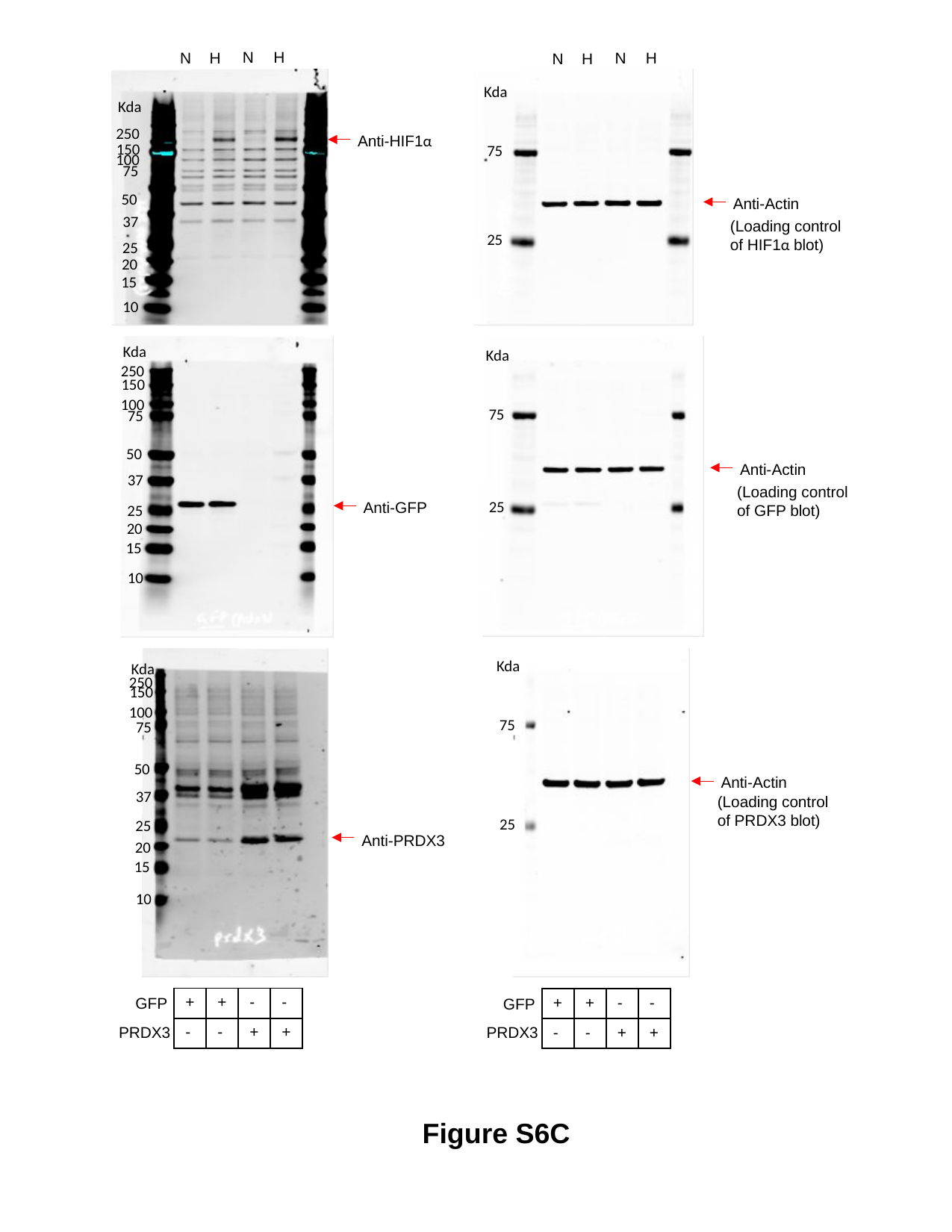

H
N
H
H
N
N
H
N
Kda
Kda
250
Anti-HIF1α
150
75
100
75
50
Anti-Actin
37
(Loading control
of HIF1α blot)
25
25
20
15
10
Kda
Kda
250
150
100
75
75
50
Anti-Actin
37
(Loading control
of GFP blot)
25
Anti-GFP
25
20
15
10
Kda
Kda
250
150
100
75
75
50
Anti-Actin
37
(Loading control
of PRDX3 blot)
25
25
Anti-PRDX3
20
15
10
GFP
| + | + | - | - |
| --- | --- | --- | --- |
| - | - | + | + |
GFP
| + | + | - | - |
| --- | --- | --- | --- |
| - | - | + | + |
PRDX3
PRDX3
Figure S6C

## Slide 15
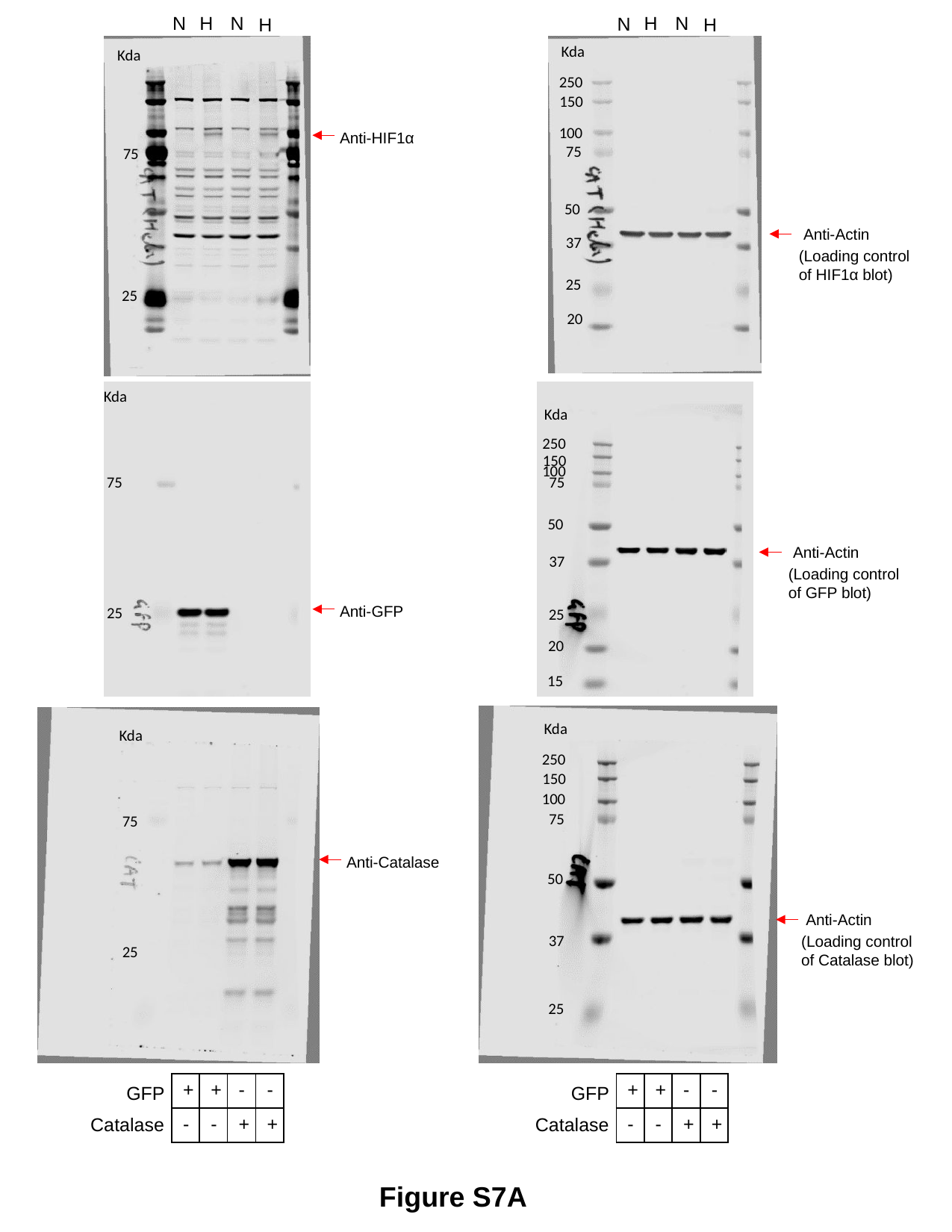

N
H
N
H
N
N
H
H
Kda
Kda
250
150
100
Anti-HIF1α
75
75
50
Anti-Actin
37
(Loading control
of HIF1α blot)
25
25
20
Kda
Kda
250
150
100
75
75
50
Anti-Actin
37
(Loading control
of GFP blot)
Anti-GFP
25
25
20
15
Kda
Kda
250
150
100
75
75
Anti-Catalase
50
Anti-Actin
37
(Loading control
of Catalase blot)
25
25
| + | + | - | - |
| --- | --- | --- | --- |
| - | - | + | + |
| + | + | - | - |
| --- | --- | --- | --- |
| - | - | + | + |
GFP
GFP
Catalase
Catalase
Figure S7A

## Slide 16
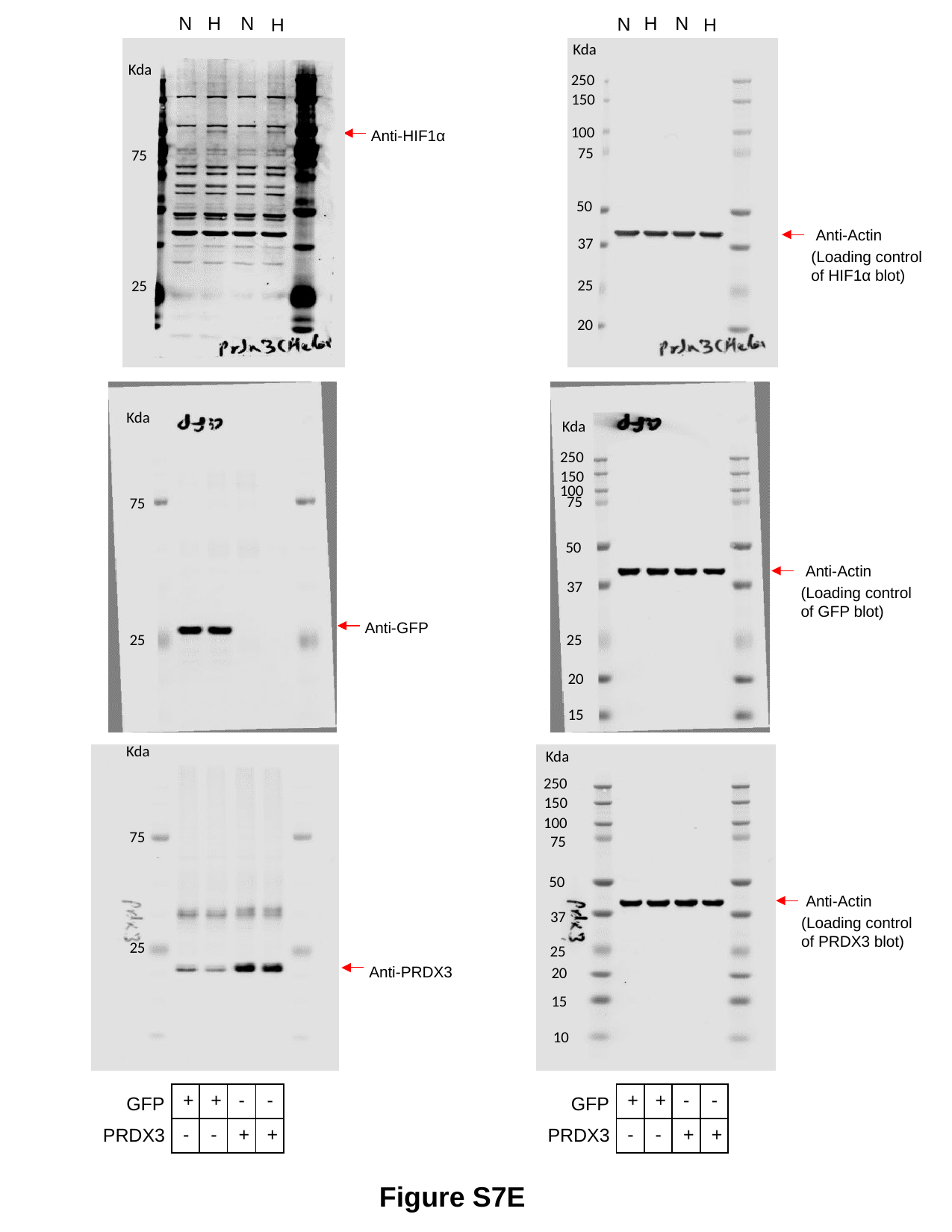

N
H
N
H
N
N
H
H
Kda
Kda
250
150
100
Anti-HIF1α
75
75
50
Anti-Actin
37
(Loading control
of HIF1α blot)
25
25
20
Kda
Kda
250
150
100
75
75
50
Anti-Actin
37
(Loading control
of GFP blot)
Anti-GFP
25
25
20
15
Kda
Kda
250
150
100
75
75
50
Anti-Actin
37
(Loading control
of PRDX3 blot)
25
25
Anti-PRDX3
20
15
10
| + | + | - | - |
| --- | --- | --- | --- |
| - | - | + | + |
| + | + | - | - |
| --- | --- | --- | --- |
| - | - | + | + |
GFP
GFP
PRDX3
PRDX3
Figure S7E

## Slide 17
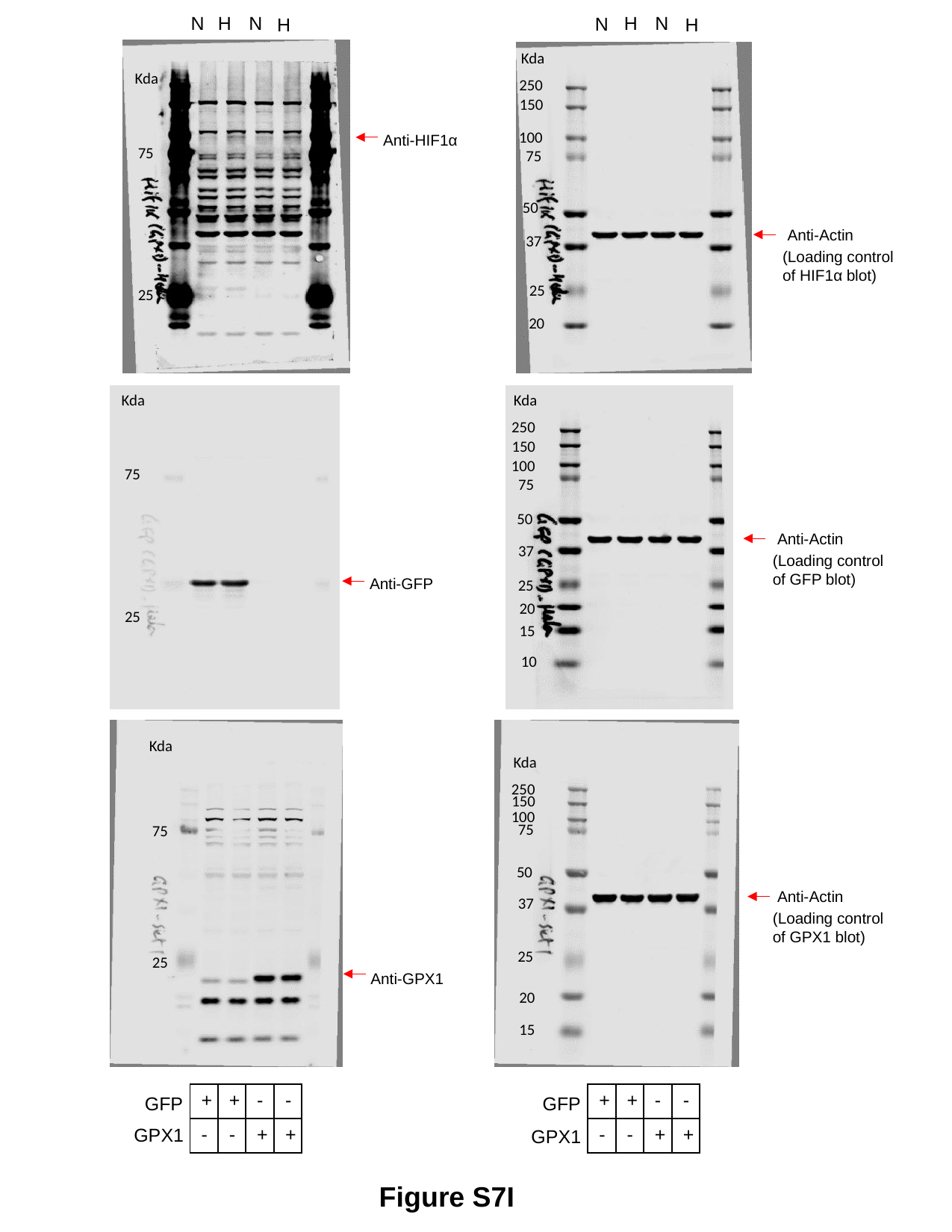

N
H
N
H
N
N
H
H
Kda
Kda
250
150
100
Anti-HIF1α
75
75
50
Anti-Actin
37
(Loading control
of HIF1α blot)
25
25
20
Kda
Kda
250
150
100
75
75
50
Anti-Actin
37
(Loading control
of GFP blot)
Anti-GFP
25
20
25
15
10
Kda
Kda
250
150
100
75
75
50
Anti-Actin
37
(Loading control
of GPX1 blot)
25
25
Anti-GPX1
20
15
| + | + | - | - |
| --- | --- | --- | --- |
| - | - | + | + |
| + | + | - | - |
| --- | --- | --- | --- |
| - | - | + | + |
GFP
GFP
GPX1
GPX1
Figure S7I

## Slide 18
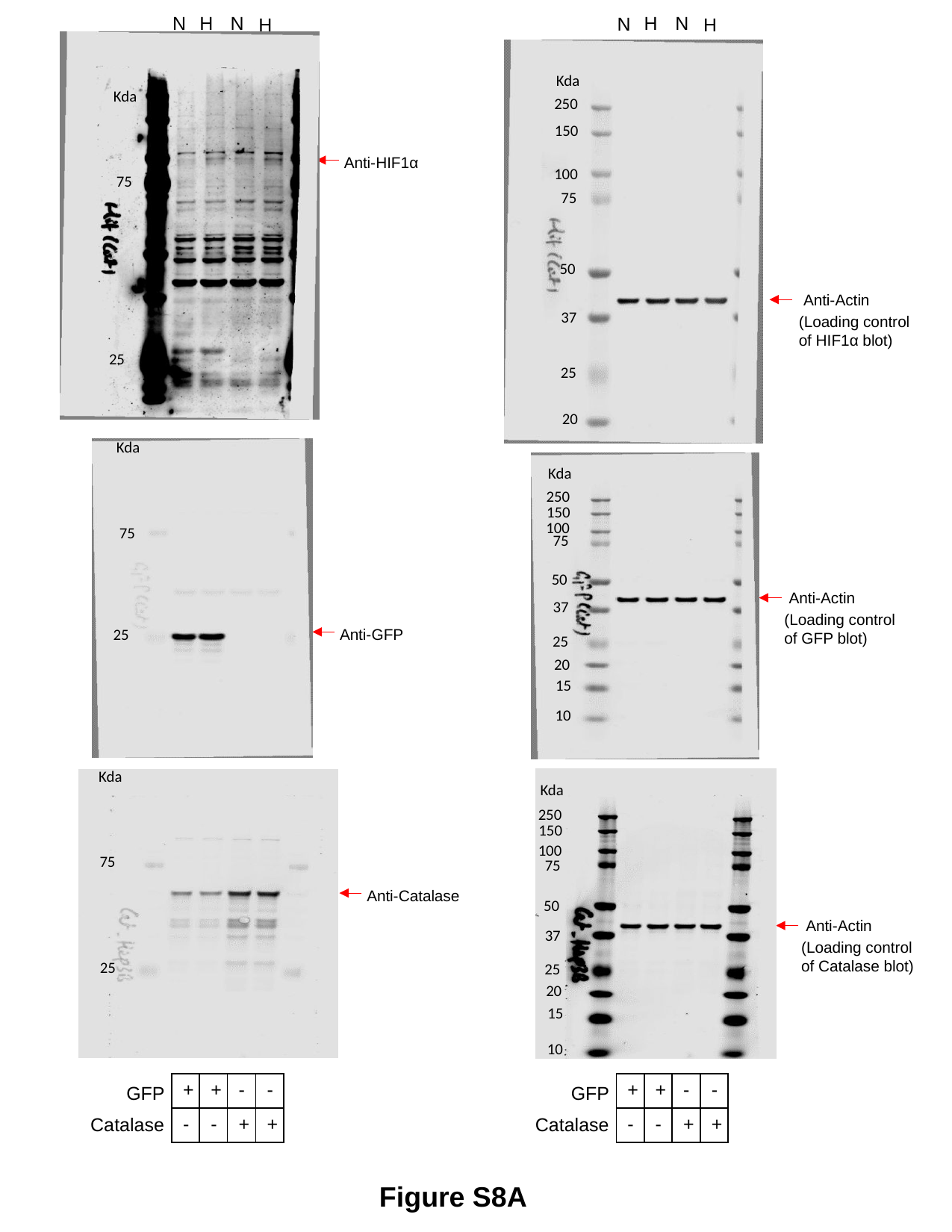

N
H
N
H
N
N
H
H
Kda
Kda
250
150
Anti-HIF1α
100
75
75
50
Anti-Actin
37
(Loading control
of HIF1α blot)
25
25
20
Kda
Kda
250
150
100
75
75
50
Anti-Actin
37
(Loading control
of GFP blot)
Anti-GFP
25
25
20
15
10
Kda
Kda
250
150
100
75
75
Anti-Catalase
50
Anti-Actin
37
(Loading control
of Catalase blot)
25
25
20
15
10
| + | + | - | - |
| --- | --- | --- | --- |
| - | - | + | + |
| + | + | - | - |
| --- | --- | --- | --- |
| - | - | + | + |
GFP
GFP
Catalase
Catalase
Figure S8A

## Slide 19
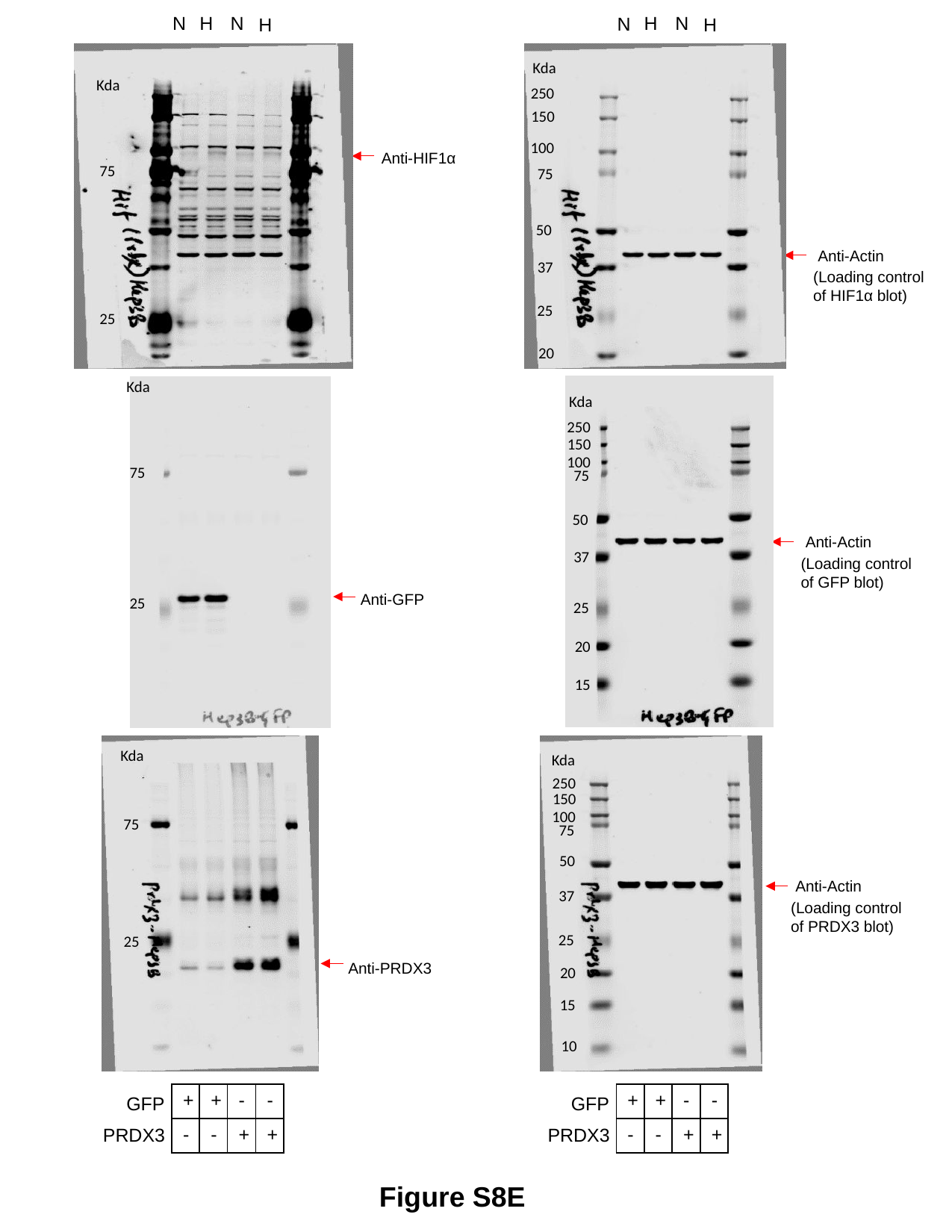

N
H
N
H
N
N
H
H
Kda
Kda
250
150
100
Anti-HIF1α
75
75
50
Anti-Actin
37
(Loading control
of HIF1α blot)
25
25
20
Kda
Kda
250
150
100
75
75
50
Anti-Actin
37
(Loading control
of GFP blot)
Anti-GFP
25
25
20
15
Kda
Kda
250
150
100
75
75
50
Anti-Actin
37
(Loading control
of PRDX3 blot)
25
25
Anti-PRDX3
20
15
10
| + | + | - | - |
| --- | --- | --- | --- |
| - | - | + | + |
| + | + | - | - |
| --- | --- | --- | --- |
| - | - | + | + |
GFP
GFP
PRDX3
PRDX3
Figure S8E

## Slide 20
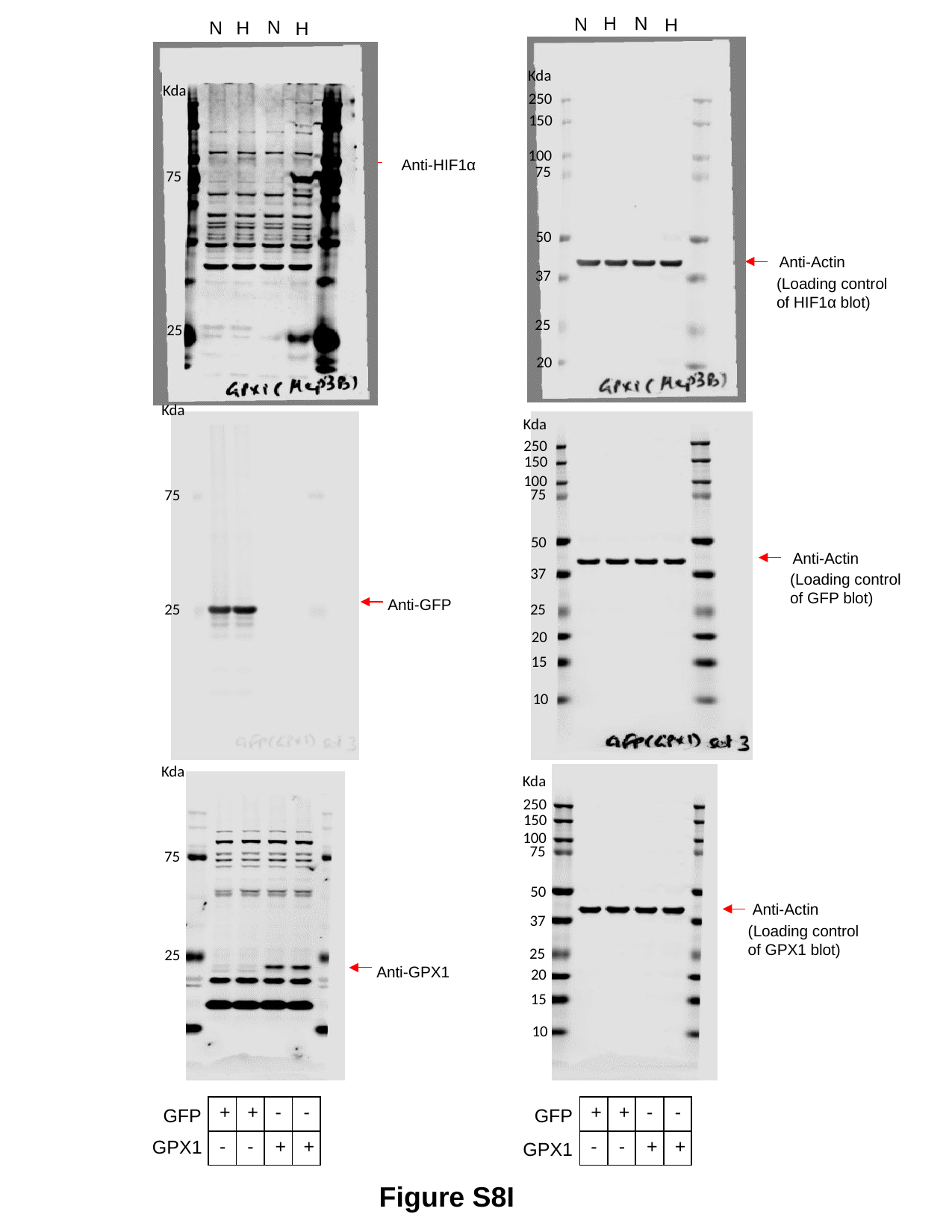

N
H
N
H
N
H
N
H
Kda
Kda
250
150
100
Anti-HIF1α
75
75
50
Anti-Actin
37
(Loading control
of HIF1α blot)
25
25
20
Kda
Kda
250
150
100
75
75
50
Anti-Actin
37
(Loading control
of GFP blot)
Anti-GFP
25
25
20
15
10
Kda
Kda
250
150
100
75
75
50
Anti-Actin
37
(Loading control
of GPX1 blot)
25
25
Anti-GPX1
20
15
10
| + | + | - | - |
| --- | --- | --- | --- |
| - | - | + | + |
| + | + | - | - |
| --- | --- | --- | --- |
| - | - | + | + |
GFP
GFP
GPX1
GPX1
Figure S8I
